# Characterizing genetic and environmental influences on variable DNA methylation using monozygotic and dizygotic twins

**DOI:** 10.1371/journal.pgen.1007544

**Published:** 2018-08-09

**Authors:** Eilis Hannon, Olivia Knox, Karen Sugden, Joe Burrage, Chloe C. Y. Wong, Daniel W. Belsky, David L. Corcoran, Louise Arseneault, Terrie E. Moffitt, Avshalom Caspi, Jonathan Mill

**Affiliations:** 1 University of Exeter Medical School, University of Exeter, Exeter, United Kingdom; 2 Department of Psychology and Neuroscience, Duke University, Durham, NC, United States of America; 3 Social, Genetic & Developmental Psychiatry Centre, Institute of Psychiatry, Psychology & Neuroscience, King’s College London, London United Kingdom; 4 Department of Population Health Sciences, Duke University School of Medicine, Durham, NC, United States of America; 5 Center for Genomic and Computational Biology, Duke University, Durham, NC, United States of America; 6 Department of Psychiatry and Behavioral Sciences, Duke University Medical School, Durham, NC, United States of America; Albert Einstein College of Medicine, UNITED STATES

## Abstract

Variation in DNA methylation is being increasingly associated with health and disease outcomes. Although DNA methylation is hypothesized to be a mechanism by which both genetic and non-genetic factors can influence the regulation of gene expression, little is known about the extent to which DNA methylation at specific sites is influenced by heritable as well as environmental factors. We quantified DNA methylation in whole blood at age 18 in a birth cohort of 1,464 individuals comprising 426 monozygotic (MZ) and 306 same-sex dizygotic (DZ) twin pairs. Site-specific levels of DNA methylation were more strongly correlated across the genome between MZ than DZ twins. Structural equation models revealed that although the average contribution of additive genetic influences on DNA methylation across the genome was relatively low, it was notably elevated at the highly variable sites characterized by intermediate levels of DNAm that are most relevant for epigenetic epidemiology. Sites at which variable DNA methylation was most influenced by genetic factors were significantly enriched for DNA methylation quantitative trait loci (mQTL) effects, and overlapped with sites where inter-individual variation correlates across tissues. Finally, we show that DNA methylation at sites robustly associated with environmental exposures such as tobacco smoking and obesity is also influenced by additive genetic effects, highlighting the need to control for genetic background in analyses of exposure-associated DNA methylation differences. Estimates of the contribution of genetic and environmental influences to DNA methylation at all sites profiled in this study are available as a resource for the research community (http://www.epigenomicslab.com/online-data-resources).

## Author summary

The study of monozygotic (MZ) and dizygotic (DZ) twins provides an opportunity for exploring the extent to which heritable and environmental factors contribute to phenotypic variation in human populations. We exploit the twin study design to explore the factors influencing epigenetic variation between individuals, focussing on DNA methylation, the best-characterized and most stable epigenetic modification. We find that site-specific levels of DNA methylation are more strongly correlated across the genome between MZ than DZ twins. While the average contribution of additive genetic influences on DNA methylation is relatively low, it is notably elevated at sites that are highly variable and have intermediate levels of DNAm, which are most relevant for epigenetic epidemiology. Sites at which variable DNA methylation is strongly influenced by genetic factors are enriched for DNA methylation quantitative trait loci (mQTL) effects, and overlap with sites where inter-individual variation correlates across tissues. Importantly, we show that DNA methylation at sites robustly associated with environmental exposures such as smoking and obesity is also influenced by genetic effects, highlighting the need to control for genetic background in analyses of exposure-associated DNA methylation differences. Finally, we present a searchable database cataloguing the genetic and environmental contributions to variable DNA methylation across the genome (http://www.epigenomicslab.com/online-data-resources).

## Introduction

The study of twins provides an opportunity for exploring the extent to which heritable and environmental factors contribute to phenotypic variation in human populations [1]. By comparing concordance rates between monozygotic (MZ) and dizygotic (DZ) twins it has been shown that most human traits are, at least in part, influenced by DNA sequence variation [2]. The fact that genetically-identical MZ twins exhibit phenotypic differences indicates that non-sequence based factors, usually attributed to the environment, also contribute to phenotypic variation. Increasing knowledge about the biology of the genome has stimulated interest in the role of epigenetic processes—acting to developmentally regulate gene expression via modifications to DNA, histone proteins, and chromatin—in mediating phenotypic variation across the life-course. Growing evidence identifies epigenetic differences between MZ twins [3], and epigenetic variation is associated with a range of health and disease phenotypes [4].

The primary focus of epigenetic epidemiology is on DNA methylation, the best-characterized and most stable epigenetic modification, which is assumed to influence gene expression via the disruption of transcription factor binding and the attraction of methyl-binding proteins that initiate chromatin compaction and gene silencing. DNA methylation can be influenced by both environmental and genetic factors, meaning that careful study design in epigenome-wide association studies (EWAS) is important to minimize the influence of confounders and false positives [4, 5]. There is evidence that certain exposures–for example, to tobacco smoke [6–8], dietary factors [9, 10] and psychosocial stress [11, 12]–are associated with changes in DNA methylation at specific sites across the genome. Likewise, studies have identified associations between DNA sequence variation and DNA methylation at sites across the genome [13–16]; these DNA methylation quantitative trait loci (mQTLs) often overlap with DNA variants associated with levels of gene expression (expression quantitative trait loci; eQTLs)[14, 17], providing a potential mechanism linking genetic variation to gene regulation.

Researchers are starting to exploit the twin study design to further explore the extent to which epigenetic variation between individuals is influenced by genetic and environmental factors. Recent studies have shown that DNA methylation profiles are more similar between related individuals than unrelated individuals, with greater concordance between MZ than DZ twins [18, 19]. Twin studies suggest that the proportion of variance in DNA methylation explained by genetic factors is on average low (typically 5–19%) at the majority of sites that have been tested across the genome [19–21]. Importantly, however, the contribution of genetic and environmental factors to DNA methylation varies at sites across the genome, and potentially differs as a function of tissue, age and sex [21]. Studies investigating associations between DNA methylation and phenotypic variation, should not dismiss the impact that genetic variation may have on their results.

Here we report findings about the genetic and environmental architecture of DNA methylation in whole blood at age 18 years using samples collected from the Environmental Risk (E-Risk) Longitudinal Twin Study, a representative birth cohort of young-adult twins based in the UK. Young adulthood is a life stage when people show great variation in health risk behaviors and exposures that have been hypothesized to alter an individuals’ epigenome. Our goal was to characterize the genetic and environmental determinants of variation in DNA methylation in order to inform future methylomic analyses of complex traits. By analyzing a sample where all twin pairs provided a whole blood sample at the same age, we minimize the confounding influence of age-associated variation.

We first used structural equation modeling to calculate the proportion of variance in DNA methylation explained by additive genetic (A), shared environmental (C) and unshared (or unique) environmental (E) factors at sites across the genome. Second, we explored whether the contribution of genetic and environmental influences on DNA methylation differs depending upon the level and/or variability in DNA methylation at individual sites. Third, we assessed how genetic and environmental influences on DNA methylation differ as a function of genic location, describing the factors influencing variable DNA methylation across gene regulatory regions. Fourth, we tested the hypothesis that sites characterized by highly heritable levels of DNA methylation are enriched for known mQTL effects. Fifth, we explored the extent to which biological phenotype estimates derived from DNA methylation data itself (e.g. age and blood cell proportions) are influenced by genetic and environmental factors, in addition to estimating the genetic and non-genetic contribution to levels of DNA methylation at sites robustly associated with specific environmental exposures (e.g. tobacco smoking and obesity). Finally, as a resource for the research community, we present a searchable database cataloguing the genetic and environmental contributions to variable DNA methylation across all sites on the Illumina 450K array (http://www.epigenomicslab.com/online-data-resources).

## Results

### Site-specific levels of DNA methylation are more strongly correlated between MZ twins than DZ twins, especially at sites with variable and intermediate levels of DNA methylation

We quantified genome-wide patterns of DNA methylation using the Illumina Infinium HumanMethylation450 BeadChip (“450K array”) in DNA samples isolated from whole blood collected at age 18 years from members of the E-Risk cohort [22]. After implementing a stringent quality control (QC) pipeline (see **Methods**), our final sample included 426 MZ twin pairs (48.5% female) and 306 DZ twin pairs (49.2% female) (1,464 individuals, a representative 65.6% of participants, see **Methods**).

**Fig 1:**
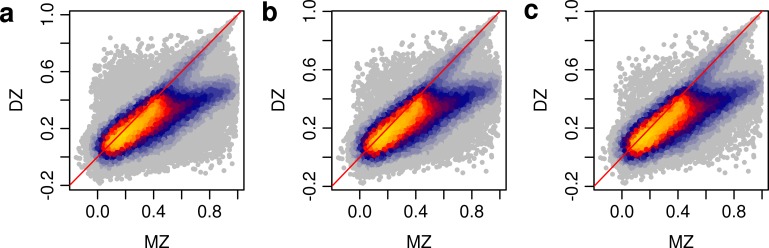
Monozygotic (MZ) twins are more concordant than dyzygotic (DZ) twins for DNA methylation at the majority of autosomal sites tested. (**a**) Scatterplot showing the correlation of DNA methylation values within MZ twin-pairs (x-axis) and DZ twin-pairs (y-axis) for all 420,857 autosomal Illumina 450K array sites passing our stringent quality control (QC) pipeline. MZ twin pairs are more similar to each other than DZ twin pairs for 277,077 (65.8%) of sites (sign-test P = 1.98x10-323). The elevated concordance of DNA methylation in MZ twins compared to DZ twins is more pronounced amongst both (**b**) “variable” sites (77.6%, sign-test P = 1.48x10-323) and (**c**) sites with intermediate levels of DNAm (83.0%, sign-test P = 9.88x10-324). The red diagonal line indicates x = y. The color indicates the density of data points ranging from yellow (highest) to grey (lowest).

We first assessed the profile of DNA methylation across all 420,857 autosomal 450K array sites included in our final dataset. As expected, these ‘global’ patterns of DNA methylation were highly stable between individuals (**S1 Fig)**, although the average inter-individual correlation of DNA methylation across sites was significantly higher between siblings than between unrelated individuals (P = 2.20x10^-223^). MZ twin pairs were more similar to each other than DZ twin pairs for the majority of sites tested (N = 277,077 (65.8%), sign test P = 1.98x10^-323^) (**Fig 1**); the average sibling correlation across the 420,857 sites was significantly higher for MZ twin-pairs than for DZ pairs (mean MZ sibling correlation = 0.996, mean DZ sibling correlation = 0.995, P = 1.29x10^-34^). The magnitude of this difference was relatively small, reflecting the fact that most autosomal 450K array probes are characterized by consistently high (>80%) or low (<20%) levels of DNA methylation, and minimal inter-individual variation. We therefore estimated sibling correlations for the subset of autosomal DNA methylation sites we defined as either “variable” (i.e. those where the range of DNA methylation values for the middle 80% of individuals was greater than 5%; N = 214,991 sites (51.1%)) or with intermediate levels of DNAm (i.e. those where the mean level of DNA methylation was between 20% and 80%; N = 131,728 sites (31.3%)) (see **Methods**). These probe subsets were not distinct; the majority (N = 127,935 (97.1%)) of DNA methylation sites with intermediate levels of DNAm were also classed as “variable” (**S2 Fig)**. The elevated concordance in DNA methylation levels in MZ twins compared to DZ twins was more pronounced amongst both “variable” sites (number of sites at which MZ twin pairs are more similar to each other than DZ twin pairs = 166,783 (77.6%), sign test P = 1.48x10^-323^) and sites with intermediate levels of DNAm (number of sites at which MZ twin pairs are more similar to each other than DZ twin pairs = 109,303 (83.0%), sign test P = 9.88x10^-324^) (**Fig 1**). Furthermore, there was an overall elevated average sibling similarity for DNA methylation levels in MZ twins compared to DZ twins amongst both “variable” DNA methylation sites (mean MZ sibling correlation = 0.989, mean DZ sibling correlation = 0.985, P = 3.92x10^-38^) and DNA methylation sites with intermediate levels of DNAm (mean MZ sibling correlation = 0.979, mean DZ sibling correlation = 0.968, P = 1.55x10^-39^) (**S1 Fig)**, consistent with findings from previous twin studies of DNA methylation in whole blood [21, 23].

### Autosomal DNA methylation is predominantly influenced by non-shared environmental factors

**Fig 2:**
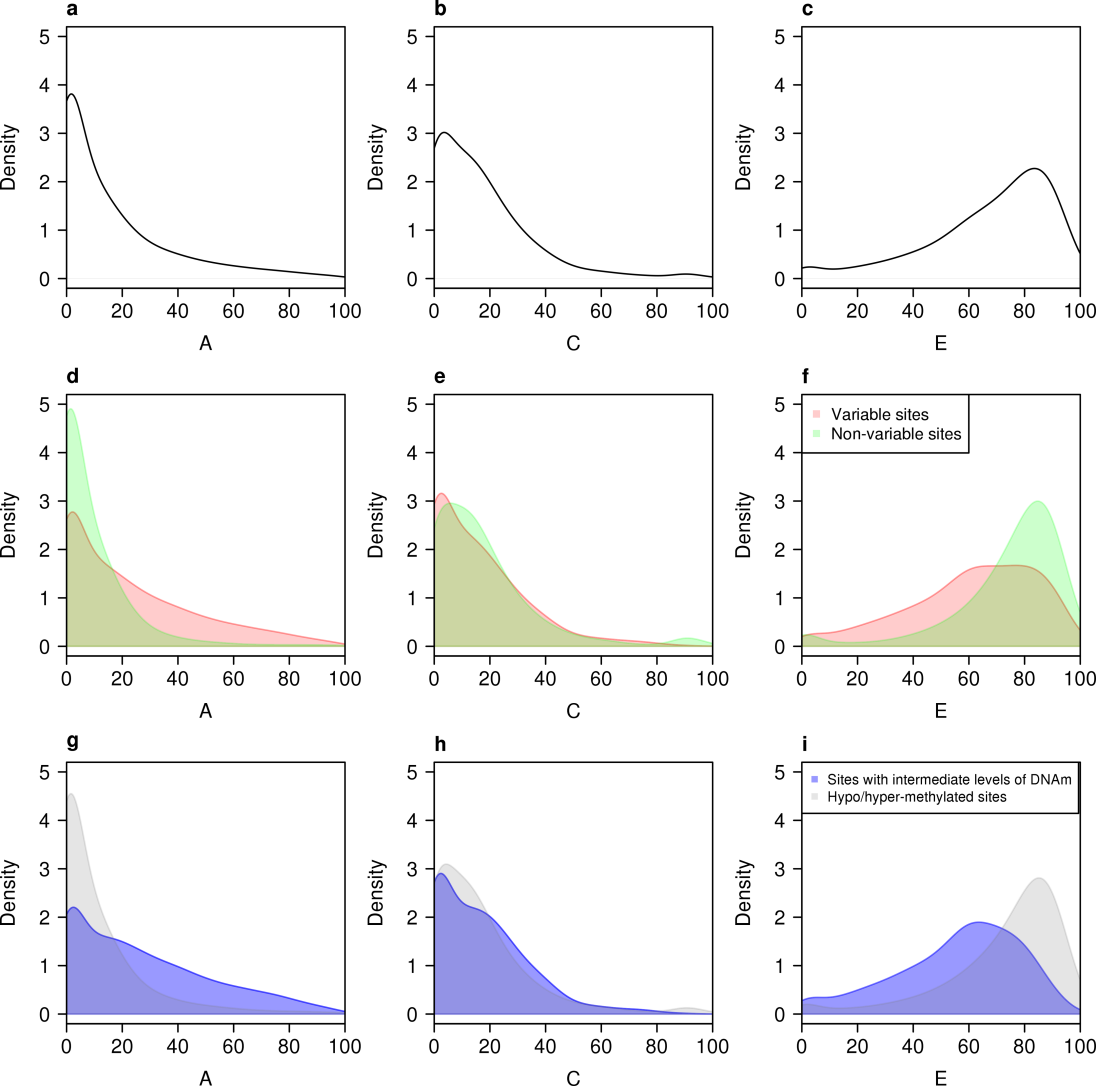
**The proportion of variance in DNA methylation explained by additive genetic effects (A), shared environmental effects (C) and unshared (or unique) environmental effects (E) across autosomal sites.** Panels **a-c** show density distributions for estimates of A, C, and E across all 420,857 autosomal DNA methylation sites. At the majority of autosomal sites, environmental factors contribute more to the observed variance in DNA methylation than additive genetic factors. We observe significantly higher average heritability estimates for DNA methylation across the subset of DNA methylation sites defined as “variable” (**d-f**) (mean A = 23.0% (SD = 23.8%); Mann Whitney P < 2.2x10^-16^) and (**g-i**) “sites with intermediate levels of DNAm” (mean A = 27.3% (SD = 24.6%); Mann Whitney P < 2.2x10^-16^).

**Fig 3:**
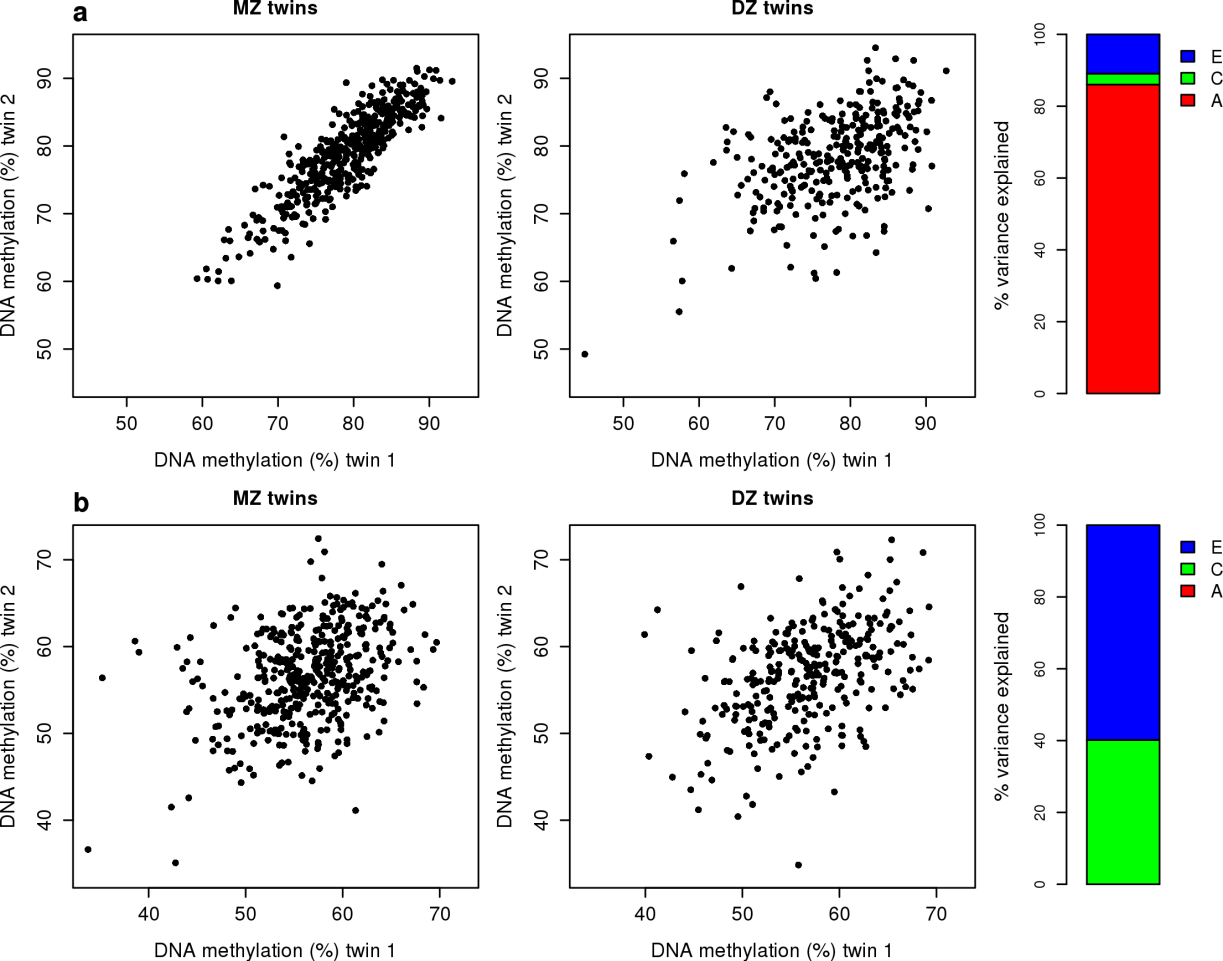
Examples of autosomal sites at which DNA methylation is differentially influenced by additive genetic and environmental factors. (**a**) An example of a site (cg00002033) at which DNA methylation is highly heritable. The scatterplot shows DNA methylation values in MZ (left panel) and DZ (right panel) twin pairs. Each point represents an individual twin-pair. At this site, the correlation of DNA methylation is markedly higher in MZ twins (r = 0.882) compared to DZ twins (r = 0.484). Structural equation modelling highlights that DNA methylation at this site is strongly influenced by additive genetic effects (A = 79.7%, C = 8.53%, E = 11.8%). (**b**) An example of a site (cg00000289) at which DNA methylation is not strongly influenced by genetic factors. The scatterplot shows DNA methylation values in MZ (left panel) and DZ (right panel) twin pairs. Each point represents an individual twin-pair. At this site, the correlation of DNA methylation is similar in both MZ twins (r = 0.363) and DZ twins (r = 0.449) highlighting that DNA methylation is strongly influenced by the environment (A = 0%, C = 40.2%, E = 59.8%). MZ and DZ correlations for DNA methylation across all sites on the Illumina 450K array can be visualized at http://www.epigenomicslab.com/online-data-resources.

DNA methylation is widely hypothesized to be a mechanism by which both heritable and environmental factors can influence the regulation of gene expression and function, but little is known about the extent to which DNA methylation at specific sites is actually influenced by genetic and non-genetic factors. We fitted structural equation models to estimate the proportion of variance in DNA methylation explained by additive genetic effects (A), shared environmental effects (C) and unshared (or unique) environmental effects (E) across all 420,857 autosomal sites (see **Methods**) (**Table 1**). The average contribution of additive genetic effects across all DNA methylation sites was relatively low but highly variable (mean A = 15.9% (SD = 20.8%)) (**Fig 2A–2C**); our mean estimate of heritability was slightly below that observed in previous studies of older and more variably-aged twin-pairs [19, 23]. On average, the largest contribution to variation in DNA methylation was attributable to unique environmental influences, which also indexes measurement error (mean E = 67.4% (SD = 22.9%)). The mean estimate for common environmental influences across all 420,857 autosomal sites was similar to that for additive genetic effects (mean C = 16.7% (SD = 17.8%)). These data highlight that variation in DNA methylation can be influenced by both genetic and non-genetic factors, and that the relative importance of these influences differs across sites in the genome. Because whole blood is a heterogeneous tissue, we derived blood cell proportion estimates for each sample using the DNAm data (see **Methods**) and repeated our structural equation modelling in an attempt to explore the effects of cellular heterogeneity on heritability estimates of DNAm. Including derived blood cell-types as a covariate in our model did not change the pattern of results (mean A = 16.5% (SD = 21.2%), mean C = 12.6% (SD = 13.7%), mean E = 71.0% (SD = 20.9%)) (**S3 Fig**), with estimates for genetic and environmental influences on DNAm across sites being highly correlated across both models (**S4 Fig**). **Fig 3** shows examples of sites at which the level of DNA methylation was influenced by a high (**Fig 3A**) and low (**Fig 3B**) additive genetic component. MZ and DZ twin correlations and estimates for A, C, and E for all Illumina 450K array sites are available as an online resource at http://www.epigenomicslab.com/online-data-resources).

**Table 1:** The contribution of additive genetic and environmental factors to levels of DNA methylation. Shown are the results from structural equation models to estimate the mean proportion of variance in DNA methylation explained by additive genetic effects (A), shared environmental effects (C) and unshared (or unique) environmental effects (E) across Illumina 450K probes. Results are presented separately for DNA methylation sites located on the autosomes and X-chromosome, and stratified by whether they have intermediate levels of DNAm and/or are “variable”.

		*A*		*C*		*E*	
***Autosomes (all twins)***							
	N probes	Mean	SD	Mean	SD	Mean	SD
**All**	420,857	15.9%	20.8%	16.7%	17.8%	67.4%	22.9%
**Intermediate levels of DNAm**	131,728	27.3%	24.6%	16.8%	16.5%	55.9%	22.3%
**Variable**	214,991	23.0%	23.8%	15.9%	16.8%	61.1%	23.2%
***X Chromosome (female twins)***							
**All**	9,896	30.2%	17.0%	13.6%	20.4%	56.3%	22.1%
**Intermediate levels of DNAm**	7,911	32.1%	15.6%	14.8%	21.5%	53.1%	21.5%
**Variable**	9,127	31.3%	16.5%	13.5%	20.5%	55.3%	21.7%
***X Chromosome (male twins)***							
**All**	9,896	12.1%	17.5%	17.7%	24.5%	70.2%	25.6%
**Intermediate levels of DNAm**	2,778	18.8%	20.7%	16.1%	16.7%	65.1%	20.0%
**Variable**	5,377	15.0%	19.4%	15.5%	18.5%	69.4%	22.1%

### Additive genetic influences on DNA methylation are highest at highly variable sites and sites with intermediate levels of DNAm

**Fig 4:**
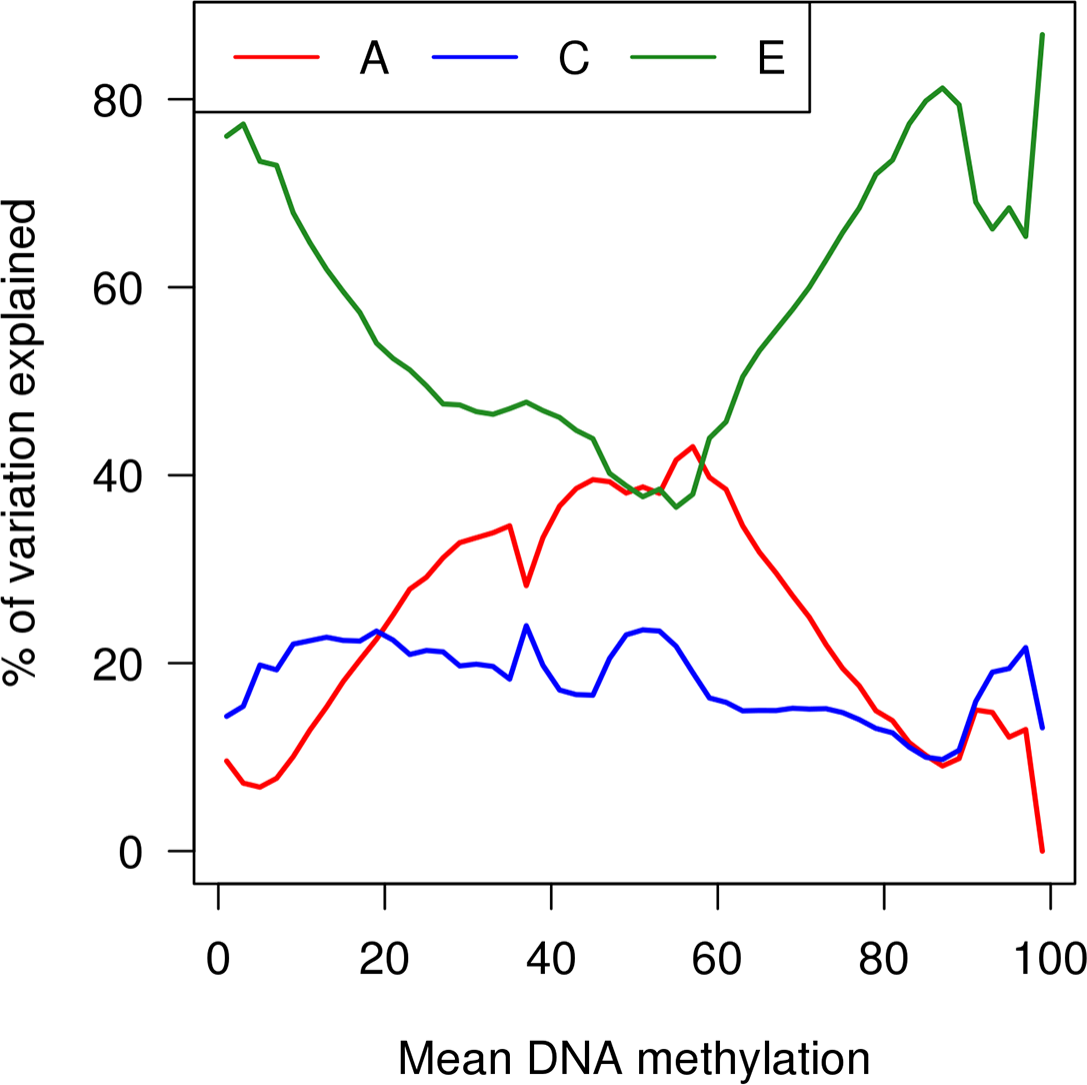
The contribution of genetic and environmental influences on DNA methylation at autosomal sites differs as a function of average DNA methylation level at that location. Shown are estimates of additive genetic effects (A), shared environmental effects (C) and non-shared (or unique) environmental effects (E) against mean DNA methylation level. The most heritable sites are characterized by intermediate levels of DNA methylation.

We next tested the hypothesis that DNA methylation at sites which are “variable” or have intermediate levels of DNAm is more highly heritable than other sites in the genome. Average additive genetic influences on DNA methylation were markedly higher at “variable” autosomal sites compared to non-variable sites (mean A = 23.0% (SD = 23.8%), Mann Whitney P < 2.2x10^-16^) (**Fig 2D** and **S5 Fig)**. Likewise, additive genetic influences on DNA methylation were significantly higher at autosomal sites with intermediate levels of DNAm compared to hyper/hypo-methylated sites (mean A = 27.3% (SD = 24.6%), Mann Whitney P < 2.2x10^-16^), with a striking inverted U-shaped relationship between the level of DNA methylation and the extent to which it was influenced by additive genetic factors (**Fig 2G** and **Fig 4**). In contrast, the influence of non-shared environmental factors was significantly lower at “variable” autosomal sites compared to non-variable sites (mean E = 61.1% (SD = 23.2%); Mann-Whitney P < 2.2x10^-16^) (**Fig 2F**). The contribution of non-shared environmental factors was also lower at autosomal sites with intermediate levels of DNAm compared to either hyper- or hypo-methylated sites (mean E = 55.9% (SD = 22.3%); Mann-Whitney P < 2.2x10^-16^) (**Fig 2I**); there is a U-shaped relationship between the mean level of DNA methylation and the proportion of variance explained by unique environmental effects; the smallest contribution of E was observed at sites that were 56–58% methylated (**Fig 4**). Shared environmental influences were fairly stable and not strongly affected by either the average variability or level of DNA methylation. These results are important because they suggest that the effects of genetic variants associated with phenotypic differences are likely to be more pronounced at DNA methylation sites that are variable or have intermediate levels of DNAm compared to hypo- or hyper methylated sites, which are more stable in the population and often associated with cell-type-specific patterns of gene expression.

### Genetic influences on DNA methylation are not evenly distributed across genic regions

Although DNA methylation across CpG-rich promoter regions is often associated with the repression of gene expression, recent work has revealed a more nuanced relationship between DNA methylation and transcription that is frequently dependent on genomic context [24]. DNA methylation in the gene body, for example, can be a marker of active gene transcription [25, 26], potentially playing a role in regulating alternative splicing and isoform diversity. Given these contextual differences, we tested whether genetic and environmental contributions to variable DNA methylation differ across genomic domains. As DNAm sites located in specific gene features differ in their variability, these analyses focused on our subset of “variable” DNAm sites to prevent any potential confounding. First, we used a sliding-window approach to examine how the proportion of variation in DNA methylation explained by genetic and environmental influences changes across a canonical gene region (**S6 Fig)**. There was a peak in the contribution of shared environmental influences in the vicinity of the transcription start site (TSS), accompanied by a reduction in the contribution of non-shared environmental influences. The contribution of additive genetic factors to variation in DNA methylation was highest at sites located immediately upstream of the TSS, and also in a region spanning ~5 kilobases (kb) downstream of the transcription termination site. Second, we tested the extent to which DNA methylation levels at sites annotated to specific genic features (**S7 Fig)** and CpG island features (**S8 Fig)** were influenced by additive genetic or environmental factors. Variation in DNA methylation at sites in the immediate vicinity of a TSS, or annotated to a first exon or CpG island, were associated with significantly higher additive genetic and shared environmental influences (all Mann-Whitney P < 2.2x10^-16^) (**S1 Table**). Given the presumed importance of promoter-region DNA methylation in regulating gene expression, these observations suggest that both environmental and genetic factors can influence transcriptional regulation via DNA methylation at these promoter-region locations and that, on average, the effects across features are relatively consistent. Finally, we investigated how the influence of genetic and environmental factors on DNAm varies across regulatory features and chromatin states defined by ChromHMM using ENCODE ChIP-seq data for a well-characterized lymphoblastoid cell line (GM12878) (**S9 Fig**). This analysis revealed higher levels of additive genetic effects on DNAm at sites in insulators (mean A = 23.0%, SD = 24.2%), repressed (mean A = 19.6%, SD = 21.4%) and repetitive/CNV regions (mean A = 24.8–27.0%, SD = 25.8–26.2%), with moderate levels of heritability in enhancer regions (mean A = 17.5–19.1%, SD = 20.9–22.1%). In contrast, DNAm at sites located in promoters is characterized by an increased proportion of variance explained by unique environmental factors (E = 65.6–67.8%, SD = 22.3–23.2%) reflecting the genic annotation results above.

### Sites at which variable DNA methylation is strongly influenced by additive genetic factors are significantly enriched for mQTL effects

Given that epigenetic epidemiology aims to understand both the causes and phenotypic consequences of differential DNA methylation, we focused our subsequent analyses on the subset of 214,991 “variable” autosomal DNA methylation sites. Hypothesizing that the majority of heritable DNA methylation sites identified in this study are influenced by common genetic variation, we tested whether they were enriched for mQTL effects, i.e. common genetic variants known to be robustly associated with DNA methylation at specific sites [13, 27, 28]. We used a large mQTL database generated by our group on an independent set of whole blood samples [29] to identify overlap with the most highly heritable DNA methylation sites (defined as those with A > 0.8; n = 4,882) identified in the E-Risk cohort. DNA methylation at 84.7% of these sites was significantly associated with at least one common genetic variant using a stringent mQTL threshold (P < 1x10^-8^) (**S2 Table**); this represented a highly significant enrichment for mQTL effects (P < 2.2x10^-16^) compared to less-heritable DNA methylation sites (defined as those with A < 0.8), amongst which only 24.5% were associated with a mQTL variant. Of note, mQTL effect sizes vary as a function of the mean level of DNAm. Sites with intermediate levels of DNAm are associated with larger mQTL effects (mean = 4.99% change in methylation per allele (SD = 3.61%)) compared to sites characterized as being hyper- or hypo-methylated (mean = 3.56% change in methylation per allele (SD = 2.79%); Mann-Whitney P < 2.2x10^-16^); this parallels the relationship observed between the level of DNAm and the influence of additive genetic factors (**S10 Fig**). These findings suggest that the incorporation of common SNP data into epigenome-wide association studies (EWAS) will facilitate understanding about the contribution of genetic and non-genetic factors to trait-associated methylomic variation. An example of a highly heritable DNA methylation site (cg02573566, A = 96.9%) that was also associated with an mQTL SNP (rs11548104, P = 5.95x10^-179^) is shown in **S11 Fig**. Of note, observed DNA methylation at highly heritable sites for which we did not detect an mQTL (15.3%) does not necessarily signal false positives as these sites may be associated with rare variation or larger structural variants not assessed in existing mQTL databases. mQTLs influencing levels of DNA methylation at highly heritable sites were associated with larger effects (mean change in DNA methylation per allele = 6.77% (SD = 4.48%)) compared to all identified mQTLs (mean change in DNA methylation per allele = 3.03% (SD = 3.10%)) (P = < 2.2x10^-16^). Across all autosomal 450K array sites, there was a relatively linear relationship between the contribution of genetic influences to variation in DNA methylation and the proportion of sites influenced by an mQTL (**S12 Fig**). In contrast, the proportion of DNA methylation sites that were associated with an mQTL decreased as the contribution of the common or unique environment to levels of DNA methylation increased. Taken together, these findings confirm our hypothesis that DNA methylation at the majority of highly heritable sites is directly influenced by common genetic variants.

### DNA methylation sites at which inter-individual variation is correlated across tissues are characterized by higher levels of heritability

**Fig 5:**
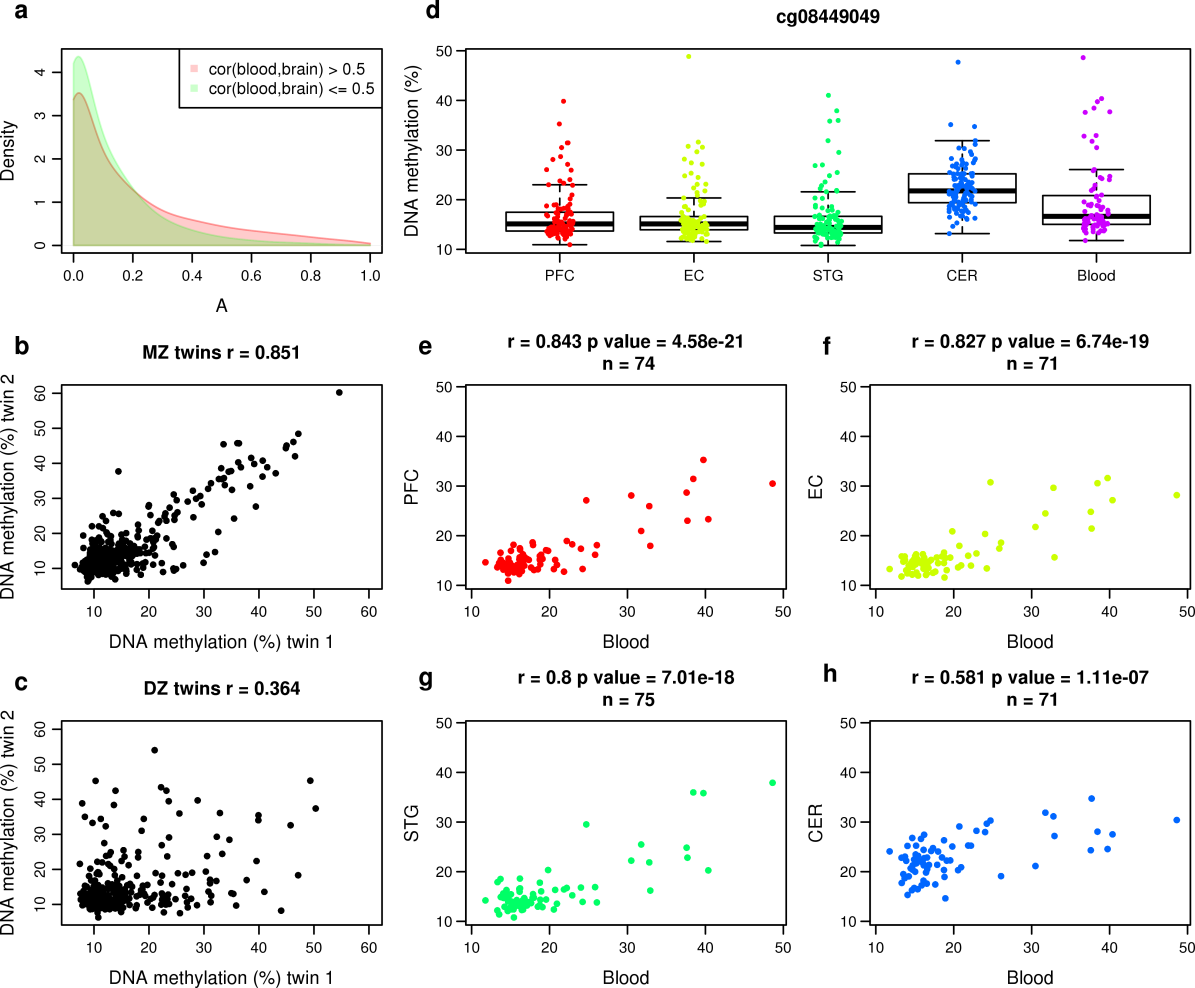
DNA methylation sites at which inter-individual variation is correlated across tissues are characterized by higher levels of heritability. (**a**) A density plot of heritability estimates for DNA methylation at sites split by the extent to which DNA methylation co-varies between whole blood and the prefrontal cortex using data from Hannon et al (2015). Heritability is significantly higher in probes where the cross-tissue covariation in DNA methylation is high (r^2^ > 0.5, red). (**b-h**) An example of a probe (cg08449049) at which DNA methylation is strongly influenced by additive genetic effects and also co-varies between blood and multiple regions of the human brain. Shown are scatterplots of DNA methylation values at cg08449049 for (**b**) MZ (corr = 0.851) and **c)** DZ (corr = 0.364) twin pairs. Each point represents an individual twin-pair. (**d**) A boxplot of the distribution of DNA methylation levels at cg08449049 in blood and four brain regions (PFC = prefrontal cortex, EC = entorhinal cortex, STG = superior temporal gyrus, CER = cerebellum) from the same individual donors using data generated by Hannon et al (2015). (**e-h**) Scatterplots of the DNA methylation values in blood against the DNA methylation values in each of the four brain regions showing that there is significant covariation across tissues.

Epigenetic association studies of phenotypes where the presumed tissue of interest is challenging to obtain (e.g. regions of the human brain) typically use more accessible peripheral tissues (e.g. whole blood) under the premise that variation identified in these ‘proxy’ tissues potentially mirrors that in the disease-relevant tissue. We have previously shown, however, that whole blood generally has limited utility for inferring inter-individual variation in multiple regions of the human brain [30]. Where there is significant co-variation between two tissues from the same individual, we hypothesized that this is likely to reflect genetic effects on DNA methylation that are manifest across tissues. We used the matched blood and brain DNA methylation datasets, previously generated by our group [30], to confirm that DNA methylation at sites characterized by high inter-individual co-variation across tissues from the same individual is more likely to be influenced by heritable factors. For example, we observed a striking increase in the heritability of DNA methylation at the subset of sites at which inter-individual variation in our prior sample was strongly correlated between whole blood and the prefrontal cortex (covariation between blood and prefrontal cortex > 0.5, N = 9,212 sites) compared to those at which variation was less correlated across tissues (median A = 71.1% vs 14.7%, Mann-Whitney P < 2.2x10^-16^) (**Fig 5A**). Overall, there was a strong positive correlation (r = 0.500) between the additive genetic contribution to DNA methylation and tissue co-variation (blood vs prefrontal cortex) across variably methylated sites (**S13 Fig**), confirming that sites at which DNA methylation co-varies across tissues are more likely to be influenced by heritable factors. Similar effects were seen for the other brain regions profiled from the same individual donors (entorhinal cortex, superior temporal gyrus and cerebellum). An example of a site where DNA methylation significantly covaries between whole blood and brain, and is strongly influenced by additive genetic effects, is shown in **Fig 5B–5H**. These results are important because they suggest that concerns regarding tissue-specific effects on DNA methylation are likely to be more relevant for studies of environmentally-induced variation as compared to analyses of genetic influences on DNA methylation.

### Genetic influences on DNA methylation at sites on the X chromosome are also highest at sites characterized by intermediate levels of DNAm and high variability

Because DNA methylation on the X-chromosome differs markedly between males and females–primarily due to its role in regulating the dosage compensation of X-linked genes (see **S14 Fig**)—the analyses presented above focused solely on autosomal DNA methylation sites. We next estimated the proportion of variance in DNA methylation explained by additive genetic effects, shared environmental effects and non-shared (or unique) environmental effects for probes on the X chromosome in male and female twins separately (male: 156 DZ twin pairs, 219 MZ twin pairs; female: 150 DZ twin pairs, 207 MZ twin pairs) (**Table 1**). As hypothesized, X-chromosome DNA methylation was much more variable in females than males; the majority (N = 9,127, 92.2%) of X-linked DNA methylation sites met our criteria for being “variable” in females compared to just over half (N = 5,377, 54.3%) in males. Most DNA methylation sites classified as “variable” in males were also found to be “variable” in females (N = 5,195; 96.6%). In males, the contribution of genetic and environmental influences to DNA methylation at sites on the X-chromosome was similar to that observed at autosomal loci; for males, more variation was attributed to unique environmental influences (mean = 69.4%, SD = 22.1%) than shared environmental (mean = 15.5%, SD = 18.5%) or additive genetic (mean = 15.0%, SD = 19.4%) influences (**S15 Fig**). Furthermore, the influence of additive genetic factors on male X-chromosome DNA methylation was highest at sites characterized by either “intermediate levels of DNAm” (**S16 Fig**) or “variable” levels of DNA methylation (**S17 Fig**). Although most variance in X-chromosome DNA methylation in females could also be attributed to the unique environment (mean E = 55.3%, SD = 21.7%), the average contribution of additive genetic factors (mean A = 31.3%, SD = 16.5%) was significantly higher compared to that observed at autosomal sites (P < 2.2x10^-16^) and X-linked sites in males (P < 2.2x10^-16^) (**Table 1)**. While the influence of genetic and environmental factors on DNA methylation across sites on the X-chromosome was positively correlated between males and females (**S18 Fig**), with the strongest correlation seen for unique environmental influences (r = 0.381), there was some notable heterogeneity. A number of sites, for example, were characterized by sex-specific additive genetic influences on DNA methylation (**S19 Fig** and **S20 Fig**). These results are interesting as they could potentially mediate observed sex differences for certain inherited phenotypes. This heterogeneity of effects may also have negative effects on power for statistical significance in EWAS analyses that combine males and female samples to analyze sites on the X chromosome; to truly disentangle genetic and environmental effects on X-chromosome DNA methylation it is important to analyze the sexes separately. Finally, we examined the genetic and environmental contribution to variable DNAm across regions annotated to the small subset of genes known to escape X-chromosome inactivation (XCI) in females. Using RNA-seq data from the GTEx consortium [31] we selected DNAm sites annotated to the 5’UTR or within 1500 bp of the transcription start site of genes highlighted as escaping XCI. As expected, the distribution of DNAm across sites annotated to genes escaping XCI is dramatically different to other X-chromosome sites in females, with a striking enrichment of hypomethylated loci. Despite the differences in levels of DNAm associated with genes escaping XCI, the contribution of additive genetic and environmental influences on DNAm at these sites is broadly comparable to that seen at sites across the X-chromosome in females (**S21 Fig**).

### Estimates of chronological age and blood cell proportions derived from DNA methylation data are influenced by both genetic and environmental effects

A number of classifiers can be used to derive estimates of biological phenotypes including age (DNAmAge) [32] and the proportion (or abundance) of different cell types present in whole blood [32–34] from DNA methylation data. These estimates are useful because they can be incorporated as covariates in EWAS analyses when empirical measures are missing, or used as interesting variables in their own right in epidemiological analyses [35–37]. We examined the twin correlations for each of these derived variables (**S22 Fig**) and estimated the contribution of additive genetic and environmental influences to these measures by comparing MZ and DZ twins (**S23 Fig**). The mean predicted DNAmAge of samples from participants in this study was 20.7 years (SD = 4.10 years), slightly higher and more variable that the actual age at sampling (mean = 18.4 years; SD = 0.37 years). As DNAmAge is associated with actual chronological age, age acceleration is typically calculated as the residual from a linear regression model of predicted age against reported age. Although the limited age variation in our sample provides limited power for structural equation modelling, we found that DNAmAge acceleration was characterized by an additive genetic contribution of 36.7%, with 42.8% and 20.5% of the variance explained by common environmental and unique environmental influences, respectively. This heritability estimate is lower than the 100% reported previously for age acceleration in a smaller set of newborns but comparable to the 39% reported for adult twin pairs (45–75 years old) [32]. The contribution of additive genetic and environmental influences differed dramatically across the predicted cellular heterogeneity variables, with heritability estimates ranging from 0% (for CD8 T cells and granulocytes) to 47.0% (for CD8+CD28-CD45RA- T cells) (**S3 Table**). For seven of the ten derived cell estimates, the largest proportion of variance was attributed to the influence of unique environmental factors. B cells had the largest proportion of variance estimated as being explained by common environmental factors (52.1%), and naïve CD8 T cells and natural killer cells had the largest proportion explained by genetic factors (at 42.1% and 40.0%, respectively). Comparison between these results and those for empirically-measured cell abundance data is not straightforward as in many cases the estimated cellular composition represents a proportion rather than abundance. Although, there is contradictory evidence in the literature about whether variation in specific blood cell types is more influenced by genetic or environmental factors[38–41], our results are consistent with reports that T cells have higher heritability estimates than B cells [38, 41].

### DNA methylation at sites robustly associated with exposure to tobacco smoking and body mass index (BMI) is strongly influenced by additive genetic effects

**Fig 6:**
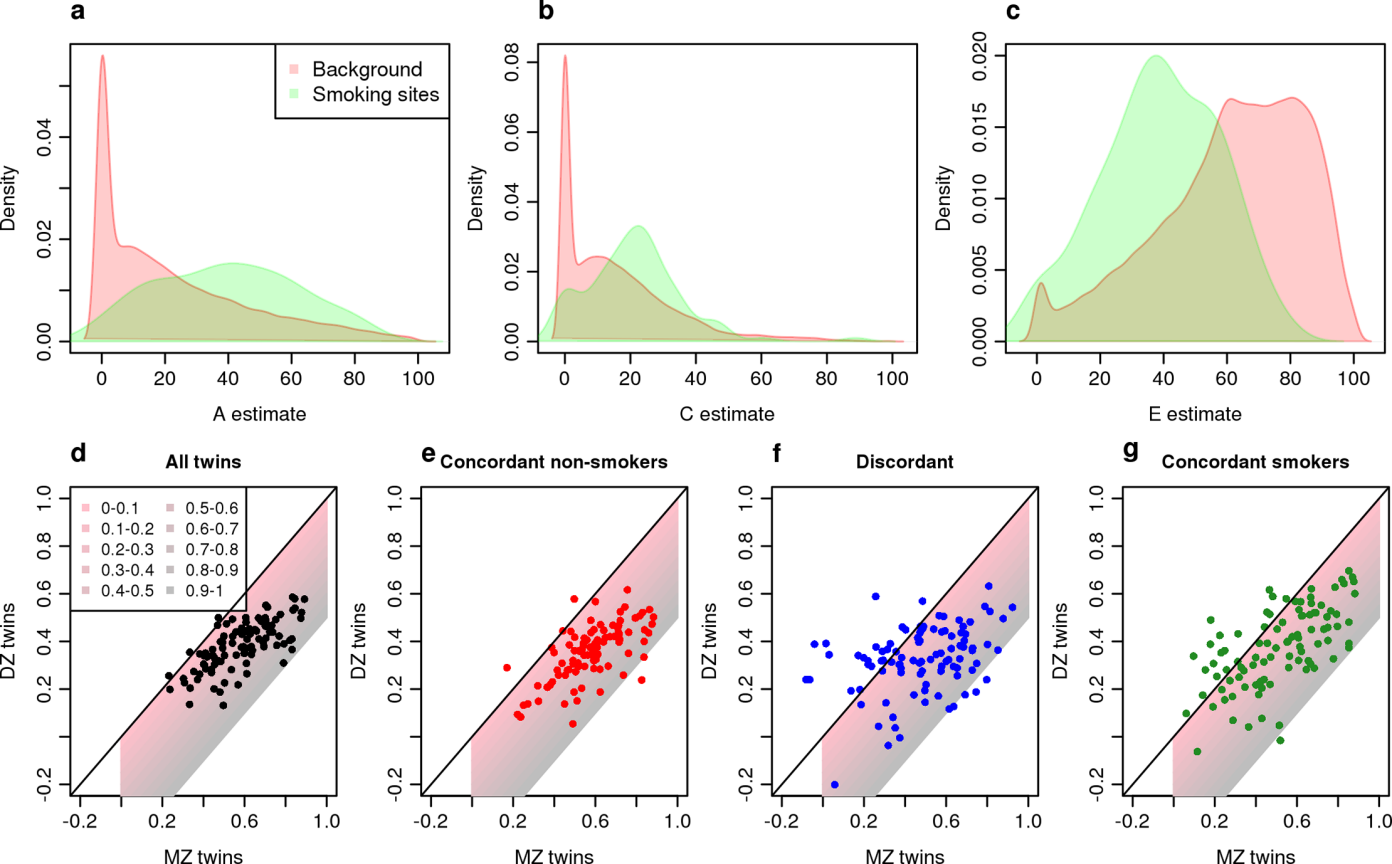
DNA methylation at sites associated with tobacco smoking is strongly influenced by additive genetic factors. Shown is a series of density plots for estimates of (**a**) additive genetic effects (A), (**b**) shared environmental effects (C) and (**c**) non-shared environmental effects (E) at 97 differentially methylated positions (DMPs) associated with smoking (green). Also shown are density plots for A, C and E at ‘background’ sites not associated with smoking (red). Shown below is a series of scatterplots showing the correlation in DNA methylation between MZ twins (x-axis) against DZ twins (y-axis) for sites associated with smoking in (**d**) all twins, (**e**) concordant non-smokers (n = 503 twin-pairs), (**f**) twins discordant for smoking status (n = 123 twin-pairs) and (**g**) concordant smokers (n = 106 twin-pairs). The shaded area on each plot indicates the heritability estimate (using Falconer’s formula) for each site.

Several environmental exposures have been robustly associated with differences in DNA methylation at specific sites across the genome, although the extent to which these relationships are potentially confounded by genetic influences is not known. We first examined whether variation in DNA methylation at sites associated with tobacco smoking—an exposure known to be characterized by robust and reproducible effects on DNA methylation [6, 7, 42, 43]–is also influenced by additive genetic factors. Using the extended E-Risk dataset including singletons (i.e. individuals whose co-twin did not contribute to our DNA methylation dataset), we performed an EWAS of tobacco smoking, identifying 97 differentially methylated positions (DMPs) (P < 1x10^-7^) (**S4 Table**) that are highly consistent with findings from previous studies of smoking in adults [44] (**S24 Fig**). We next examined the extent to which DNA methylation at these sites was influenced by genetic and environmental factors. We identified a strong genetic component to levels of DNA methylation at smoking-associated DMPs; overall there were significantly higher contributions of additive genetic influences (mean A = 37.7% (SD = 22.2%); Mann-Whitney P = 3.20x10^-12^) as well as shared environmental influences (mean C = 23.5% (SD = 16.0%); Mann-Whitney P = 0.00419) across smoking-associated DMPs compared to all “variable” DNA methylation sites, with a significantly smaller contribution of unique environmental influences (mean E = 38.9% (SD = 17.4%); Mann-Whitney P = 5.47x10^-16^) (**Fig 6**). We next attempted to control for the fact that smoking behavior (and therefore the “exposure” itself) is a heritable trait [45, 46]; by only considering 18-year-old twin pairs where both members have never smoked it can be assumed that the influence of tobacco exposure on DNA methylation is negligible and any observed heritability at these sites cannot result from smoking. For 95 of 97 smoking-associated DMPs, the correlation of DNA methylation in MZ concordant non-smokers (N = 315 twin-pairs) was greater than in DZ concordant non-smokers (N = 187 twin pairs) (**Fig 6**), representing a significant enrichment (P = 6.00x10^-26^). **S25 Fig** highlights two DMPs at which DNA methylation was strongly associated with smoking status (cg05575921: P = 1.73x10^-80^; cg26703534: P = 1.39x10^-90^) but also was notably more correlated in MZ twin pairs (cg05575921: r = 0.845; cg26703534 r = 0.658) than DZ twin pairs (cg05575921: r = 0.579; cg26703534: r = 0.444). These data are important because they provide evidence that smoking effects are not necessarily independent of smokers’ genetic background, and that it is important to control for genetic background when testing for effects of tobacco on health. We also explored the genetic and environmental contributions to variation in DNA methylation at DMPs robustly associated with BMI[47], again observing that these had significantly higher additive genetic influences (mean A = 31.4% (SD = 19.4%); Mann-Whitney P = 1.83x10^-11^) and shared environmental influences (mean C = 23.4% (SD = 15.4%)); Mann-Whitney P = 2.16x10^-13^) compared to all “variable” DNA methylation sites (**S26 Fig; S5 Table**). These data highlight how DNA methylation at sites robustly associated with extrinsic factors can also be under strong genetic control, highlighting the need to control for genetic background in future EWAS analyses of exposure-associated DNA methylation differences.

## Discussion

We quantified genome-wide patterns of DNA methylation in whole blood in 18-year-old young adults using samples collected from a large representative birth cohort of MZ and same-sex DZ twin pairs. We show that site-specific levels of DNA methylation are more strongly correlated between MZ twins than DZ twins, especially at sites with variable and intermediate levels of DNA methylation. Using structural equation models, we calculated the proportion of variance in DNA methylation explained by additive genetic effects, shared environmental effects and unshared (or unique) environmental effects, finding that, on average, the largest contribution to variation in DNA methylation can be attributed to unique environmental influences. Although the average contribution of additive genetic influences on DNA methylation was found to be relatively lower, it is variable and notably elevated at DNAm sites that are highly variable and have intermediate levels of DNAm. Interestingly, sites at which variable DNA methylation is strongly influenced by additive genetic factors are significantly enriched for blood mQTL effects, and also for sites at which inter-individual variation is correlated across tissues. Finally, we show that DNA methylation at sites robustly associated with exposures such as tobacco smoking and BMI is, in fact, also influenced by additive genetic effects, implying that environmental epigenetics research should routinely control for genetic background in future analyses. Estimates of the contribution of genetic and environmental influences to DNA methylation at all sites profiled in this study are available as a resource for the research community (http://www.epigenomicslab.com/online-data-resources).

Unlike previous studies that have used twins to explore the genetic and environmental architecture of DNA methylation [19, 21, 23], we focused solely on same-sex twins who were all the same chronological age, enabling us to negate the effects of age and DZ twin sex-discordance on variable DNA methylation. Despite these strengths, however, our study has a number of important limitations that should be considered. First, because our analyses focused solely on a cross-section of young adults we cannot say anything about how genetic and environmental influences on DNA methylation change over time. Of note, our average estimate of additive genetic influences on DNA methylation is slightly below that observed in previous studies of older and more variably-aged twin-pairs [19, 23]. Second, our study cohort comprised individuals of European descent, like most other studies into the causes of variable DNA methylation. We know, however, that there are important racial and socioeconomic inequalities in pathogenic exposures and it is crucial that future work explores the contribution of genetic and environmental contributions to epigenetic variation in non-Caucasian populations. Third, although the Illumina 450K array quantifies DNA methylation at sites annotated to the majority of genes, the actual proportion of sites across the genome interrogated by this technology is relatively low, with a predominant focus on CpG-rich promoter regions. It will be important for future studies to explore factors influencing levels of DNA methylation across regions not well-covered by the Illumina 450K array, especially given our finding that genetic and environmental influences on DNA methylation are not evenly distributed across genic regions. Of note, most of the content (> 90%) of the Illumina 450K array is present on the new Illumina EPIC array [48] and the results presented here are therefore applicable to future studies using this technology. Fourth, our study only assessed a single tissue–whole blood–which itself is comprised of a heterogeneous mix of different cell-types. Although blood cell-type proportions can be accurately derived from whole blood DNA methylation data, it is likely that the contribution of genetic and environmental factors to methylomic variation differs across different cell-types. Future work should extend these analyses to quantify DNA methylation in purified blood cell-types and cell isolated from other tissues from MZ and DZ twins to explore the extent to which our findings are generalizable across tissues and cell-types. Of note, DNA methylation sites at which inter-individual variation is correlated across tissues were characterized by higher heritability, suggesting that genetic effects on DNA methylation may be relatively conserved across tissues and cell types.

Although the largest contributor to inter-individual variation in DNA methylation across all tested sites was found to be non-shared environmental factors, which also captures measurement error, our findings highlight the importance of genetic influences on DNA methylation. Genetic influences appear to be especially important in mediating levels of DNA methylation at highly variable DNA methylation sites and those that are characterized by high levels of covariation across tissues suggesting that concerns relating to tissue-specific effects may be less relevant for genetic studies of DNA methylation. As expected, sites at which variable DNA methylation is strongly influenced by additive genetic factors are significantly enriched for known mQTL effects. Our results could be potentially used to improve the power of mQTL studies by providing a refined list of ‘heritable’ DNA methylation sites, thereby reducing the multiple testing burden and sample sizes needed to identify significant mQTL associations. The mean estimate of shared environmental effects on DNAm across the genome was higher than previously reported [21] and comparable to the magnitude of influence of additive genetic factors. Given the young and comparable ages of the participants in the E-Risk cohort (all ~ 18 years old) it is plausible that a higher proportion of environmental influences are shared between the twins compared to the variably-aged and older twin pairs profiled in other studies.

To conclude, we have characterized the genetic and environmental architecture of methylomic variation in a large sample of young adult MZ and DZ twins. We show that both heritable and non-genetic factors influence DNA methylation in a site-specific manner, with the contribution of genetic variation being highest at the most variable DNA methylation sites. Social-science and health researchers in search of evidence for environmental effects on the genome should not assume that “epigenetic” equates to “environmental”. Importantly, DNA methylation at sites robustly associated with extrinsic factors such as smoking and BMI can also be under strong genetic control. Our online database provides estimates of the extent to which variable DNA methylation across all sites profiled in this study are under genetic influence. Although this resource is limited by some of the features of this study–i.e. it focuses on individuals of European descent, a single age-group, and sites on the Illumina 450K array–it provides a useful framework for interpreting the results of epigenetic epidemiological studies undertaken in whole blood.

## Materials and methods

### Ethics statement

The study was approved by the NRES Committee London—Camberwell St Giles Ethics Committee, and The Joint South London and Maudsley and the Institute of Psychiatry Research Ethics Committee approved each phase of the E-Risk study (reference number: 1997/122). Parents gave written informed consent and twins gave oral assent between 5–12 years and then written informed consent at age 18.

### Samples

Participants were members of the Environmental Risk (E-Risk) Longitudinal Twin Study, which tracks the development of a 1994–95 birth cohort of 2,232 British children[22]. Briefly, the E-Risk sample was constructed in 1999–2000, when 1,116 families (93% of those eligible) with same-sex 5-year-old twins participated in home-visit assessments. This sample comprised 56% monozygotic (MZ) and 44% dizygotic (DZ) twin pairs; sex was evenly distributed within zygosity (49% male). The study sample represents the full range of socioeconomic conditions in Great Britain, as reflected in the families’ distribution on a neighborhood-level socioeconomic index (ACORN [A Classification of Residential Neighbourhoods], developed by CACI Inc. for commercial use)[49]: 25.6% of E-Risk families live in “wealthy achiever” neighborhoods compared to 25.3% nationwide; 5.3% vs. 11.6% live in “urban prosperity” neighborhoods; 29.6% vs. 26.9% in “comfortably off” neighborhoods; 13.4% vs. 13.9% in “moderate means” neighborhoods; and 26.1% vs. 20.7% in “hard-pressed” neighborhoods. E-Risk underrepresents “urban prosperity” neighborhoods because such households are often childless.

Home visits were conducted when participants were aged 5, 7, 10, 12 and most recently, 18 years (93% participation). Our epigenetic study used DNA from a single tissue: whole blood. At age 18, whole blood was collected in 10mL K2EDTA tubes from 1,700 participants and DNA extracted from the buffy coat. (Study members who did not provide blood provided buccal swabs, but these were not included in our methylation analysis to avoid tissue-source confounds). There were no differences between participants who did versus did not participate and who did versus did not provide blood in terms of their socioeconomic background, IQ, mental health, or victimization experiences [50].

### Genome-wide quantification of DNA methylation

We assayed 1669 blood samples (out of 1700); 31 samples were not useable (e.g., due to low DNA concentration). ~500ng of DNA from each sample (diluted to a standard concentration of 25ng/μL) was treated with sodium bisulfite using the EZ-96 DNA Methylation kit (Zymo Research, CA, USA). DNA methylation was quantified using the Illumina Infinium HumanMethylation450 BeadChip (“Illumina 450K array”) run on an Illumina iScan System (Illumina, CA, USA). Twin pairs were randomly assigned to bisulfite-conversion plates and Illumina 450K arrays, with siblings processed in adjacent positions to minimize batch effects. Data were imported using the methylumIDAT function in methylumi[51] and subjected to quality control analyses, checking for sex mismatches, genotype data that did not concur with those typed on Illumina OmniExpress24v1.2 arrays, and excluding low intensity samples (details in [50]). In total, samples from 1658 participants passed our QC pipeline. Data were processed with the *pfilter* function from the *wateRmelon* package[52] excluding 0 samples with >1% of sites with a detection p value >0.05, 567 sites with beadcount <3 in 5% of samples and 1448 probes with >1% of samples with detection p value >0.05. The data were normalized with the *dasen* function from the wateRmelon package[52]. This article reports about 732 complete twin pairs (426 MZ and 306 same-sex DZ). Prior to any analyses, probes with common (>5% MAF) SNPs within 10 bp of the single base extension and probes with sequences previously identified as potentially hybridizing to multiple genomic loci were excluded[53, 54], resulting in a final dataset of 430,802 probes. Zygosity of twin pairs in the E-Risk cohort was confirmed in two ways. First, signal intensities at the 65 SNP probes on the Illumina 450K array were used to confirm that MZ twins were genetically identical. Second, SNP array genotype data for these samples was used to confirm that MZ twins shared 100% of their genetic variation (PI_HAT = 1) and DZ twins shared ~ 50% of their genetic variation (PI_HAT ~ 0.5). The results from these two stages were then cross-validated for final confirmation.

### Structural equation modelling to estimate the contribution heritable and environmental influences on DNA methylation

Biometrical modelling was applied to every probe passing QC on the Illumina 450K array. Specifically, an ACE model was fitted to calculate the proportion of variance in DNA methylation explained by additive genetic (A), shared environmental (C) and unshared or unique environmental (E) factors, the latter which also includes measurement error. The assumptions behind this model are that additive genetic factors are perfectly correlated between MZ twins (i.e. genetic correlation = 1) but are only 50% correlated between DZ twins (i.e. genetic correlation = 0.5) and that shared non-heritable influences are equally similar between MZ and DZ twin pairs. The model was fitted using structural equation modelling implemented with functions from the OpenMx R package [55, 56]. For DNA methylation sites located on the autosomes this model was fitted using all twin pairs; for sites located on the X chromosome, the analysis was performed separately for males and females. Given the sparse coverage on the Y chromosome, Y-linked sites were dropped from analysis. The same model was used to calculate A,C and E estimates for the predicted age and cell composition variables generated with the Epigenetic Clock software[32].

### Probe annotation of sites in the 450K array

The location of DNA methylation sites within genic features (5’UTR, 3’UTR, 1^st^ Exon, gene body, within 200 or 1500bp of the transcription start site [TSS] and CpG island categories [CpG Island, shelf, shore]) were taken from the annotation files provided by Illumina (ftp://ussd-ftp.illumina.com/downloads/ProductFiles/HumanMethylation450/HumanMethylation450_15017482_v1-2.csv).

### DNA methylation quantitative trait loci

DNA methylation quantitative trait loci (mQTL) were taken from a previously published study based on whole blood profiles from 639 adult samples [29]. After testing all DNA methylation sites against all genetic variants, 8,960,441 mQTL were identified using a p value threshold of 1x10^-10^. From this set of mQTL, 98,239/389,246 (25.2%) of DNA methylation sites overlapping with the heritability analysis had an mQTL.

### DNA methylation sites associated with tobacco smoking

To identify DNA methylation sites associated with tobacco smoking, a linear regression model was fitted across the extended E-Risk sample including singletons (n = 1,658). Current smokers (N = 392) were compared against former (N = 42) and never smokers (N = 1,223) whilst controlling for sex, batch, and 7 estimated variables relating to cellular heterogeneity generated with either the Houseman algorithm [33, 34] or Horvath Epigenetic clock [32]. To control for the fact that many members of the sample are related robust standard errors were calculated with the R packages *plm* [57] and sandwich [58] and used to generate p-values. 97 DNA methylation sites were associated with current smoking status at an experiment-wide p-value threshold of 1x10^-7^. It should be noted that the exact number of genome-wide significant associations for tobacco smoking differs slightly from that reported in [50] due to differences in methods used to account for related samples and due to filtering DNA methylation sites based on their variability.

### DNA methylation sites associated with BMI

DNA methylation sites associated with BMI were identified from the supplementary material published as part of the EWAS performed by Wahl et al [47]. Taking their 187 replicated, sentinel associations, 176 of these were present in our set of variable DNA methylation sites and therefore were included for comparison with our estimates of heritability.

## Supporting information

S1 Table The extent to which DNA methylation levels at sites annotated to specific genic features and CpG island features are enriched for the influence of additive genetic or environmental factors. (PDF)

S2 Table Sites at which DNA methylation is strongly influenced by additive genetic effects are often associated with mQTL variation. (XLSX)

S3 Table Estimated contribution of additive genetic and environmental influences to estimated age and blood cell proportion estimates derived from DNA methylation data. (PDF)

S4 Table Estimates of additive genetic and environmental influences on levels of DNA methylation at the 97 differentially methylated positions (P < 1x10^-7^) associated with tobacco smoking. (PDF)

S5 Table Estimates of additive genetic and environmental effects on levels of DNA methylation at 176 differentially methylated positions associated with BMI. (PDF)

S1 Fig Genome-wide patterns of DNA methylation are highly correlated between siblings, with significantly higher average similarity in monozygotic (MZ) twin-pairs than dizygotic (DZ) twin-pairs. Shown are violin plots for the average correlations of DNA methylation within each sibling pair (stratified by relatedness) averaged across **A)** all autosomal DNA methylation sites (n = 420,857), **B)** autosomal sites characterized by “variable” DNA methylation (n = 214,991), **C)** autosomal sites characterized by “non-variable” DNA methylation (n = 205,866), **D)** autosomal sites with intermediate levels of DNA methylation (n = 131,728), and **E)** autosomal sites characterized as being either hypo- or hyper-methylated (n = 289,129). P-values are from a t-test comparing average correlations observed in MZ twins to those observed in DZ twins. Also shown are comparisons between random pairs of unrelated individuals selected from the E-Risk cohort.
(PDF)

S2 Fig There is considerable overlap between the set of autosomal DNA methylation sites defined as being ‘variable’ and having intermediate levels of DNA methylation. (TIFF)

S3 Fig **The proportion of variance in DNA methylation explained by additive genetic effects (A), shared environmental effects (C) and unshared (or unique) environmental effects (E) across autosomal sites after adjusting for cellular composition.** Panels **a-c** show density distributions for estimates of A, C, and E across all 420,857 autosomal DNA methylation sites. At the majority of autosomal sites, environmental factors contribute more to the observed variance in DNA methylation than additive genetic factors. We observe significantly higher average heritability estimates for DNA methylation across the subset of DNA methylation sites defined as “variable” (**d-f**) (mean A = 29.3% (SD = 25.0%); Mann Whitney P < 2.2x10^-16^) and (**g-i**) sites with intermediate levels of DNA methylation (mean A = 24.3% (SD = 24.2%); Mann Whitney P < 2.2x10^-16^).
(PDF)

S4 Fig The contribution of genetic and environmental influences on DNA methylation is not strongly influenced by blood cell heterogeneity. Scatterplots of additive genetic effects (A), shared environmental effects (C) and non-shared (or unique) environmental effects (E) for all autosomal DNA methylation sites (n = 420,857), comparing DNA methylation data unadjusted for cellular composition (x-axis) and DNA methylation data adjusted for cellular composition variables (y-axis). Each point represents a DNA methylation site and the colour of the point indicates the density of points at that location (gray–low to yellow–high).
(PDF)

S5 Fig The contribution of genetic and environmental influences on DNA methylation at autosomal sites differs as a function of the variability in DNA methylation level. Shown are estimates of additive genetic effects (A), shared environmental effects (C) and non-shared (or unique) environmental effects (E) plotted as a function of the variability in DNA methylation measured by **A**) the standard deviation (SD) and **B**) the range of the middle 80% of the distribution of DNA methylation levels. In panel **B**, the dashed vertical line indicates the cut-off (5%) used to define DNA methylation sites as being “variable” in this study.
(PDF)

S6 Fig The contribution of genetic and environmental influences on DNA methylation at autosomal sites are not evenly distributed across genic regions. Shown is a line graph depicting the extent to which variation in DNA methylation is influenced by genetic and environmental factors across a canonical gene region. Genetic influences on DNA methylation are highest immediately upstream of the transcription start-site (TSS), and in the region spanning 5 kilobases downstream of the gene coding sequence (red line, panel **A**). Sites located around the TSS are enriched for shared environmental effects (blue line, panel **A**) and show reduced non-shared environmental effects (green line, panel **B**).
(PDF)

S7 Fig The contribution of genetic and environmental influences on DNA methylation at autosomal sites annotated to specific genic features. Shown is a density plot of estimates of **A)** additive genetic, **B)** shared environmental, and **C**) non-shared environmental influences on DNA methylation at autosomal sites stratified by gene feature annotation.
(PDF)

S8 Fig The contribution of genetic and environmental influences on DNA methylation at autosomal sites annotated to specific CpG island features. Shown is a density plot of estimates of **A)** additive genetic, **B)** shared environmental, and **C**) non-shared environmental influences on DNA methylation at sites stratified by CpG island feature annotation.
(PDF)

S9 Fig The influence of genetic and environmental factors on DNAm varies across regulatory features and chromatin states. Violin plots showing the proportion of variance explained by additive genetic factors (A; red), common environmental factors (C; green), and unique environmental factors (E; blue) where DNA methylation sites are stratified by their location in regulatory annotation states as defined by ChromHMM [59] using ENCODE experimental data from the GM12878 cell line.
(PDF)

S10 Fig Sites with intermediate levels of DNAm are associated with larger DNA methylation trait quantitative trait loci (mQTL) effects. Line graph of the moving mean mQTL effect on DNA methylation (measured as the % DNA methylation change per allele; y-axis) as a function of mean DNA methylation (%; x-axis). The gray area indicates the 95% interquantile range for the moving average.
(PDF)

S11 Fig Example of a site at which DNA methylation is highly heritable (A = 96.9%) and associated with genotype at a DNA methylation trait quantitative trait loci (mQTL). Panel **A**) shows a boxplot of the association between DNA methylation at cg02573566 and genotype at rs11548104 (*P* = 5.95x10^-179^). Panel **B**) shows the correlation in DNA methylation at cg02573566 between MZ twins (r = 0.916) and panel **C)** shows the correlation in DNA methylation at cg02573566 between DZ twins (r = 0.487).
(PDF)

S12 Fig Sites at which DNA methylation is more strongly influenced by genetic factors are more likely to be associated with genotype at a mQTL. Shown is a line graph of the percentage of DNA methylation sites significantly associated with an mQTL variant in our whole blood dataset[29] (y-axis) as a function of increasing cut-offs for estimates of additive genetic (black line), shared environmental (red line) and non-shared environmental (blue line) effects on DNA methylation (x-axis).
(PDF)

S13 Fig There is a strong correlation between the extent to which inter-individual variation in DNA methylation co-varies across tissues with the influence of additive genetic variation on DNA methylation. Scatterplot of the amount of variance in DNA methylation explained by additive genetic effects (y-axis) against the level of blood-brain covariation in DNA methylation (x-axis) using data from Hannon et al[30] for all sites on the Illumina 450K array. Shown is data for covariation between whole blood and **A**) prefrontal cortex, **B**) entorhinal cortex **C**) superior temporal gyrus and **D**) cerebellum. Color indicates the density of points ranging from yellow (high) to gray (low). PFC = prefrontal cortex, EC = entorhinal cortex, STG = superior temporal gyrus, CER = cerebellum.
(PDF)

S14 Fig Distribution of DNA methylation levels across sites on the X chromosome. A) Shown is a density plot of DNA methylation across sites on the X chromosome stratified by sex. B) Shown is a scatterplot comparing mean DNA methylation at sites across the X-chromosome in females (x-axis) and males (y-axis).
(PDF)

S15 Fig Sex differences for the proportion of variance in DNA methylation explained by additive genetic and environmental influences for sites on the X chromosome. Shown are density plots of estimates of additive genetic effects (A), shared environmental effects (C) and non-shared (or unique) environmental effects (E) stratified by sex (red = females, green = males).
(PDF)

S16 Fig The contribution of genetic and environmental influences on DNA methylation at sites on the X-chromosome differs as a function of mean DNA methylation with notable differences between males and females. Shown for **A)** males and **B)** females are estimates of additive genetic effects (A), shared environmental effects (C) and non-shared (or unique) environmental effects (E) plotted as a function of average DNA methylation level.
(PDF)

S17 Fig The contribution of genetic and environmental influences on DNA methylation at sites on the X-chromosome differs as a function of the variability in DNA methylation level, with notable differences between males and females. Shown are estimates of additive genetic effects (A), shared environmental effects (C) and unshared (or unique) environmental effects (E) against probe variability. Panels **A** and **B** show how genetic and environmental influences differ as a function of the standard deviation (SD) in DNA methylation in males and females, respectively. Panels **C** and **D** show how genetic and environmental influences differ as a function of the middle 80% of the distribution of DNA methylation levels in males and females, respectively. The dashed vertical line indicates the cut-off of 5% used to define probes as being “variable”.
(PDF)

S18 Fig The contribution of genetic and environmental influences on DNA methylation at sites on the X-chromosome is modestly correlated between males and females. Shown are scatterplots of the **A**) additive genetic, **B**) shared environmental, and **C**) non-shared environmental contribution to DNA methylation for sites on the X chromosome in female (x-axis) and male (y-axis) twin pairs.
(PDF)

S19 Fig An example of a site (cg00195237) on the X chromosome at which DNA methylation is strongly influenced by additive genetic factors in females (A = 56.8%) but not males (A = 6.70%). The scatterplots show DNA methylation values in **A**) female MZ, **B**) female DZ, **D**) male MZ, and **E**) male DZ twin pairs. Each point represents an individual twin-pair. At this site, the twin correlation of DNA methylation is notably higher in female MZ twins (r = 0.569) compared to female DZ twins (r = 0.15), whereas the correlations for male MZ twins (r = 0.0686) and male DZ twins (r = 0.0289) are similar. Results from structural equation modelling are presented as stacked bar-plots for **C**) female and **F**) male twin-pairs respectively, highlighting higher genetic effects on DNA methylation at this site in females than males.
(PDF)

S20 Fig An example of a site (cg19782749) on the X chromosome at which DNA methylation is strongly influenced by additive genetic factors in males (A = 58.9%) but not females (A = 3.76%). The scatterplots show DNA methylation values in **A**) female MZ, **B**) female DZ, **D**) male MZ, and **E**) male DZ twin pairs. Each point represents an individual twin-pair. At this site, the correlation of DNA methylation is notably higher in male MZ twins (r = 0.777) compared to female DZ twins (r = 0.536), whereas the correlations for male MZ twins (r = 0.405) and male DZ twins (r = 0.378) are similar. Results from structural equation modelling are presented as stacked bar-plots for **C**) female and **F**) male twin-pairs respectively, highlighting higher heritability in females than males.
(TIF)

S21 Fig The proportion of variance in DNA methylation explained by additive genetic and environmental influences for sites on the X chromosome. Shown are density plots of estimates of additive genetic effects (A), shared environmental effects (C) and non-shared (or unique) environmental effects (E) stratified by sex and within females stratified by sites located in the transcription start site or 5’UTR of genes that escape XCI (red = females, blue = females sites that escape X chromosome inactivation, green = males).
(PDF)

S22 Fig Twin-pair correlations for estimates of DNA methylation age and blood cell composition derived from DNA methylation data. Shown are co-twin correlations for **A)** DNA methylation age, **B)** estimated plasma blast abundance, **C)** estimated CD8+CD28-CD45RA- T cell abundance, **D)** estimated naïve CD8 T cell abundance, **E)** estimated naive CD4 T cell abundance (all derived using the online Epigenetic Clock software[32]), **F)** estimated CD8 T cell proportion, **G)** estimated CD4 T cell proportion, **H)** estimated natural killer cell proportion, **I)** estimated B cell proportion, **J)** estimated monocyte proportion, and **K)** estimated granulocyte proportion (all derived using the Houseman algorithm[33, 34]). Panels on the left show correlations for monozygotic (MZ) twin pairs and panels on the right show correlations for dizygotic (DZ) twin pairs.
(PDF)

S23 Fig The contribution of additive genetic and environmental influences to age and blood cell-count estimates derived from the DNA methylation data. AAR = age acceleration residual derived from the DNA methylation age clock.
(PDF)

S24 Fig Effect sizes at DNA methylation sites associated with tobacco smoking in the E-risk cohort overlap with those previously identified in adult cohorts. **A)** The mean difference between current smokers and never smokers from the E-risk cohort (x-axis) against a similar study in adults taken from Joehanes et al[44] (y-axis). **B)** Shown is the correlation of the signed log10 P-values from a comparison between current smokers and never smokers from the E-risk cohort (x-axis) against a similar study in adults taken from Joehanes et al[44] (y-axis).
(PDF)

S25 Fig Examples of DNA methylation sites associated with smoking that are influenced by both additive genetic and environmental factors. Scatterplot of DNA methylation values at cg05575921 for **A**) monozygotic (MZ) twin pairs and **B)** dizygotic (DZ) twin pairs, and cg26703534 for **C**) MZ twin pairs and **D)** DZ twin pairs. Colors depict the concordance for current smoking status in each twin-pair.
(TIF)

S26 Fig DNA methylation at sites associated with body mass index (BMI) is influenced by additive genetic factors. Density plots for estimates of **A**) additive genetic effects (A), **B**) shared environmental effects (C), and **C**) non-shared environmental effects (E) at 176 differentially methylated positions (DMPs) recently associated with BMI (green)[47].
(PDF)
