# Supplementary material for: Characterizing genetic and environmental influences on variable DNA methylation using monozygotic and dizygotic twins
Source: PLoS Genet. 2018 Aug 9;14(8):e1007544. doi: 10.1371/journal.pgen.1007544 (PMC6084815; doi:10.1371/journal.pgen.1007544)
Supplement: S1 Table — (PDF) [file pgen.1007544.s001.pdf]

	Additive genetic (A)		Common environment (C)		Non-shared environment (E)	
	**Mean**	**P value**	**Mean**	**P value**	**Mean**	**P value**
Genic features						
**Body**	21.6%	0	15.8%	$2.16x10^{-23}$	62.6%	0
**TSS**	23.7%	0	18.5%	$4.26x10^{-51}$	57.8%	0
**3'UTR**	18.2%	$2.41x10^{-26}$	15.8%	0.314822	66.0%	$4.43x10^{-22}$
**5'UTR**	21.7%	0	19.0%	$8.91x10^{-82}$	59.3%	0
**1stExon**	24.9%	0	21.6%	$2.74x10^{-64}$	53.6%	0
**Intergenic**	25.4%	0	13.7%	0	60.9%	0
CpG island features						
**CpG Island**	28.5%	0	21.0%	$1.14x10^{-150}$	50.5%	0
**Shelf**	18.7%	$5.94x10^{-135}$	15.2%	$4.96x10^{-19}$	66.1%	$2.92x10^{-54}$
**Shore**	25.8%	0	15.8%	$1.63x10^{-52}$	58.4%	0
**Sea**	20.7%	0	14.3%	0	65.0%	0
