# Supplementary material for: Characterizing genetic and environmental influences on variable DNA methylation using monozygotic and dizygotic twins
Source: PLoS Genet. 2018 Aug 9;14(8):e1007544. doi: 10.1371/journal.pgen.1007544 (PMC6084815; doi:10.1371/journal.pgen.1007544)
Supplement: S3 Table — (PDF) [file pgen.1007544.s003.pdf]

Variable	A	C	E
**Age Acceleration***	0.367146461	0.427927378	0.204926161
**Plasma Blast***	0.377622832	0.128915739	0.493461428
**CD8pCD28nCD45RAn T cells***	0.469348045	0.038002035	0.492649919
**naive CD8 T cells***	0.421183455	0.233812316	0.345004229
**naive CD4 T cells***	0.335581433	0.311508146	0.352910421
**CD8 T cells**$^{+}$	5.28E-14	0.420515113	0.579484887
**CD4 T cells**$^{+}$	0.273315615	0.284137745	0.44254664
**Natural killer**$^{+}$	0.399613256	0.202252486	0.398134257
**B cells**$^{+}$	0.202983521	0.521349375	0.275667104
**Monocytes**$^{+}$	0.269308024	0.242800259	0.487891717
**Granulocytes**$^{+}$	6.12E-15	0.408301181	0.591698819
