# Supplementary material for: Characterizing genetic and environmental influences on variable DNA methylation using monozygotic and dizygotic twins
Source: PLoS Genet. 2018 Aug 9;14(8):e1007544. doi: 10.1371/journal.pgen.1007544 (PMC6084815; doi:10.1371/journal.pgen.1007544)
Supplement: S4 Table — (PDF) [file pgen.1007544.s004.pdf]

Probe ID	EWAS of smoking (current vs never)		A	C	E	Chromosome	Position
	Mean DNA methylation difference	P value					
cg00232092	-1.08	3.77E-09	0.0%	34.1%	65.9%	7	5518887
cg00308065	0.93	5.40E-08	0.0%	24.1%	75.9%	12	124422135
cg00501876	-1.23	1.62E-16	71.6%	26.9%	1.5%	3	39193251
cg01062937	0.74	4.96E-08	18.8%	42.9%	38.3%	16	88537260
cg01899089	-1.76	6.84E-16	36.4%	25.1%	38.5%	5	369969
cg01940273	-3.64	1.61E-30	57.9%	15.5%	26.6%	2	233284934
cg02228160	1.25	7.50E-09	44.7%	21.3%	34.0%	5	143192067
cg02451831	-1.82	3.53E-17	26.9%	17.2%	55.9%	7	26578098
cg02532700	-1.42	6.25E-08	41.6%	27.4%	31.0%	22	37257404
cg03450842	-1.39	2.74E-15	79.7%	19.5%	0.8%	10	80834947
cg03636183	-3.62	9.99E-33	43.3%	27.3%	29.4%	19	17000585
cg03991871	-2.83	5.05E-17	73.1%	0.0%	26.9%	5	368447
cg04039799	-1.17	2.39E-08	12.7%	34.6%	52.6%	11	19745484
cg04180046	2.68	5.07E-12	41.3%	28.8%	29.9%	7	45002736
cg04387347	2.34	9.90E-12	48.8%	10.7%	40.5%	16	88537187
cg04460609	-1.66	6.97E-10	40.0%	28.0%	32.0%	4	16532808
cg04551776	-1.24	3.84E-09	11.1%	31.4%	57.5%	5	393366
cg04640972	1.26	2.60E-09	18.3%	25.7%	56.1%	10	8373522
cg04885881	-1.96	3.72E-15	52.7%	9.7%	37.6%	1	11123118
cg05221370	-1.20	2.98E-09	18.2%	12.9%	68.9%	7	110738836
cg05460226	-2.16	8.75E-14	28.6%	17.5%	53.8%	17	8804279
cg05508862	1.00	4.95E-09	57.7%	6.6%	35.7%	17	18885437
cg05575921	-9.70	1.73E-80	59.8%	25.6%	14.6%	5	373378
cg05824218	0.85	7.18E-08	19.3%	20.5%	60.2%	17	38499096
cg05951221	-3.64	1.89E-27	74.6%	12.7%	12.7%	2	233284402
cg06126421	-3.57	6.06E-45	48.4%	14.1%	37.5%	6	30720080
cg07339236	-2.03	5.69E-30	21.4%	47.2%	31.4%	20	50312490
cg07826859	-1.29	1.34E-18	16.7%	23.0%	60.3%	7	45020086
cg07986378	-2.37	2.67E-19	23.4%	19.7%	57.0%	12	11898284
cg08709672	-1.80	1.66E-17	61.2%	22.1%	16.7%	1	206224334
cg08763102	-0.89	1.98E-09	72.7%	25.6%	1.7%	4	3079751
cg08972170	2.09	7.66E-12	61.8%	0.0%	38.2%	7	30185776

cg09022230	-2.02	1.09E-16	38.0%	22.7%	39.4%	7	5457225
cg09560590	1.16	1.51E-09	51.9%	19.2%	28.8%	5	143191663
cg09651136	1.05	1.27E-09	14.6%	20.2%	65.2%	15	72525012
cg09935388	-3.98	1.18E-18	53.4%	21.8%	24.8%	1	92947588
cg10062919	-0.74	5.18E-08	18.7%	35.8%	45.4%	17	38503802
cg10420527	-1.06	4.89E-13	6.9%	44.2%	48.9%	11	68138505
cg10750182	-1.03	6.66E-15	14.8%	24.2%	61.0%	10	73497514
cg10965178	-1.15	3.47E-09	30.0%	30.2%	39.8%	1	43766752
cg11071448	-1.84	1.29E-13	0.0%	48.3%	51.7%	1	202584465
cg11730703	1.02	5.76E-09	48.1%	12.9%	39.0%	14	105167607
cg11824827	1.66	7.97E-12	30.2%	19.5%	50.3%	16	31075547
cg12803068	6.20	2.36E-20	63.9%	25.4%	10.7%	7	45002919
cg12806681	-1.31	8.24E-09	43.5%	7.3%	49.2%	5	368394
cg13193840	-0.85	2.81E-08	16.2%	18.3%	65.5%	2	233285289
cg13578465	1.66	1.06E-09	63.9%	11.9%	24.2%	14	105167457
cg14179389	-2.16	4.92E-09	41.5%	26.9%	31.6%	1	92947961
cg14624207	-0.96	5.85E-08	6.1%	31.7%	62.2%	11	68142198
cg14712058	-1.21	2.00E-10	31.2%	24.8%	44.0%	19	16988083
cg14753356	-2.60	2.09E-29	16.0%	32.6%	51.4%	6	30720108
cg14817490	-2.80	1.77E-24	35.4%	36.1%	28.5%	5	392920
cg15159987	-1.51	1.52E-18	10.4%	30.2%	59.5%	19	17003890
cg15542713	3.26	7.52E-12	76.7%	0.0%	23.3%	1	42385581
cg15806304	1.13	2.15E-09	21.7%	39.0%	39.3%	10	119000083
cg16145216	2.34	3.10E-12	83.7%	0.0%	16.3%	1	42385662
cg16624521	1.23	1.88E-09	63.5%	11.6%	24.9%	5	172064552
cg16694480	1.38	8.15E-09	40.0%	18.2%	41.8%	1	19732786
cg17907003	-1.45	5.09E-12	16.0%	34.2%	49.8%	1	117533414
cg18804920	1.33	3.32E-10	6.2%	17.1%	76.7%	7	146572638
cg19089201	2.60	2.97E-12	49.4%	34.0%	16.5%	7	45002287
cg19406367	1.26	2.33E-09	40.2%	18.6%	41.2%	1	66999929
cg19572487	-1.45	5.30E-10	39.1%	60.5%	0.4%	17	38476024
cg20059012	-2.80	8.50E-10	53.7%	0.6%	45.8%	12	53613154
cg20059928	-1.97	9.66E-08	82.5%	0.0%	17.5%	15	40361485
cg20244340	-1.43	1.17E-08	9.6%	88.6%	1.7%	20	19193989

cg20295214	-1.40	1.22E-15	28.5%	6.2%	65.4%	1	206226794
cg20438687	-1.13	2.21E-08	43.5%	12.4%	44.1%	17	4761644
cg21161138	-3.94	2.30E-48	36.9%	7.6%	55.4%	5	399360
cg21304158	-0.64	7.47E-08	32.7%	26.2%	41.1%	2	241937034
cg21322436	-1.21	3.27E-13	0.2%	46.6%	53.2%	7	145812842
cg21446172	-1.08	3.53E-08	55.0%	0.0%	45.0%	1	223745234
cg21611682	-1.55	2.62E-23	54.0%	45.7%	0.3%	11	68138269
cg21733098	-3.32	4.19E-13	62.1%	22.5%	15.3%	12	127931219
cg21747070	-1.13	1.05E-08	47.2%	0.0%	52.8%	5	957535
cg22132788	4.38	1.63E-26	68.4%	19.1%	12.5%	7	45002486
cg22539182	1.45	3.41E-10	52.1%	14.8%	33.2%	10	850393
cg23126342	2.32	8.95E-09	56.5%	21.7%	21.9%	13	67801125
cg23161492	-1.35	7.17E-08	61.1%	9.9%	29.0%	15	90357202
cg23551720	-0.70	5.71E-08	10.8%	18.9%	70.3%	17	46633726
cg23842572	1.05	9.76E-09	6.2%	36.3%	57.6%	17	17030253
cg23916896	-2.60	4.22E-17	33.1%	23.3%	43.6%	5	368804
cg23973524	1.98	1.74E-10	79.2%	0.0%	20.8%	19	18873222
cg24049493	2.27	7.95E-09	81.3%	0.0%	18.7%	1	42385941
cg24090911	-1.85	2.75E-21	33.4%	0.0%	66.6%	5	400732
cg24556382	-2.11	6.62E-11	60.8%	0.0%	39.2%	4	174173455
cg24688690	-0.86	6.21E-10	29.9%	13.7%	56.4%	5	345850
cg24838345	-1.66	4.34E-10	47.6%	0.0%	52.4%	8	125737353
cg25189904	-3.77	7.91E-25	39.7%	24.6%	35.8%	1	68299493
cg25648203	-2.85	2.85E-33	29.6%	31.8%	38.6%	5	395444
cg25949550	-0.80	6.82E-10	34.2%	26.4%	39.4%	7	145814306
cg26361535	-1.57	9.33E-09	59.3%	9.8%	30.9%	8	144576604
cg26529655	-0.90	1.06E-10	30.9%	15.8%	53.3%	5	424371
cg26701785	1.69	3.79E-11	72.9%	7.4%	19.7%	6	158448455
cg26703534	-4.01	7.91E-64	42.0%	23.6%	34.4%	5	377358
cg26764244	-1.69	3.10E-12	50.4%	15.1%	34.5%	1	68299511
cg27537125	-1.16	6.57E-19	20.5%	22.3%	57.3%	1	25349681
