# Supplementary material for: Characterizing genetic and environmental influences on variable DNA methylation using monozygotic and dizygotic twins
Source: PLoS Genet. 2018 Aug 9;14(8):e1007544. doi: 10.1371/journal.pgen.1007544 (PMC6084815; doi:10.1371/journal.pgen.1007544)
Supplement: S5 Table — (PDF) [file pgen.1007544.s005.pdf]

Probe ID	EWAS of BMI		A	C	E	Chromosome	Position
	change in BMI per unit increase in DNA methylation	P value					
cg09315878	-10.3 (1.7)	3.20E-08	19.25%	32.44%	48.31%	1	1152580
cg11832534	12.4 (2.0)	1.30E-08	58.31%	0.00%	41.69%	1	3563998
cg08648047	12.6 (1.9)	7.20E-10	26.70%	12.31%	61.00%	1	11028561
cg03885055	-25.2 (3.8)	1.30E-09	40.93%	6.87%	52.20%	1	16723232
cg12484113	18.6 (2.2)	1.20E-14	29.70%	21.38%	48.92%	1	27898757
cg16815882	21.2 (3.6)	6.00E-08	0.64%	44.86%	54.49%	1	35908609
cg17971578	-12.9 (2.2)	4.60E-08	22.92%	26.84%	50.24%	1	36852463
cg27547344	18.3 (3.1)	6.20E-08	19.08%	26.09%	54.83%	1	43765617
cg17901584	-13.7 (2.0)	1.40E-10	12.10%	41.36%	46.54%	1	55353706
cg16594806	-24.9 (2.6)	2.30E-18	38.77%	1.76%	59.47%	1	59473943
cg25001190	-10.8 (1.9)	8.10E-08	38.13%	15.60%	46.27%	1	61668835
cg03050965	-29.5 (4.9)	3.00E-08	12.63%	30.07%	57.30%	1	101705237
cg03725309	-24.8 (3.2)	3.80E-13	21.84%	35.41%	42.75%	1	109757585
cg14476101	-14.5 (1.6)	2.10E-17	72.13%	9.20%	18.68%	1	120255992
cg22700686	-25.9 (3.4)	3.20E-12	0.00%	35.89%	64.11%	1	153538764
cg12593793	-32.0 (2.7)	3.00E-27	6.18%	36.10%	57.71%	1	156074135
cg25217710	26.9 (3.4)	4.40E-13	81.58%	18.28%	0.14%	1	156609523
cg07202479	-14.5 (2.2)	2.20E-09	27.21%	46.17%	26.61%	1	159174162
cg09554443	-14.5 (2.1)	2.50E-10	39.93%	20.10%	39.97%	1	167487762
cg22534374	-7.3 (1.2)	1.70E-08	48.83%	11.03%	40.14%	1	201511610
cg10717869	21.2 (3.1)	1.80E-10	31.69%	19.68%	48.63%	1	205780912
cg15323828	-17.5 (2.6)	4.00E-10	54.02%	3.10%	42.88%	1	226053673
cg01101459	14.2 (2.1)	3.10E-10	15.03%	36.86%	48.11%	1	234871477
cg02560388	-11.4 (1.9)	4.70E-08	15.09%	16.62%	68.28%	2	11969958
cg04011474	-17.3 (2.7)	3.70E-09	34.23%	8.64%	57.14%	2	28904455

ch.2.30415474F	-35.4 (5.7)	7.70E-09	23.69%	27.73%	48.58%	2	30561970
cg16163382	-20.3 (2.6)	5.80E-13	17.38%	36.83%	45.79%	2	37938640
cg26253134	-23.8 (3.2)	3.20E-12	13.64%	39.66%	46.70%	2	70751721
cg25570328	-18.3 (2.9)	6.00E-09	17.85%	30.36%	51.79%	2	108903952
cg09152259	-10.3 (1.7)	4.70E-08	68.53%	17.60%	13.87%	2	128156114
cg15357118	15.3 (2.5)	1.60E-08	18.46%	28.43%	53.11%	2	128927972
cg03327570	-18.5 (2.5)	1.00E-11	23.57%	22.26%	54.17%	2	145304883
cg17178175	-13.8 (2.0)	5.10E-10	15.13%	29.72%	55.15%	2	178109973
cg09613192	10.6 (1.7)	7.30E-09	44.63%	21.83%	33.54%	2	181388538
cg00634542	15.6 (2.6)	2.10E-08	40.05%	0.00%	59.95%	2	219254588
cg00144180	15.4 (2.5)	1.50E-08	29.18%	26.02%	44.79%	2	240294362
cg23032421	-18.8 (3.1)	1.40E-08	28.80%	7.40%	63.81%	3	3152038
cg15681239	-11.3 (1.9)	5.50E-08	43.67%	14.49%	41.84%	3	38080203
cg00138407	14.1 (2.4)	4.50E-08	9.12%	29.16%	61.72%	3	47386505
cg00108715	21.3 (3.2)	4.00E-10	39.03%	20.85%	40.12%	3	52565015
cg22012981	10.2 (1.5)	3.50E-10	24.49%	15.72%	59.79%	3	58522689
cg10549088	10.5 (1.8)	7.70E-08	16.98%	37.71%	45.31%	3	64277154
cg12992827	-13.0 (1.9)	2.90E-10	20.12%	26.87%	53.02%	3	101901234
cg23232188	18.7 (2.5)	4.00E-12	59.87%	39.41%	0.72%	3	121556543
cg16846518	-15.4 (2.6)	6.70E-08	13.26%	37.23%	49.51%	3	128062608
cg25197194	-11.0 (1.8)	3.10E-08	51.82%	5.87%	42.30%	3	128758787
cg00673344	-16.0 (2.7)	5.30E-08	12.33%	27.37%	60.31%	3	156807691
cg18098839	-15.7 (2.2)	2.70E-11	42.91%	9.16%	47.94%	3	167742700
cg15721584	10.4 (1.3)	4.90E-14	34.96%	12.23%	52.82%	3	181326755
cg10513161	22.4 (3.7)	1.50E-08	45.88%	0.09%	54.03%	3	183705727
cg06164260	-21.3 (2.3)	8.10E-18	24.86%	34.07%	41.07%	3	187454439
cg18513344	-14.7 (2.0)	6.00E-12	32.91%	36.23%	30.86%	3	195531298
cg10438589	13.2 (2.3)	6.50E-08	9.55%	39.36%	51.08%	4	14531493
cg26542660	-22.1 (3.8)	8.50E-08	1.36%	37.23%	61.42%	4	56813860

cg06690548	-6.3 (0.9)	3.00E-10	25.18%	4.28%	70.53%	4	139162808
cg11080651	-26.0 (4.1)	2.90E-09	0.00%	44.16%	55.84%	5	10445523
cg10179300	17.1 (2.6)	9.10E-10	49.78%	8.85%	41.37%	5	14147618
cg04232128	-23.0 (3.8)	3.00E-08	3.30%	34.98%	61.73%	5	138861241
cg26403843	10.7 (1.3)	2.90E-15	65.05%	2.65%	32.30%	5	158634085
cg11927233	10.5 (1.6)	1.30E-09	36.58%	37.60%	25.82%	5	170816542
cg02286155	23.2 (3.3)	1.20E-10	18.45%	19.31%	62.24%	5	176826262
cg22590032	13.9 (2.1)	2.50E-09	0.00%	46.89%	53.11%	5	180050565
cg10975897	-12.9 (2.1)	8.20E-09	19.27%	31.00%	49.73%	6	15504844
cg00094412	-16.6 (2.7)	1.10E-08	25.15%	27.77%	47.08%	6	29592854
cg13123009	18.4 (2.9)	3.40E-09	52.89%	8.59%	38.52%	6	31681882
cg03957124	-22.3 (3.4)	1.60E-09	46.08%	53.04%	0.88%	6	37016869
cg18120259	-12.6 (1.9)	1.60E-09	18.91%	28.43%	52.66%	6	43894639
cg06012428	-20.8 (3.3)	3.70E-09	23.93%	25.73%	50.34%	6	157477204
cg03940776	-25.9 (4.0)	3.00E-09	13.13%	36.59%	50.28%	6	158490013
cg17501210	-18.4 (1.9)	8.20E-19	69.51%	5.38%	25.11%	6	166970252
cg05095590	9.5 (1.4)	9.60E-10	65.22%	4.91%	29.86%	7	2139259
cg26804423	20.5 (3.0)	2.40E-10	6.50%	31.13%	62.36%	7	8201134
cg24469729	16.2 (2.7)	2.30E-08	60.41%	2.41%	37.19%	7	27160520
cg21429551	-6.9 (1.0)	6.20E-10	40.97%	35.69%	23.34%	7	30635762
cg04577162	20.3 (3.2)	2.60E-09	32.79%	45.44%	21.76%	7	73667397
cg19566658	13.5 (2.2)	5.90E-09	41.49%	18.90%	39.61%	7	100466241
cg22103219	-14.9 (2.0)	7.00E-12	17.92%	33.21%	48.86%	7	101934892
cg05720226	18.9 (3.1)	1.40E-08	56.78%	8.17%	35.05%	7	116786597
cg27269962	22.3 (3.4)	1.50E-09	13.93%	22.80%	63.27%	7	127540997
cg25435714	16.5 (2.5)	5.30E-10	20.86%	37.41%	41.72%	7	157083381
cg24531955	-11.0 (1.6)	3.20E-10	40.22%	18.04%	41.74%	8	23154691
cg19589396	-14.3 (2.1)	2.30E-10	49.31%	1.18%	49.52%	8	103937374
cg07471614	21.3 (3.4)	9.40E-09	65.44%	0.00%	34.56%	8	125855152

cg26952928	18.1 (2.8)	3.60E-09	29.33%	17.20%	53.46%	8	142230233
cg26361535	11.0 (1.6)	1.40E-10	59.28%	9.85%	30.87%	8	144576604
cg02716826	-14.2 (1.9)	1.60E-12	53.03%	39.73%	7.23%	9	33447032
cg13591783	-14.3 (2.5)	9.00E-08	6.39%	46.81%	46.81%	9	75768868
cg14264316	12.5 (2.0)	1.10E-08	0.00%	24.08%	75.92%	9	134280803
cg13781414	-12.0 (2.0)	4.30E-08	13.65%	38.11%	48.25%	9	138951648
cg19695507	9.8 (1.5)	7.80E-10	42.93%	0.00%	57.07%	10	13526193
cg26033520	11.9 (1.7)	1.60E-10	18.56%	13.53%	67.90%	10	74004071
cg04126866	27.4 (4.6)	3.00E-08	22.75%	26.19%	51.06%	10	85932763
cg16578636	-7.0 (1.2)	8.80E-08	62.19%	13.94%	23.87%	10	92987457
cg07504977	12.4 (2.0)	4.50E-09	44.35%	6.54%	49.11%	10	102131012
cg00431050	-26.8 (3.8)	1.10E-10	76.09%	22.76%	1.15%	10	103985730
cg26878209	14.9 (2.5)	4.30E-08	11.43%	50.21%	38.37%	10	112375475
cg00244001	-33.3 (4.4)	2.00E-12	14.41%	37.51%	48.08%	10	126336805
cg00238353	10.2 (1.8)	9.20E-08	28.47%	0.00%	71.53%	10	129785537
cg10927968	12.8 (1.8)	3.90E-11	19.30%	35.05%	45.65%	11	1807333
cg06603309	-27.7 (3.8)	1.40E-11	22.27%	40.05%	37.68%	11	2724144
cg07136133	-19.8 (2.8)	6.50E-11	21.82%	36.54%	41.65%	11	36422377
cg21108085	-18.1 (3.1)	5.00E-08	0.00%	39.81%	60.19%	11	44591098
cg05648472	16.8 (2.4)	6.10E-11	55.81%	4.70%	39.50%	11	45232364
cg11376147	-25.3 (3.0)	5.80E-15	22.80%	30.14%	47.05%	11	57261198
cg03433986	-18.6 (2.9)	4.50E-09	44.18%	0.00%	55.82%	11	62477624
cg09777883	15.7 (2.5)	1.30E-08	31.72%	22.35%	45.93%	11	112093696
cg17260706	-28.4 (3.3)	1.20E-15	23.68%	16.87%	59.45%	11	118782879
cg26894079	-14.2 (2.2)	2.20E-09	14.54%	37.78%	47.68%	11	122954435
cg22488164	14.4 (2.1)	2.50E-10	44.32%	7.37%	48.31%	12	14716910
cg06898549	9.8 (1.6)	1.80E-08	22.32%	7.77%	69.91%	12	41083590
cg06559575	-21.1 (3.5)	2.50E-08	7.08%	33.10%	59.82%	12	53490352
cg05845030	-19.3 (2.9)	1.10E-09	36.02%	63.58%	0.40%	12	91573247

cg27117792	-12.9 (2.2)	4.50E-08	15.29%	36.07%	48.65%	12	102330180
cg01511901	-17.5 (3.0)	8.00E-08	34.26%	22.33%	43.42%	13	31004719
cg19750657	23.9 (2.7)	2.20E-16	54.40%	0.00%	45.60%	13	38935967
cg26687842	14.2 (2.4)	2.60E-08	77.72%	20.76%	1.52%	13	41055491
cg11650298	-26.6 (3.8)	1.70E-10	10.16%	13.03%	76.81%	13	44690989
cg19881557	9.8 (1.6)	3.70E-08	34.22%	16.00%	49.78%	14	20967426
cg03523676	19.5 (2.5)	3.50E-13	42.45%	19.84%	37.71%	14	24540235
cg26357885	-21.8 (3.2)	5.70E-10	19.95%	23.38%	56.67%	14	65006204
cg10919522	-14.1 (2.1)	7.50E-10	27.99%	12.76%	59.25%	14	74227441
cg19998073	18.6 (2.8)	1.30E-09	46.64%	0.00%	53.36%	14	89078443
cg10814005	-14.1 (2.4)	5.10E-08	28.50%	9.75%	61.75%	14	91711041
cg10734665	-13.2 (2.1)	1.30E-08	50.03%	18.37%	31.61%	15	26107410
cg27184903	15.9 (2.7)	6.80E-08	20.15%	76.23%	3.62%	15	29285727
cg06192883	15.8 (2.5)	3.90E-09	45.72%	2.71%	51.56%	15	52554171
cg07037944	-21.4 (3.0)	4.90E-11	32.23%	30.19%	37.58%	15	64290807
cg02119938	-23.4 (3.2)	2.90E-11	58.04%	0.00%	41.96%	15	78505051
cg07728579	14.9 (2.3)	1.70E-09	28.52%	13.74%	57.74%	15	83475013
cg11183227	18.9 (2.8)	8.10E-10	33.64%	14.38%	51.98%	15	91455407
cg27614723	24.5 (3.6)	2.70E-10	18.59%	24.82%	56.59%	15	92399897
cg00973118	9.8 (1.6)	2.30E-08	54.17%	17.60%	28.23%	16	374570
cg05063895	-29.8 (4.7)	6.30E-09	0.62%	35.43%	63.96%	16	2073518
cg06946797	-14.5 (1.8)	1.50E-13	20.02%	29.77%	50.20%	16	11422409
cg26663590	16.6 (2.3)	1.30E-11	7.44%	33.04%	59.52%	16	28959310
cg00711896	20.5 (3.2)	3.10E-09	41.59%	8.76%	49.66%	16	30410051
cg01243823	-13.8 (1.8)	1.70E-12	57.70%	22.62%	19.67%	16	50732212
cg00863378	14.8 (2.4)	2.40E-08	42.43%	8.30%	49.27%	16	56549757
cg10922280	23.8 (3.1)	6.10E-13	4.09%	44.59%	51.32%	16	68034227
cg08305942	-15.3 (2.6)	3.30E-08	52.29%	0.00%	47.71%	16	79692354
cg03159676	2.1 (0.4)	3.50E-08	60.06%	15.82%	24.12%	16	85600536

cg07021906	14.2 (2.1)	3.20E-10	34.28%	17.34%	48.38%	16	87866833
cg08443038	-14.3 (2.4)	4.20E-08	28.52%	24.74%	46.74%	16	89006877
cg08726900	-9.9 (1.5)	2.00E-09	50.37%	0.00%	49.63%	16	89550474
cg09664445	31.2 (3.5)	2.40E-16	14.56%	42.94%	42.50%	17	2612406
cg11024682	32.5 (3.0)	1.90E-23	35.80%	32.92%	31.28%	17	17730094
cg16611584	7.1 (1.0)	1.00E-11	76.04%	3.88%	20.08%	17	19809078
cg25649826	16.5 (2.7)	8.90E-09	22.86%	26.25%	50.88%	17	20938740
cg13274938	22.8 (3.7)	9.20E-09	30.87%	2.70%	66.43%	17	38493822
cg08857797	19.8 (2.4)	3.30E-14	13.02%	27.75%	59.23%	17	40927699
cg18219562	19.3 (3.1)	5.70E-09	30.53%	3.33%	66.14%	17	41773643
cg27050612	-33.2 (5.2)	4.30E-09	4.27%	40.87%	54.86%	17	46133198
cg24174557	-11.6 (1.5)	2.40E-12	4.44%	33.36%	62.20%	17	57903544
cg08813944	14.0 (2.4)	7.60E-08	23.25%	32.66%	44.09%	17	71258589
cg14020176	17.5 (3.0)	4.60E-08	1.56%	48.14%	50.30%	17	72764985
cg21486834	17.9 (2.4)	6.40E-12	41.78%	12.32%	45.90%	17	74477542
cg18181703	-18.0 (1.8)	2.80E-20	64.28%	0.00%	35.72%	17	76354621
cg11202345	17.8 (2.5)	2.30E-11	53.71%	0.00%	46.29%	17	76976057
cg11969813	17.5 (2.4)	1.60E-11	7.20%	42.29%	50.52%	17	79816559
cg18608055	-21.7 (2.8)	5.90E-13	41.49%	58.09%	0.42%	19	1130866
cg04524040	-13.5 (2.3)	4.10E-08	50.71%	6.17%	43.12%	19	4153364
cg07769588	8.4 (1.1)	1.30E-12	58.83%	10.72%	30.45%	19	10655622
cg13922488	21.2 (3.6)	4.80E-08	19.20%	35.62%	45.18%	19	14545201
cg24679890	22.3 (3.2)	8.30E-11	26.51%	16.87%	56.62%	19	17246356
cg07682160	16.8 (2.6)	1.50E-09	34.96%	63.06%	1.98%	19	18959935
cg26836479	-25.7 (4.2)	1.50E-08	16.88%	34.83%	48.29%	19	42706353
cg27087650	-21.5 (3.3)	2.10E-09	10.67%	35.22%	54.11%	19	45255796
cg02711608	-17.2 (2.4)	5.60E-11	19.34%	47.82%	32.84%	19	47287964
cg11614585	16.1 (2.8)	9.00E-08	11.47%	38.12%	50.41%	20	897050
cg18217136	19.8 (3.3)	3.80E-08	43.68%	6.49%	49.82%	20	36157651

cg24403644	26.5 (4.5)	5.90E-08	11.55%	30.82%	57.63%	20	42574624
cg08309687	-15.2 (1.9)	1.10E-13	21.21%	30.49%	48.30%	21	35320596
cg08548559	-9.0 (1.4)	3.40E-09	65.38%	7.76%	26.86%	22	31686097
cg27115863	-13.6 (2.2)	1.10E-08	66.20%	32.23%	1.57%	22	37921640
cg03318904	22.3 (2.9)	2.20E-12	22.74%	20.26%	57.00%	22	39801522
cg09349128	-24.4 (2.4)	1.70E-20	24.08%	24.61%	51.31%	22	50327986
