# Supplementary material for: Characterizing genetic and environmental influences on variable DNA methylation using monozygotic and dizygotic twins
Source: PLoS Genet. 2018 Aug 9;14(8):e1007544. doi: 10.1371/journal.pgen.1007544 (PMC6084815; doi:10.1371/journal.pgen.1007544)
Supplement: S1 Fig — Shown are violin plots for the average correlations of DNA methylation within each sibling pair (stratified by relatedness) averaged across A) all autosomal DNA methylation sites (n = 420,857), B) autosomal sites characterized by “variable” DNA methylation (n = 214,991), C) autosomal sites characterized by “non-variable” DNA methylation (n = 205,866), D) autosomal sites with intermediate levels of DNA methylation (n = 131,728), and E) autosomal sites characterized as being either hypo- or hyper-methylated (n = 289,129). P-values are from a t-test comparing average correlations observed in MZ twins to those observed in DZ twins. Also shown are comparisons between random pairs of unrelated individuals selected from the E-Risk cohort. (PDF) [file pgen.1007544.s006.pdf]

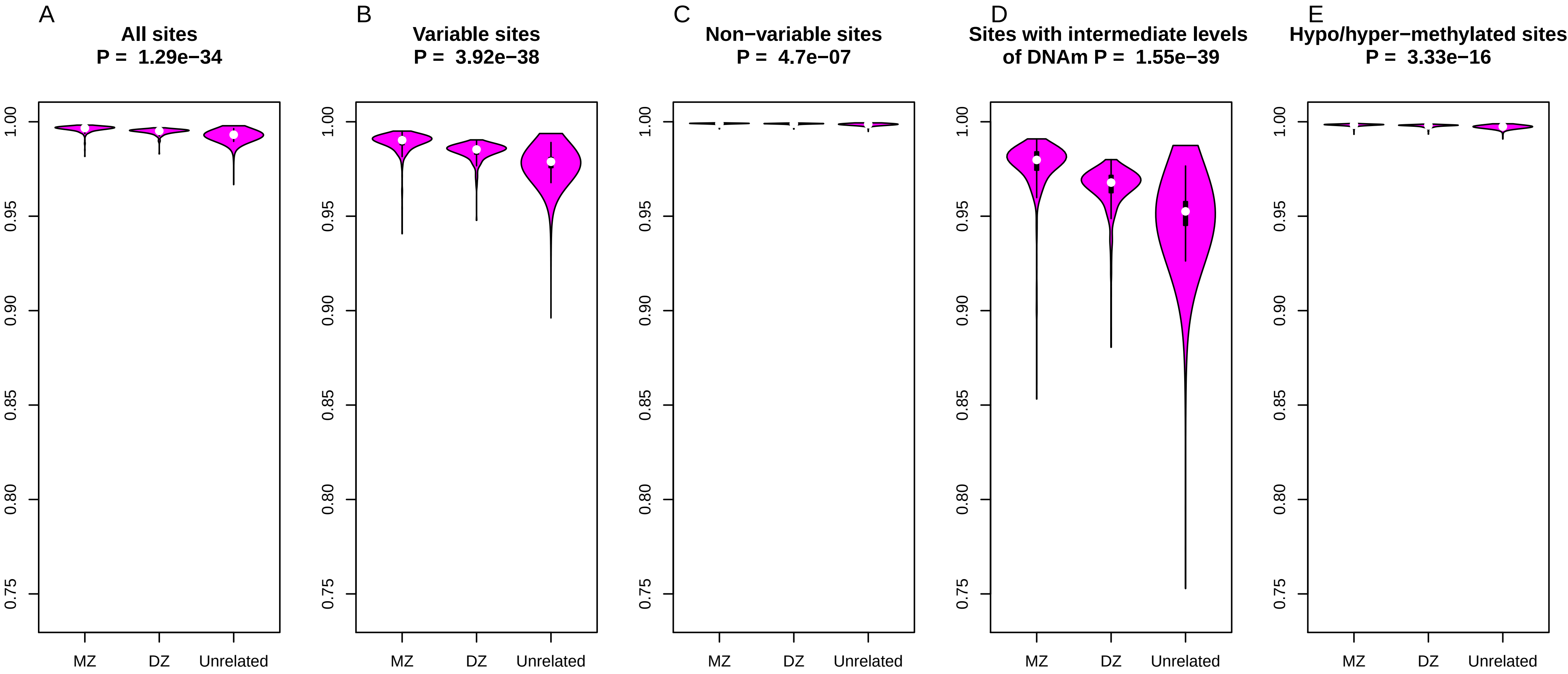
A
All sites
P = 1.29e−34
B
Variable sites
P = 3.92e−38
C
Non−variable sites
P = 4.7e−07
D
Sites with intermediate levels
of DNAm P = 1.55e−39
E
Hypo/hyper−methylated sites
P = 3.33e−16
1.00
0.95
0.90
0.85
0.80
0.75
MZ
DZ
Unrelated
