# Supplementary figures and images for: Characterizing genetic and environmental influences on variable DNA methylation using monozygotic and dizygotic twins
Source: PLoS Genet. 2018 Aug 9;14(8):e1007544. doi: 10.1371/journal.pgen.1007544 (PMC6084815; doi:10.1371/journal.pgen.1007544)

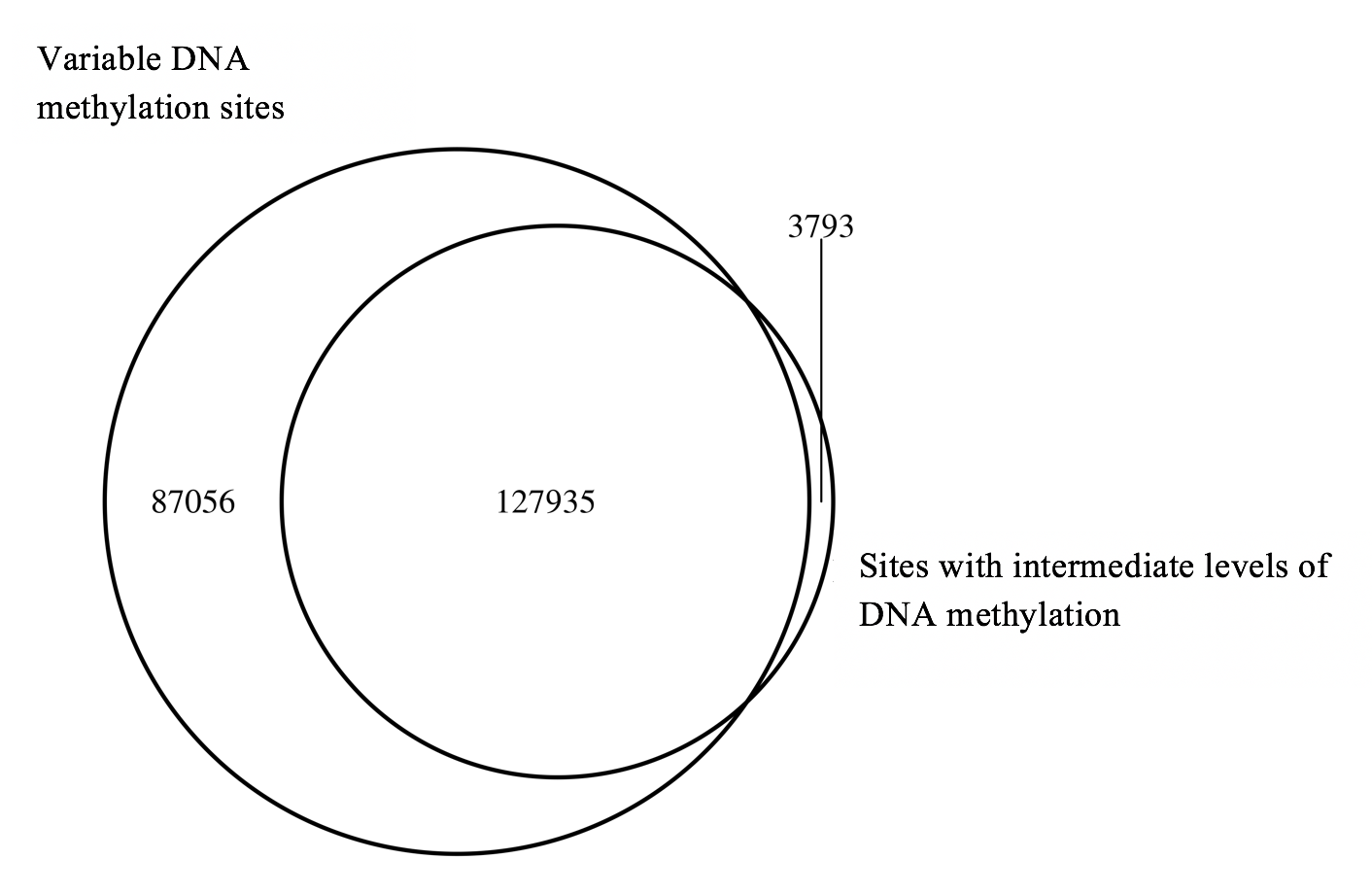

Supplement: S2 Fig — (TIFF) [file pgen.1007544.s007.tiff]

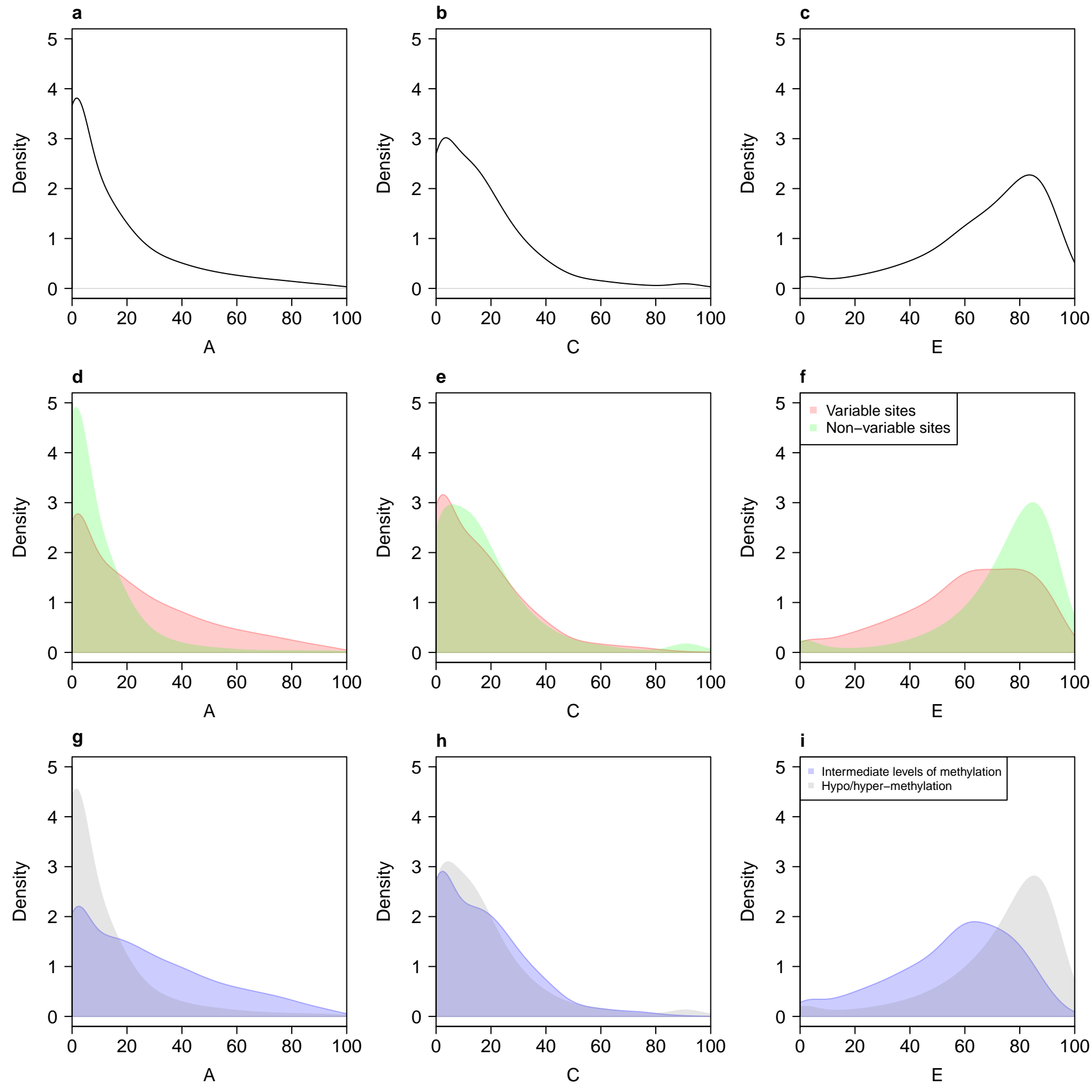

Supplement: S3 Fig — The proportion of variance in DNA methylation explained by additive genetic effects (A), shared environmental effects (C) and unshared (or unique) environmental effects (E) across autosomal sites after adjusting for cellular composition. Panels a-c show density distributions for estimates of A, C, and E across all 420,857 autosomal DNA methylation sites. At the majority of autosomal sites, environmental factors contribute more to the observed variance in DNA methylation than additive genetic factors. We observe significantly higher average heritability estimates for DNA methylation across the subset of DNA methylation sites defined as “variable” (d-f) (mean A = 29.3% (SD = 25.0%); Mann Whitney P < 2.2x10^-16^) and (g-i) sites with intermediate levels of DNA methylation (mean A = 24.3% (SD = 24.2%); Mann Whitney P < 2.2x10^-16^). (PDF) [file pgen.1007544.s008.pdf]

A:  $r = 0.938$

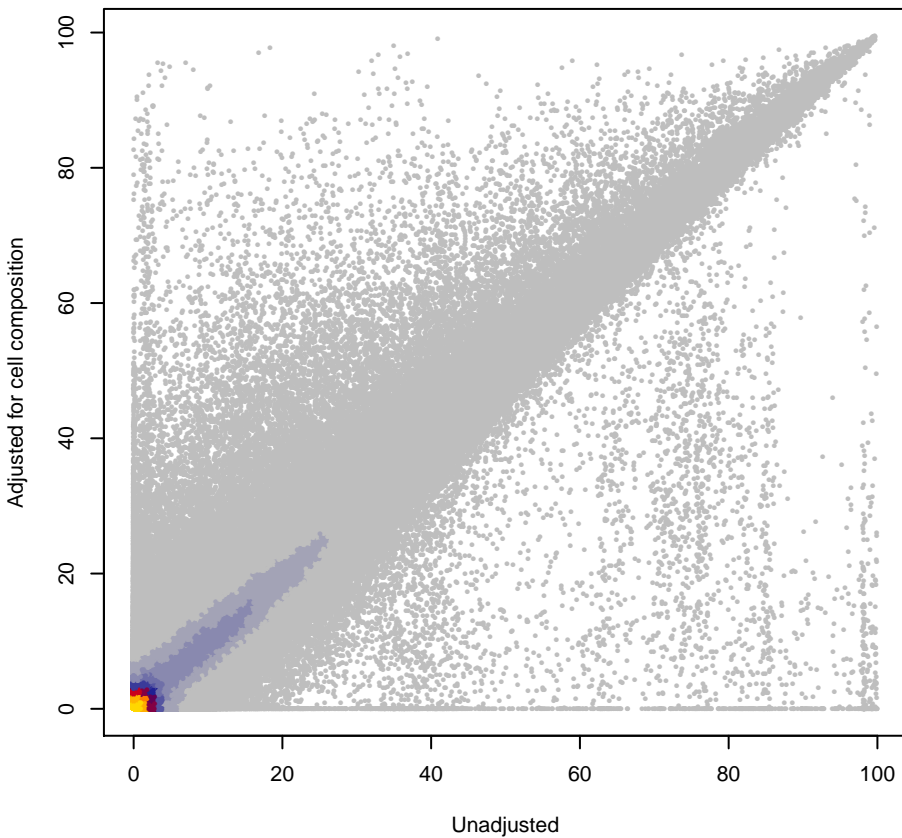

C:  $r = 0.826$

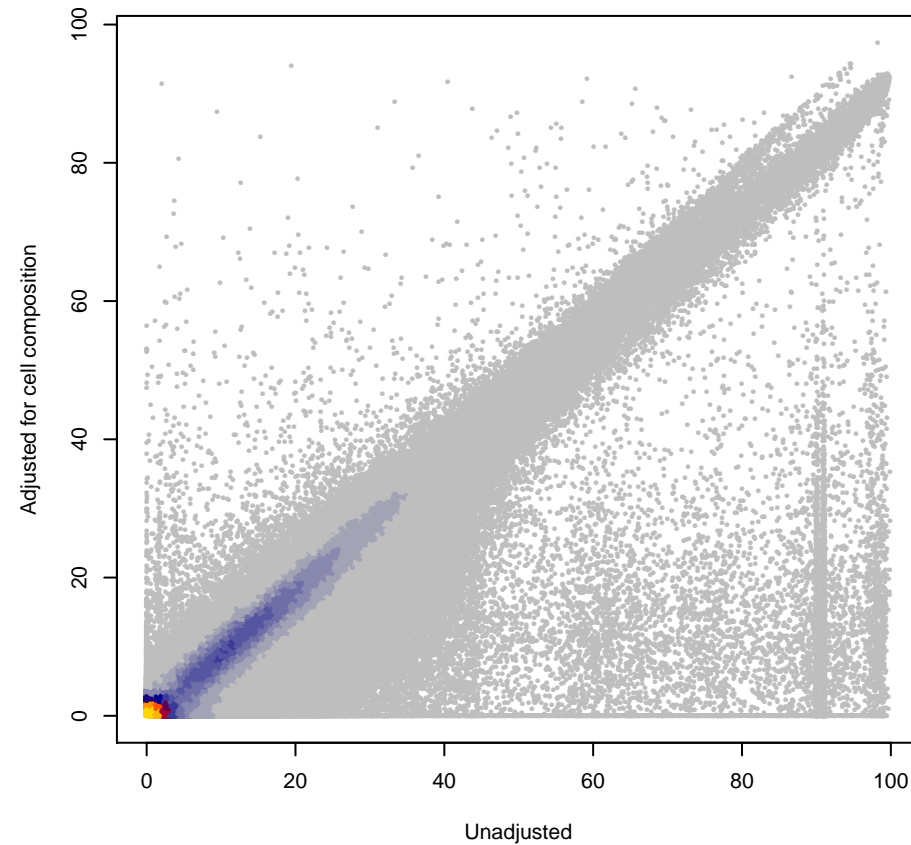

E:  $r = 0.881$

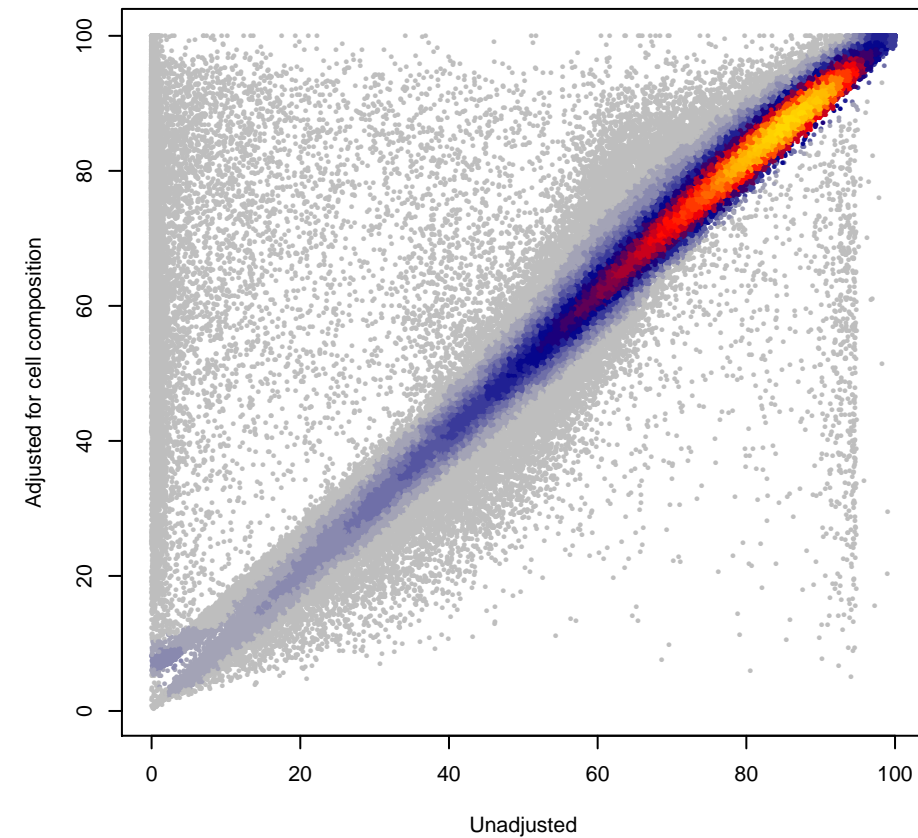

Supplement: S4 Fig — Scatterplots of additive genetic effects (A), shared environmental effects (C) and non-shared (or unique) environmental effects (E) for all autosomal DNA methylation sites (n = 420,857), comparing DNA methylation data unadjusted for cellular composition (x-axis) and DNA methylation data adjusted for cellular composition variables (y-axis). Each point represents a DNA methylation site and the colour of the point indicates the density of points at that location (gray–low to yellow–high). (PDF) [file pgen.1007544.s009.pdf]

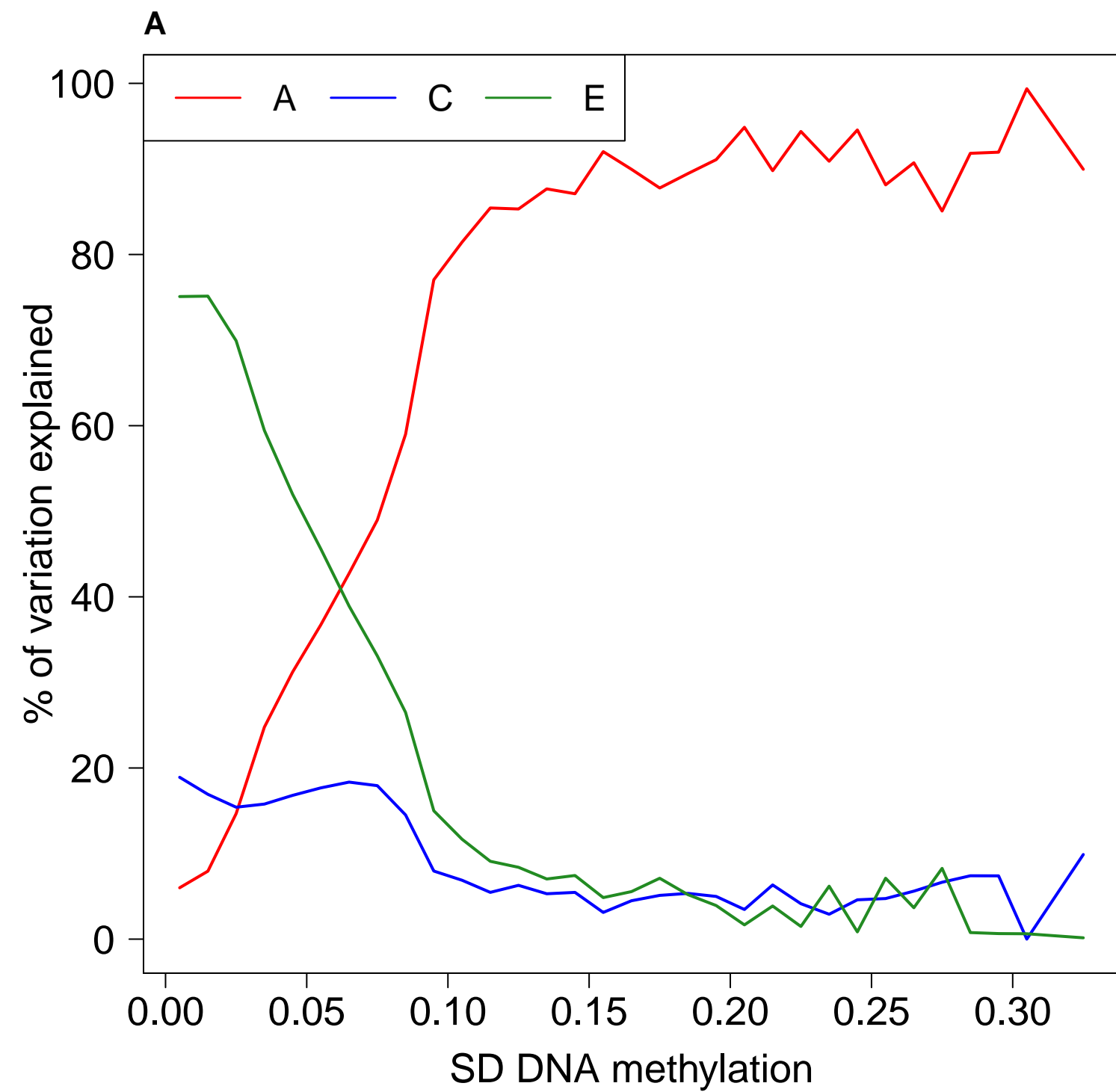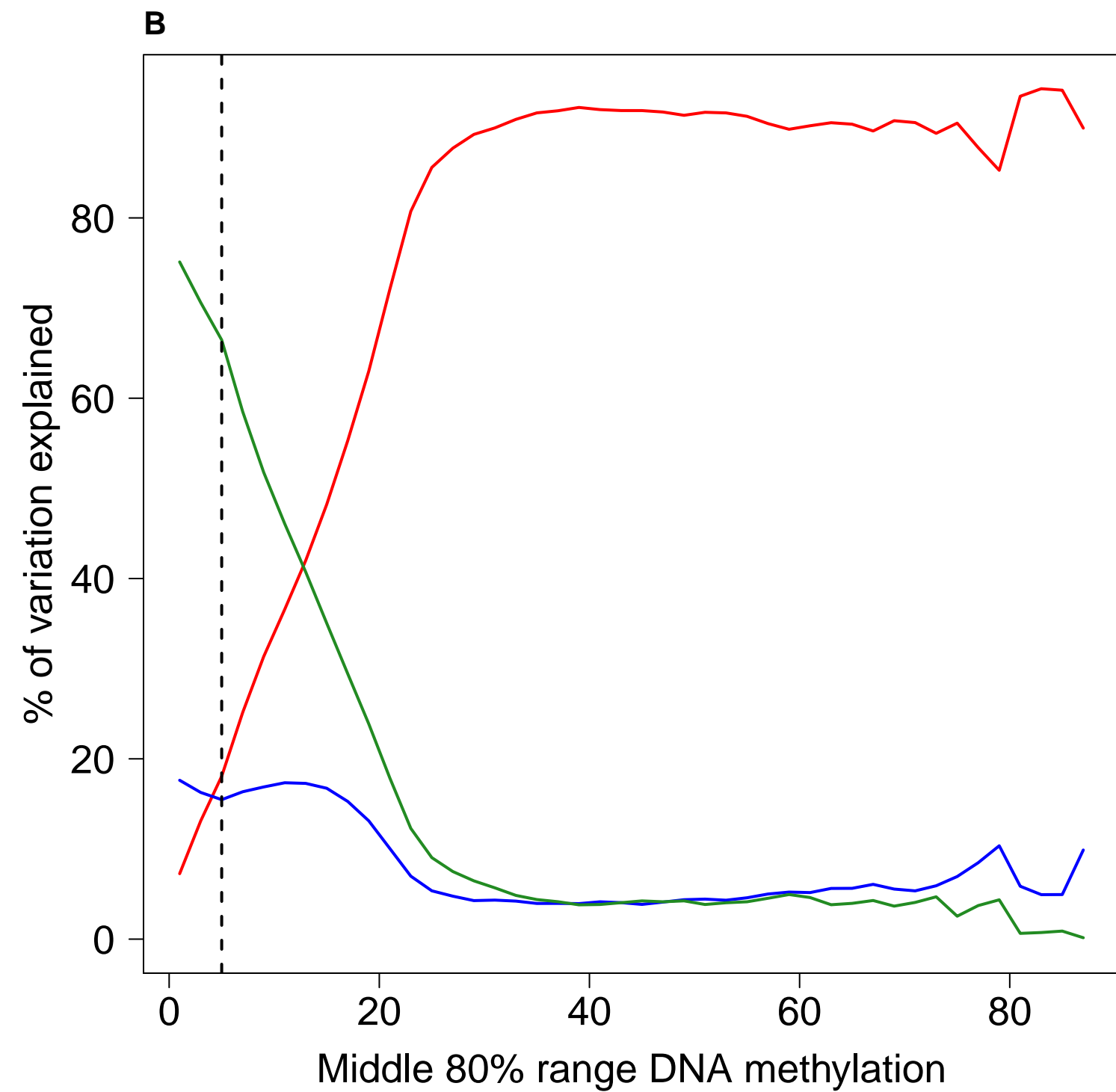

Supplement: S5 Fig — Shown are estimates of additive genetic effects (A), shared environmental effects (C) and non-shared (or unique) environmental effects (E) plotted as a function of the variability in DNA methylation measured by A) the standard deviation (SD) and B) the range of the middle 80% of the distribution of DNA methylation levels. In panel B, the dashed vertical line indicates the cut-off (5%) used to define DNA methylation sites as being “variable” in this study. (PDF) [file pgen.1007544.s010.pdf]

A

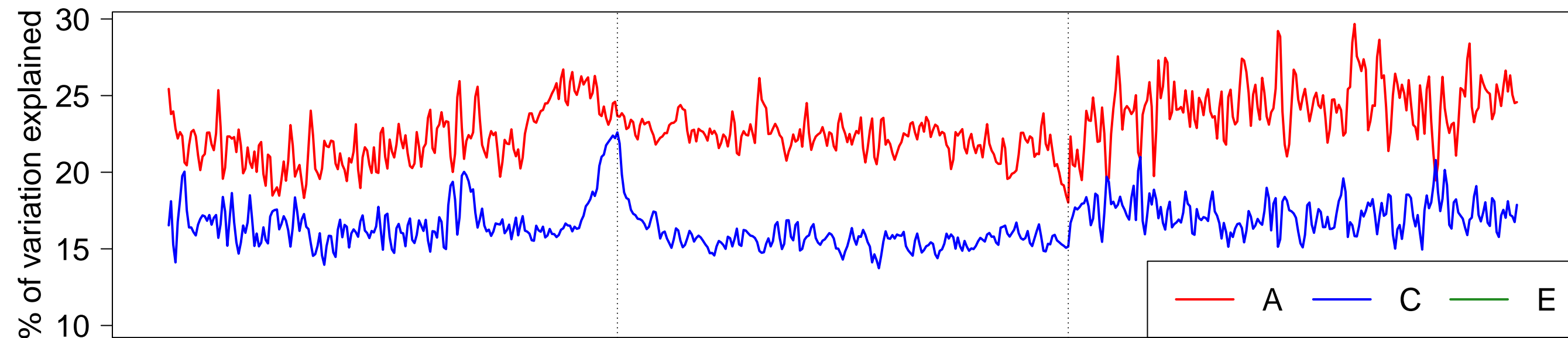

B

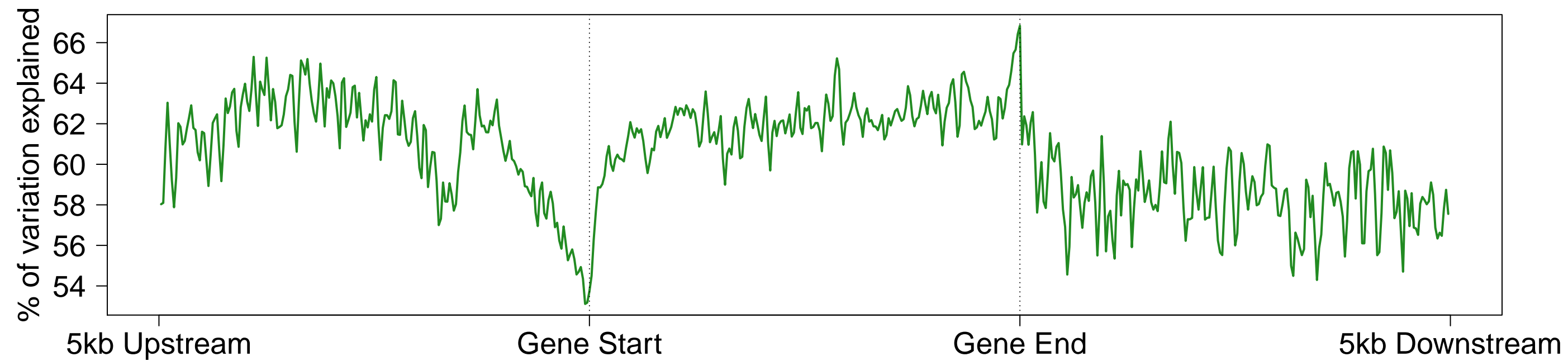

Supplement: S6 Fig — Shown is a line graph depicting the extent to which variation in DNA methylation is influenced by genetic and environmental factors across a canonical gene region. Genetic influences on DNA methylation are highest immediately upstream of the transcription start-site (TSS), and in the region spanning 5 kilobases downstream of the gene coding sequence (red line, panel A). Sites located around the TSS are enriched for shared environmental effects (blue line, panel A) and show reduced non-shared environmental effects (green line, panel B). (PDF) [file pgen.1007544.s011.pdf]

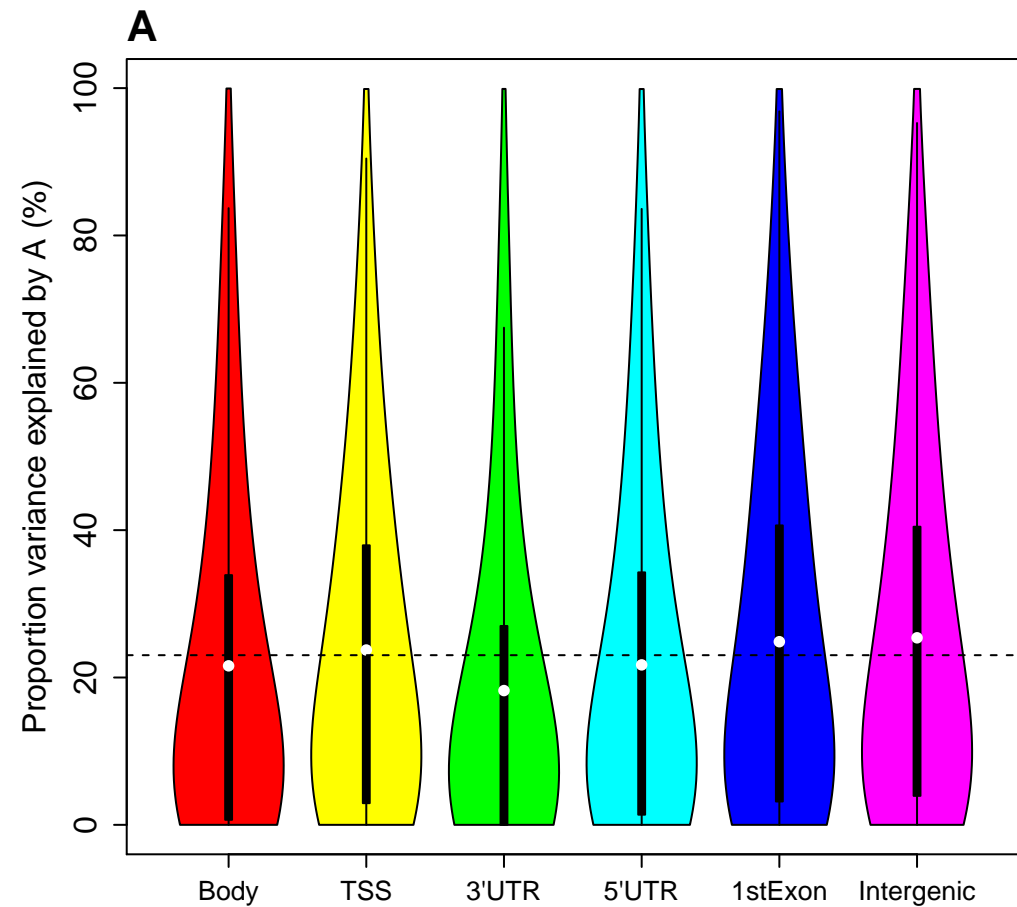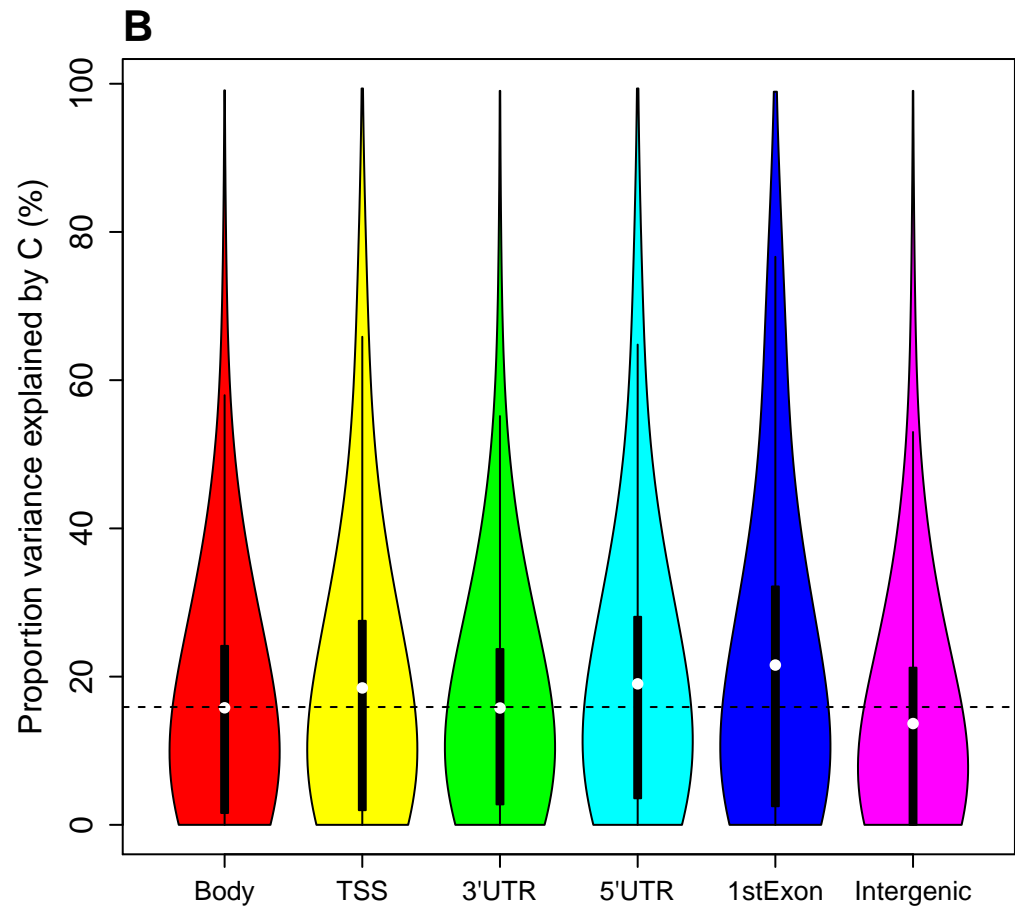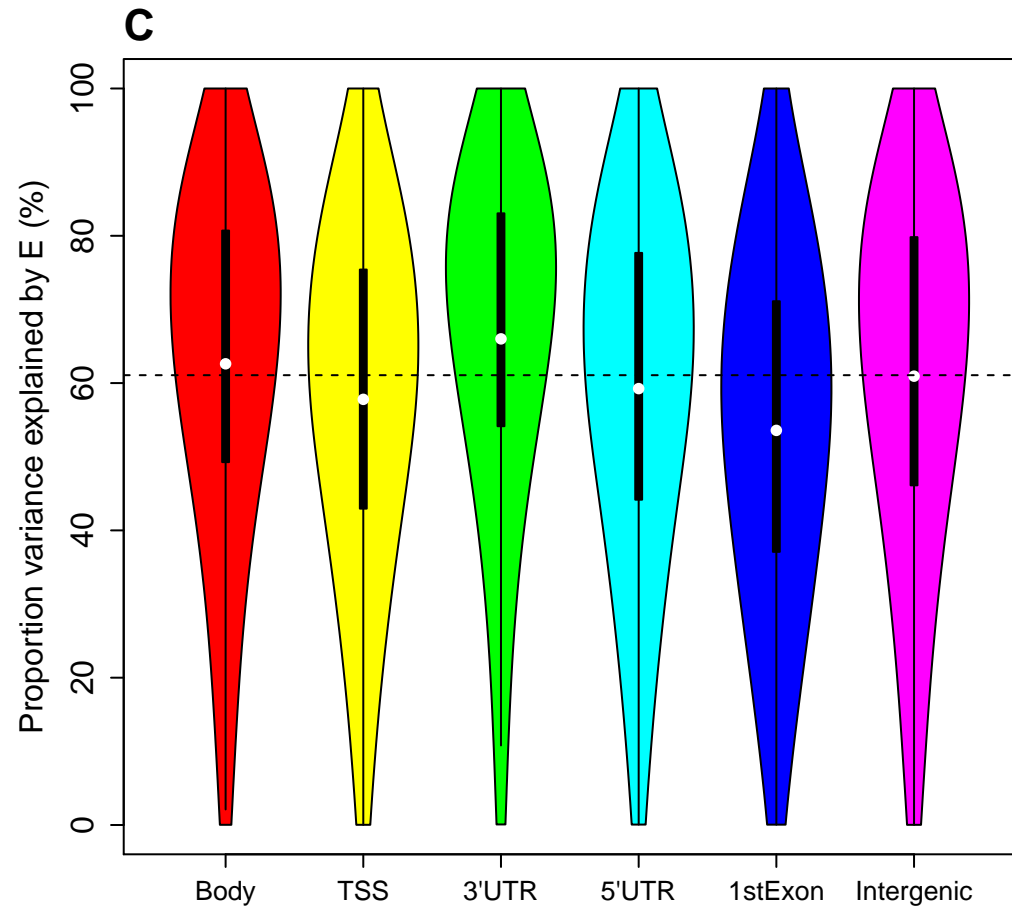

Supplement: S7 Fig — Shown is a density plot of estimates of A) additive genetic, B) shared environmental, and C) non-shared environmental influences on DNA methylation at autosomal sites stratified by gene feature annotation. (PDF) [file pgen.1007544.s012.pdf]

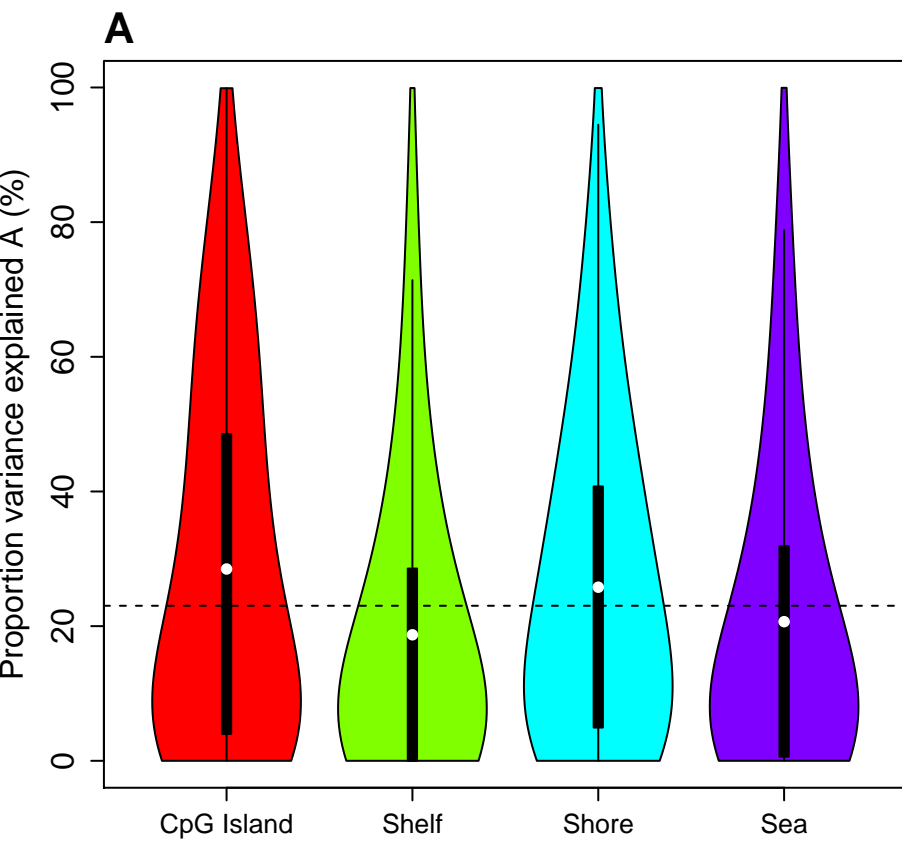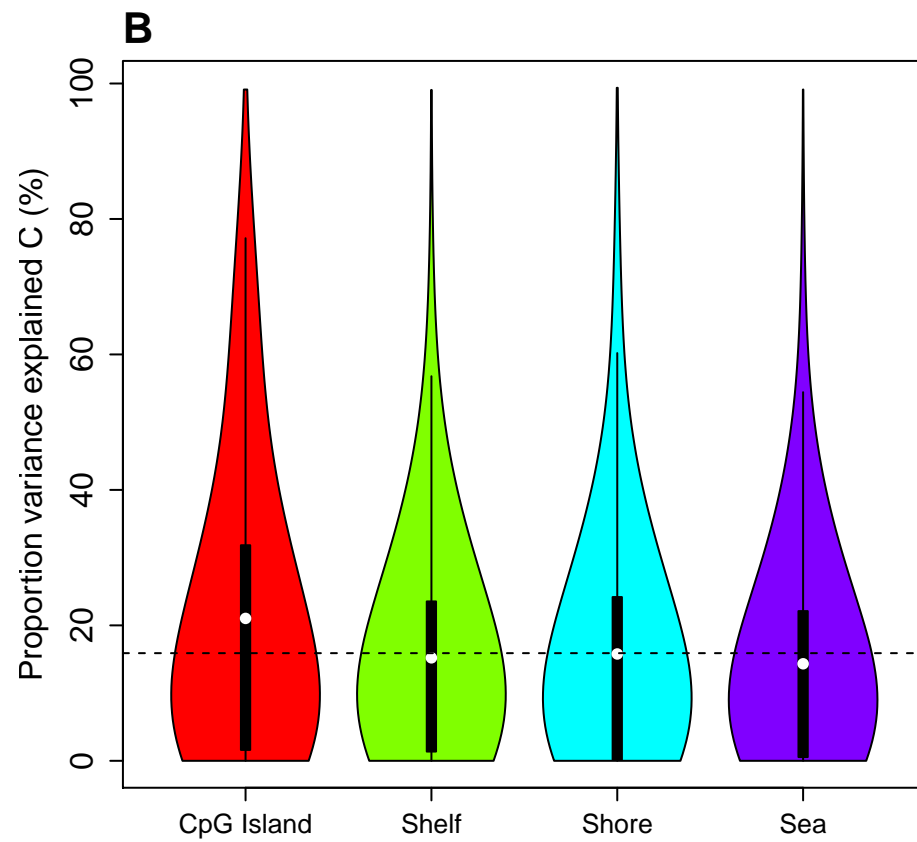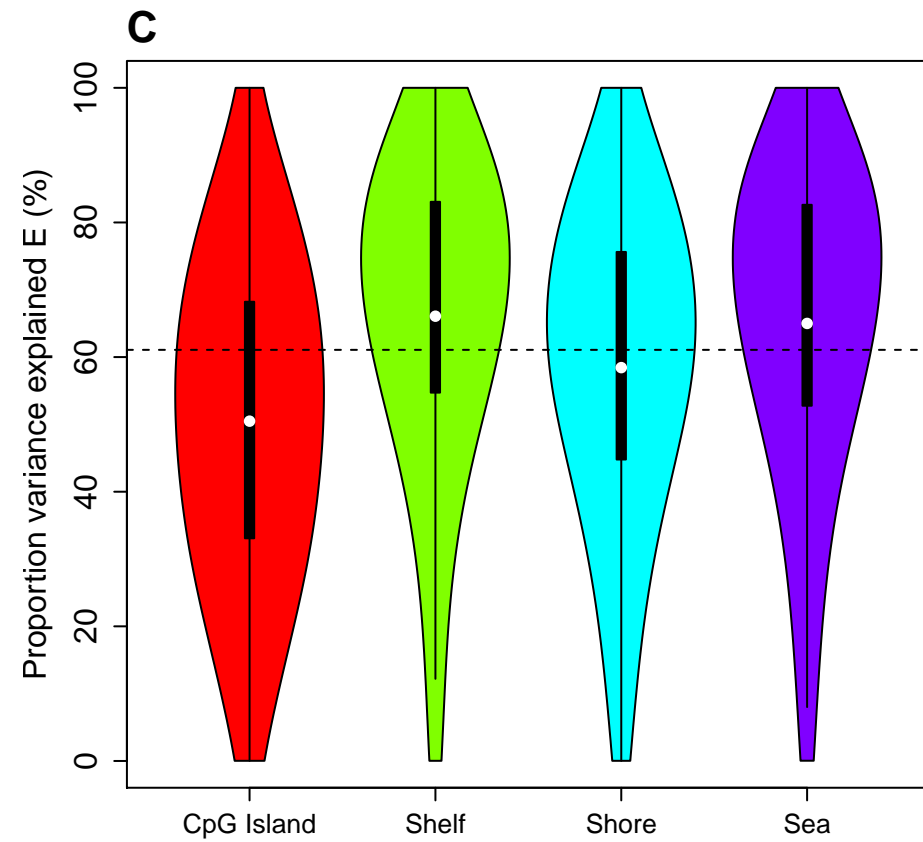

Supplement: S8 Fig — Shown is a density plot of estimates of A) additive genetic, B) shared environmental, and C) non-shared environmental influences on DNA methylation at sites stratified by CpG island feature annotation. (PDF) [file pgen.1007544.s013.pdf]

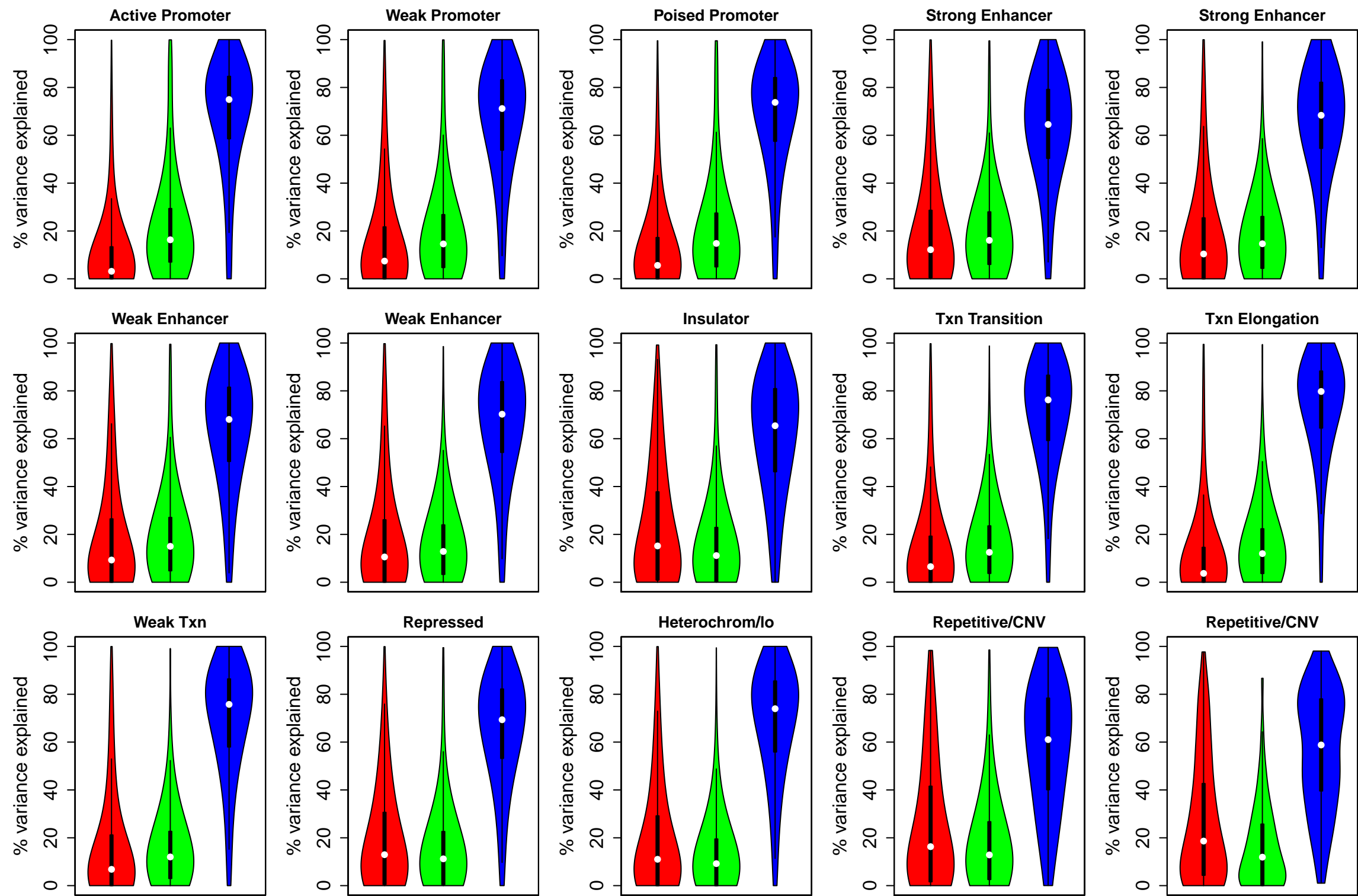

Supplement: S9 Fig — Violin plots showing the proportion of variance explained by additive genetic factors (A; red), common environmental factors (C; green), and unique environmental factors (E; blue) where DNA methylation sites are stratified by their location in regulatory annotation states as defined by ChromHMM [59] using ENCODE experimental data from the GM12878 cell line. (PDF) [file pgen.1007544.s014.pdf]

% DNA methylation change per allele

15

10

5

0

20

40

60

80

100

Mean DNA methylation %

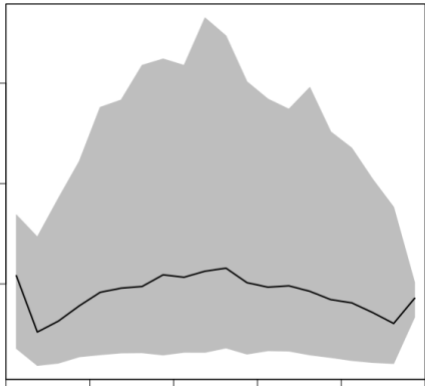

Supplement: S10 Fig — Line graph of the moving mean mQTL effect on DNA methylation (measured as the % DNA methylation change per allele; y-axis) as a function of mean DNA methylation (%; x-axis). The gray area indicates the 95% interquantile range for the moving average. (PDF) [file pgen.1007544.s015.pdf]

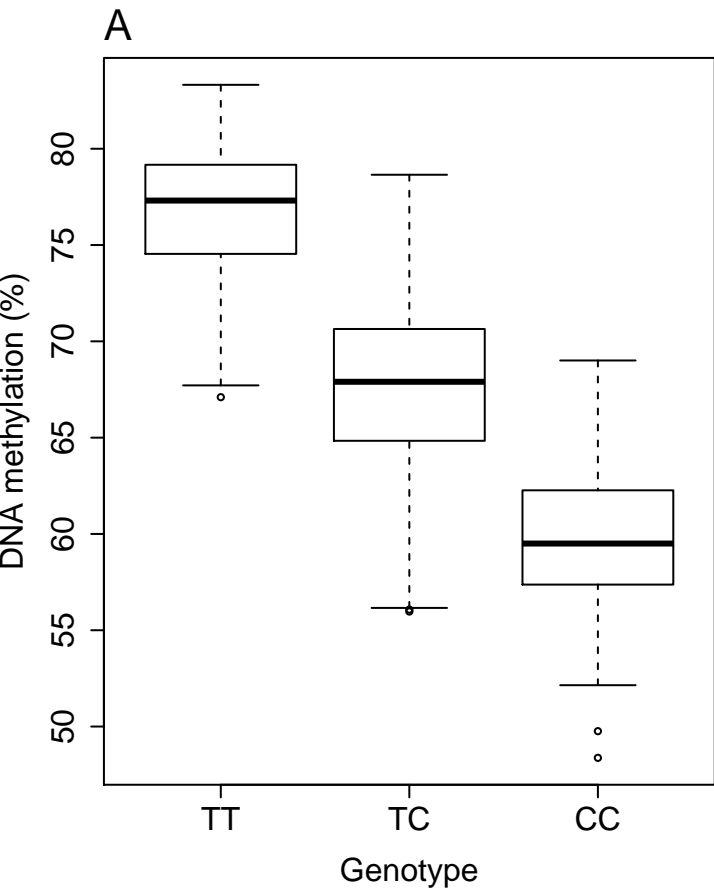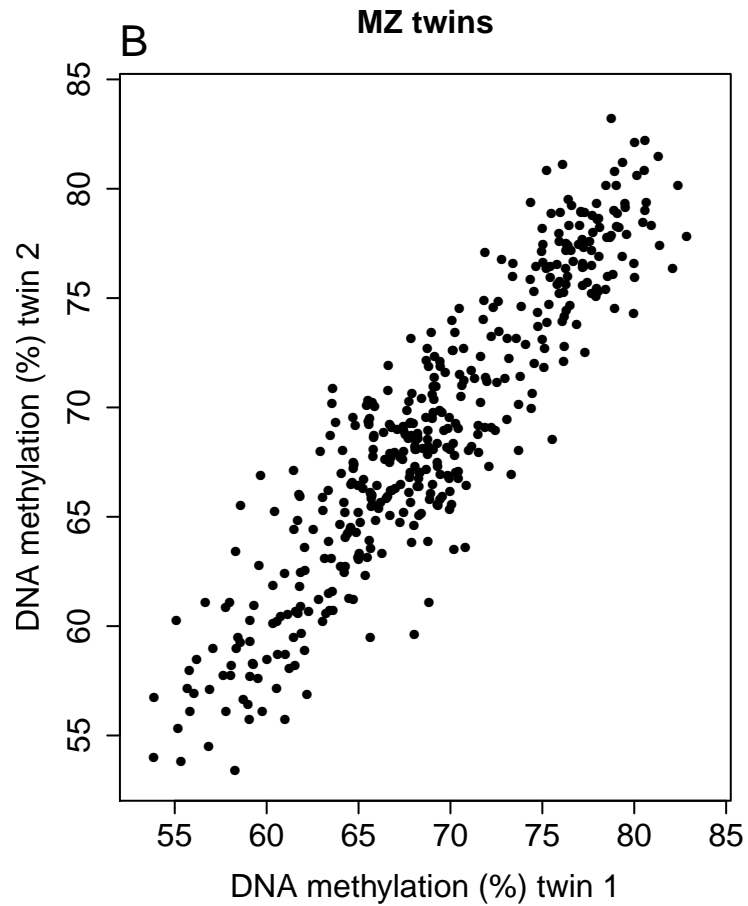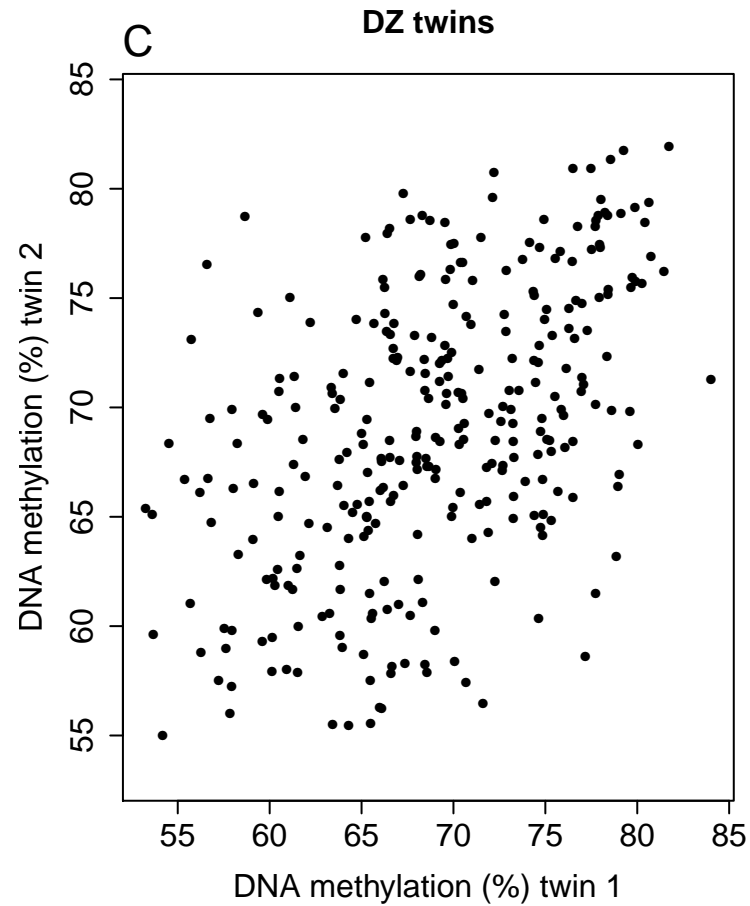

Supplement: S11 Fig — Panel A) shows a boxplot of the association between DNA methylation at cg02573566 and genotype at rs11548104 (P = 5.95x10-179). Panel B) shows the correlation in DNA methylation at cg02573566 between MZ twins (r = 0.916) and panel C) shows the correlation in DNA methylation at cg02573566 between DZ twins (r = 0.487). (PDF) [file pgen.1007544.s016.pdf]

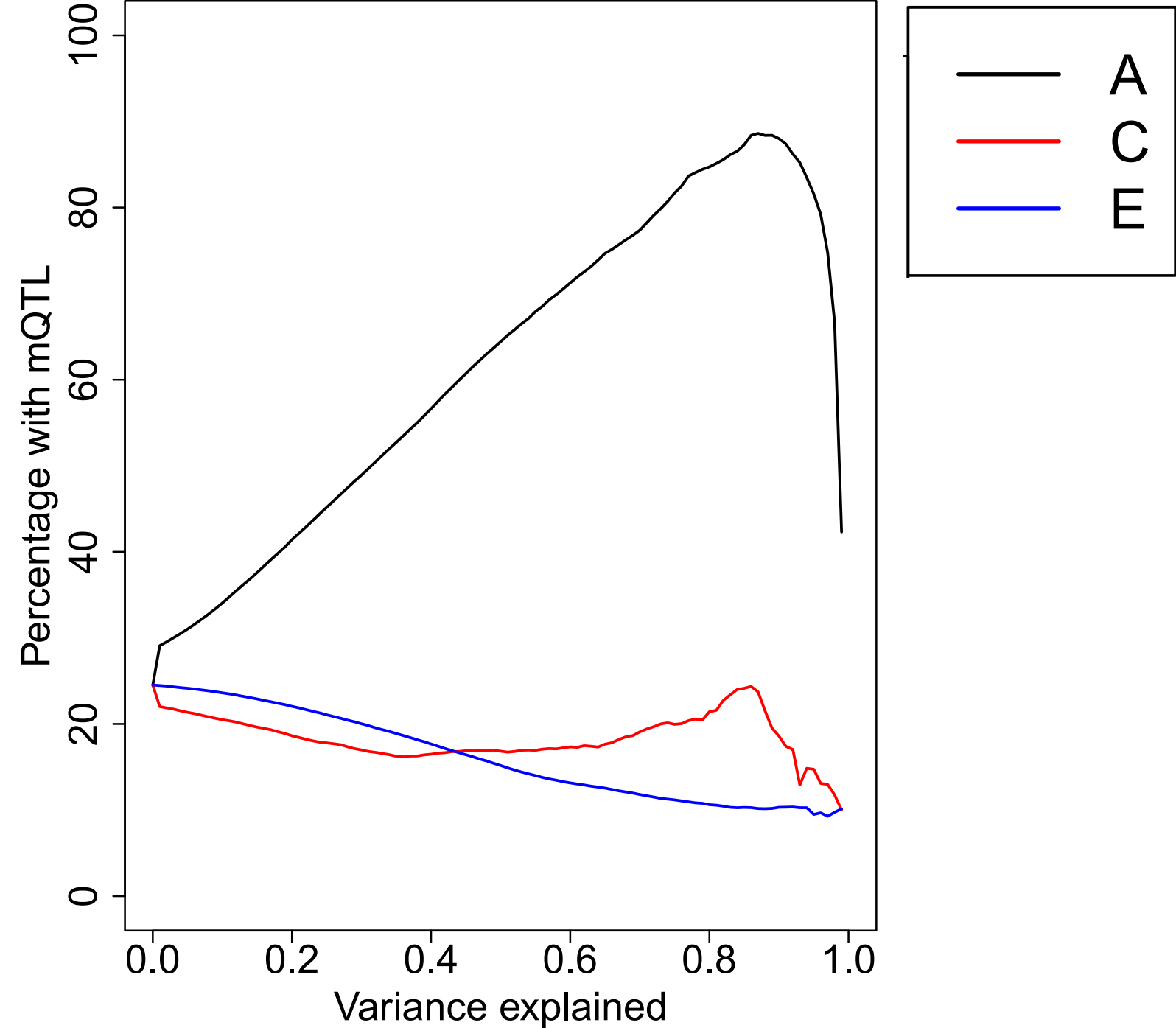

Supplement: S12 Fig — Shown is a line graph of the percentage of DNA methylation sites significantly associated with an mQTL variant in our whole blood dataset[29] (y-axis) as a function of increasing cut-offs for estimates of additive genetic (black line), shared environmental (red line) and non-shared environmental (blue line) effects on DNA methylation (x-axis). (PDF) [file pgen.1007544.s017.pdf]

Cor = 0.5

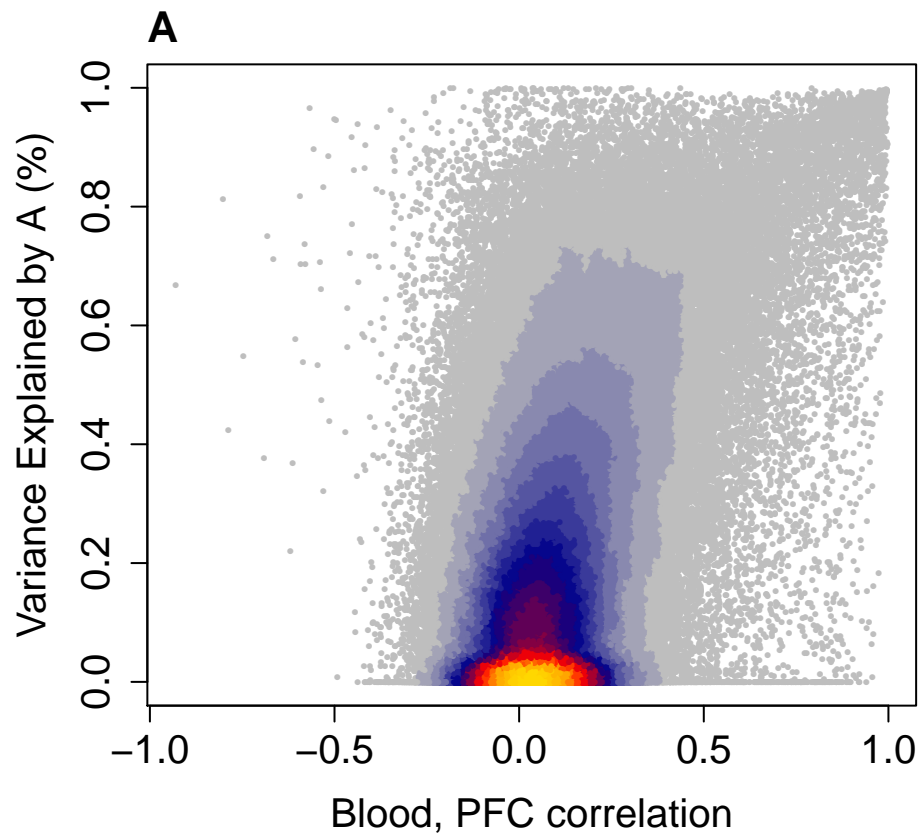

Cor = 0.513

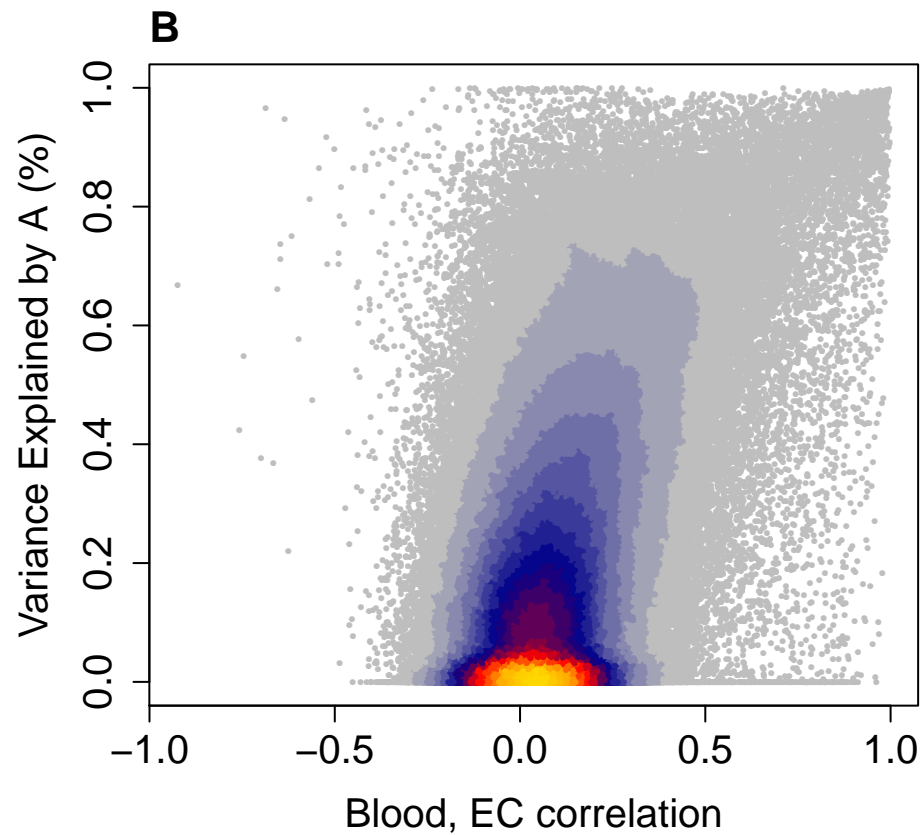

Cor = 0.521

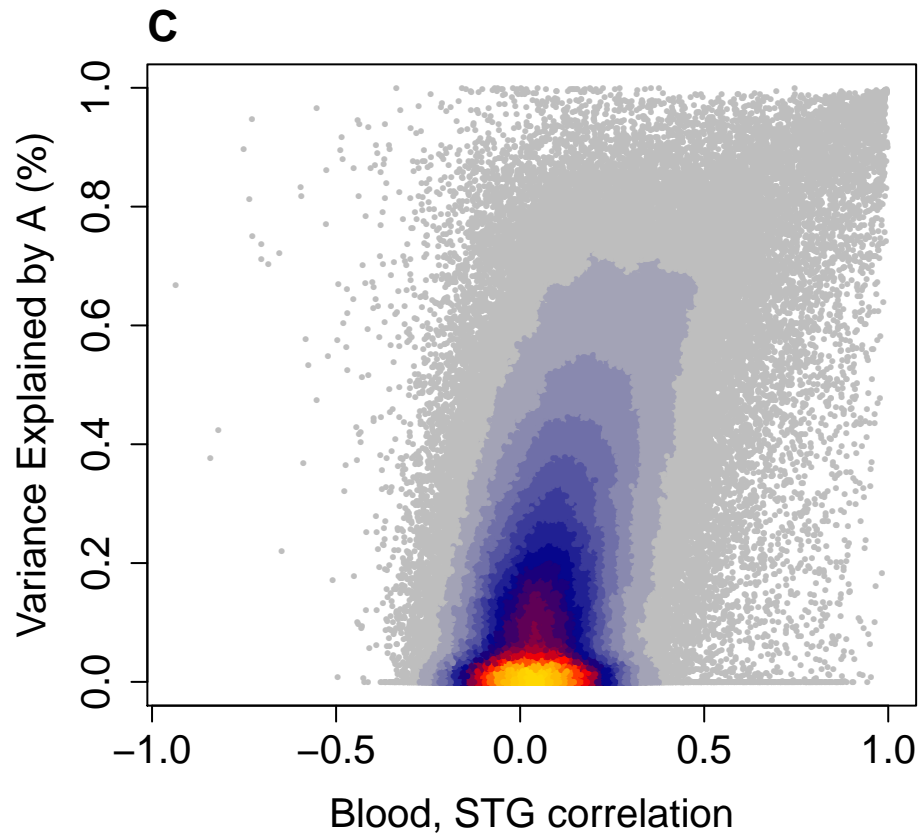

Cor = 0.352

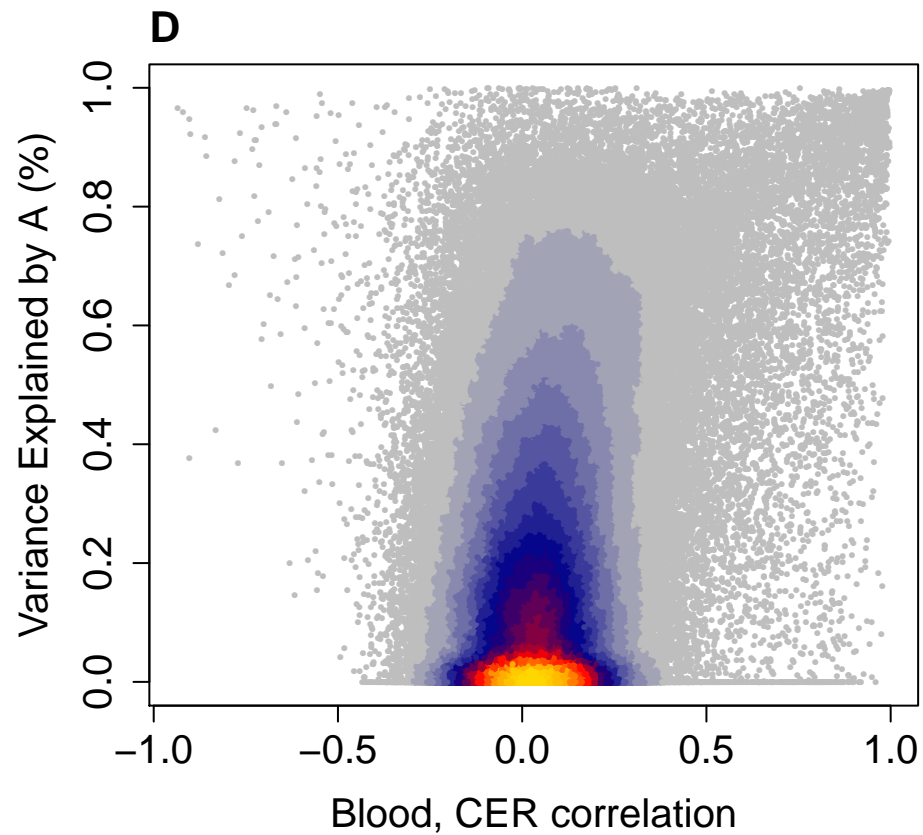

Supplement: S13 Fig — Scatterplot of the amount of variance in DNA methylation explained by additive genetic effects (y-axis) against the level of blood-brain covariation in DNA methylation (x-axis) using data from Hannon et al[30] for all sites on the Illumina 450K array. Shown is data for covariation between whole blood and A) prefrontal cortex, B) entorhinal cortex C) superior temporal gyrus and D) cerebellum. Color indicates the density of points ranging from yellow (high) to gray (low). PFC = prefrontal cortex, EC = entorhinal cortex, STG = superior temporal gyrus, CER = cerebellum. (PDF) [file pgen.1007544.s018.pdf]

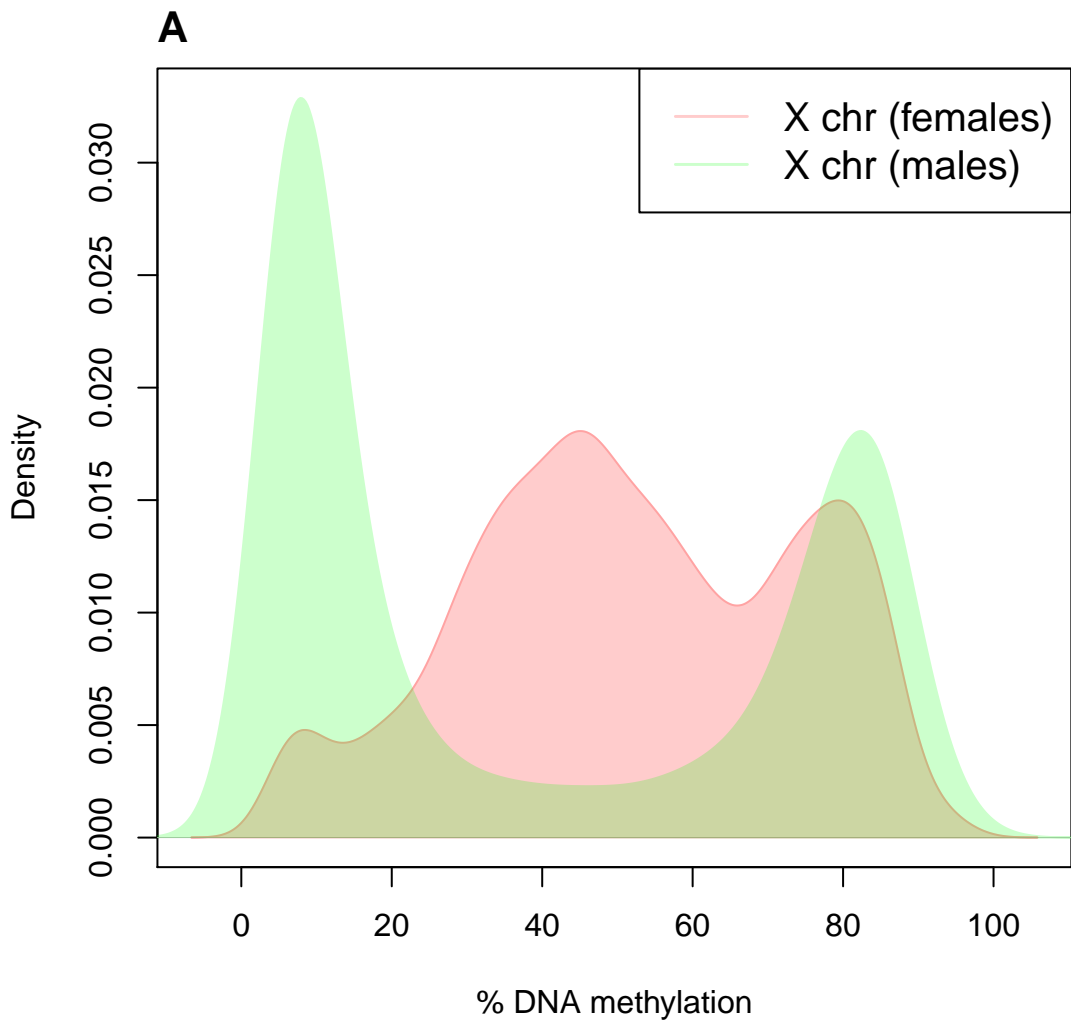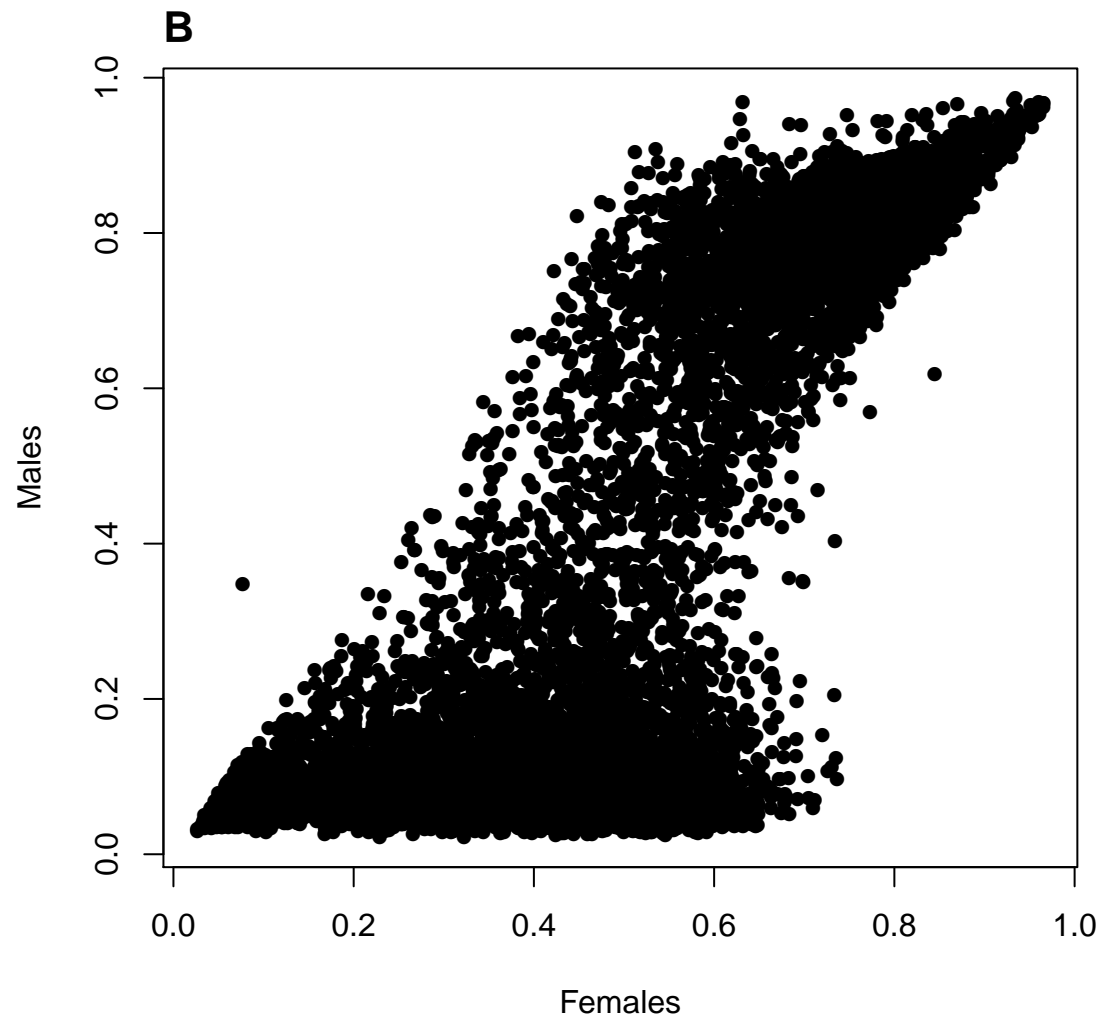

Supplement: S14 Fig — A) Shown is a density plot of DNA methylation across sites on the X chromosome stratified by sex. B) Shown is a scatterplot comparing mean DNA methylation at sites across the X-chromosome in females (x-axis) and males (y-axis). (PDF) [file pgen.1007544.s019.pdf]

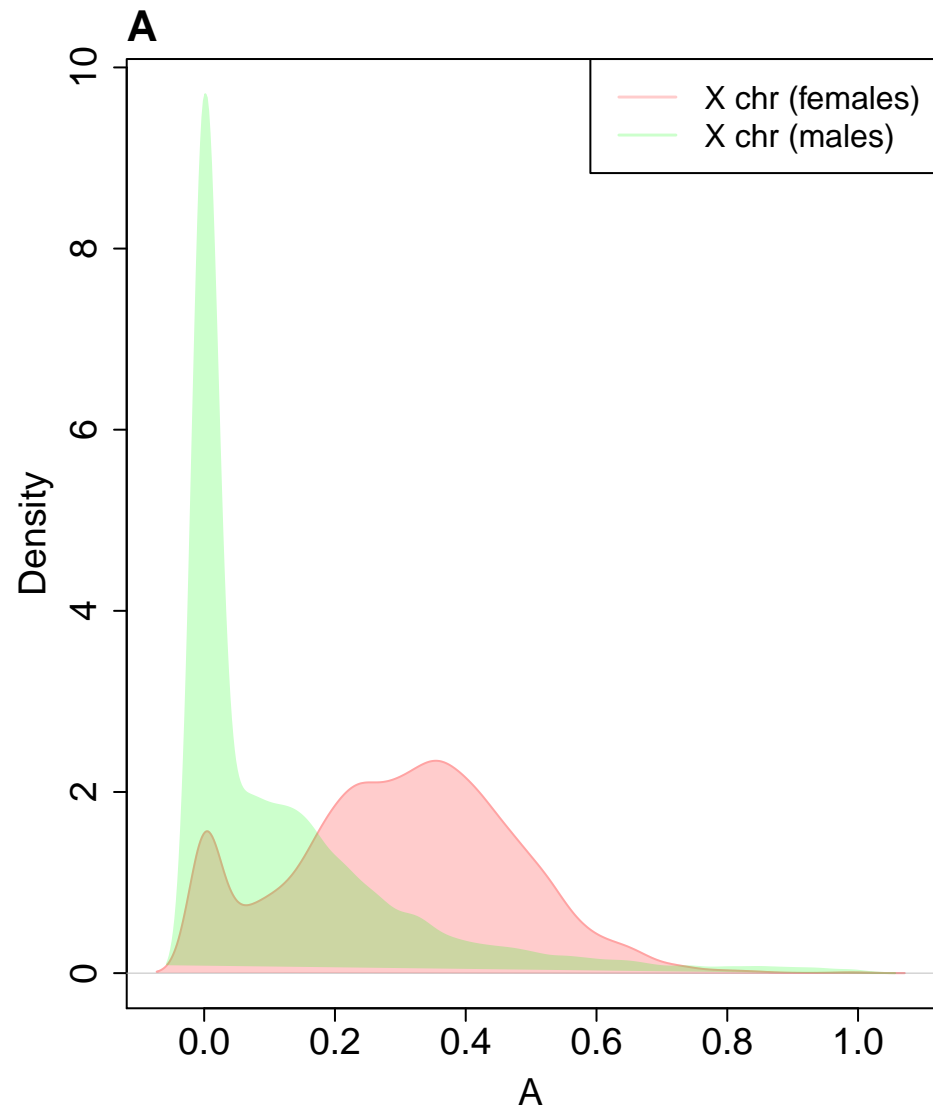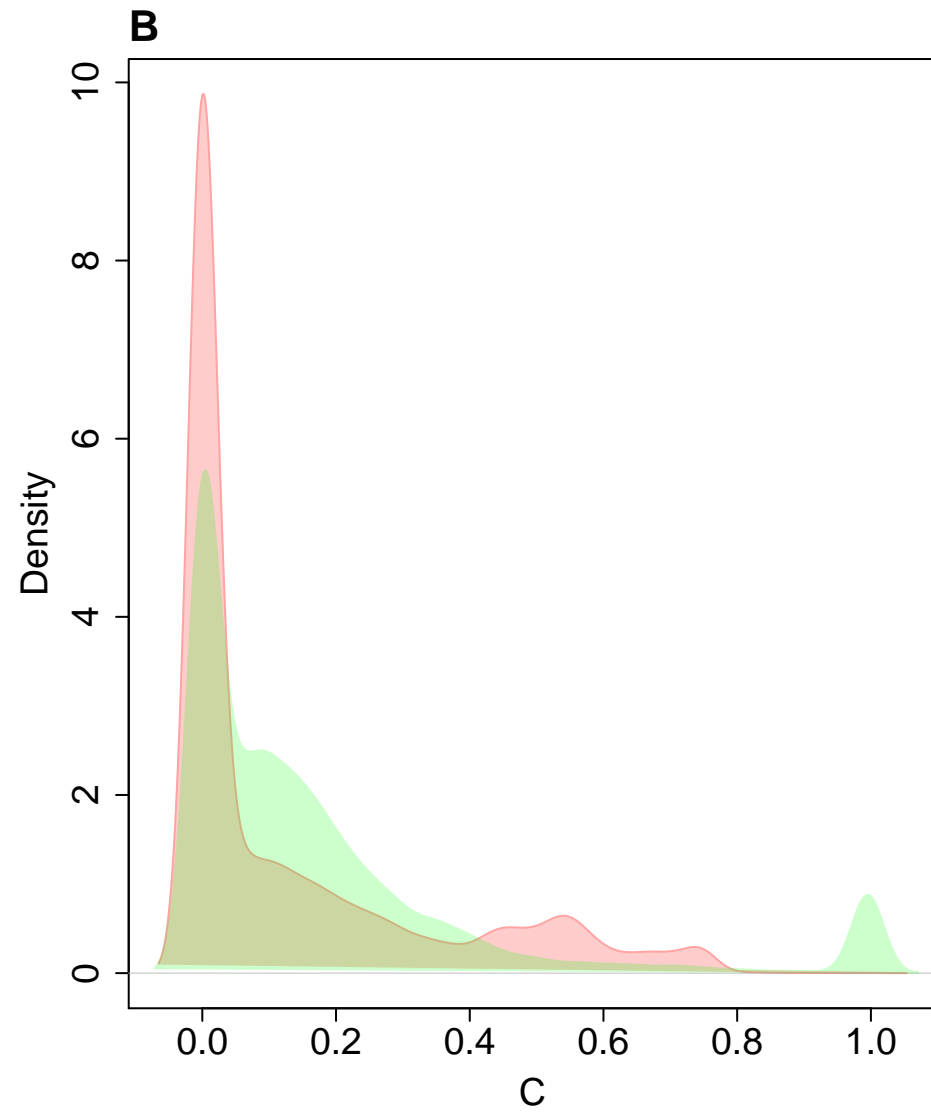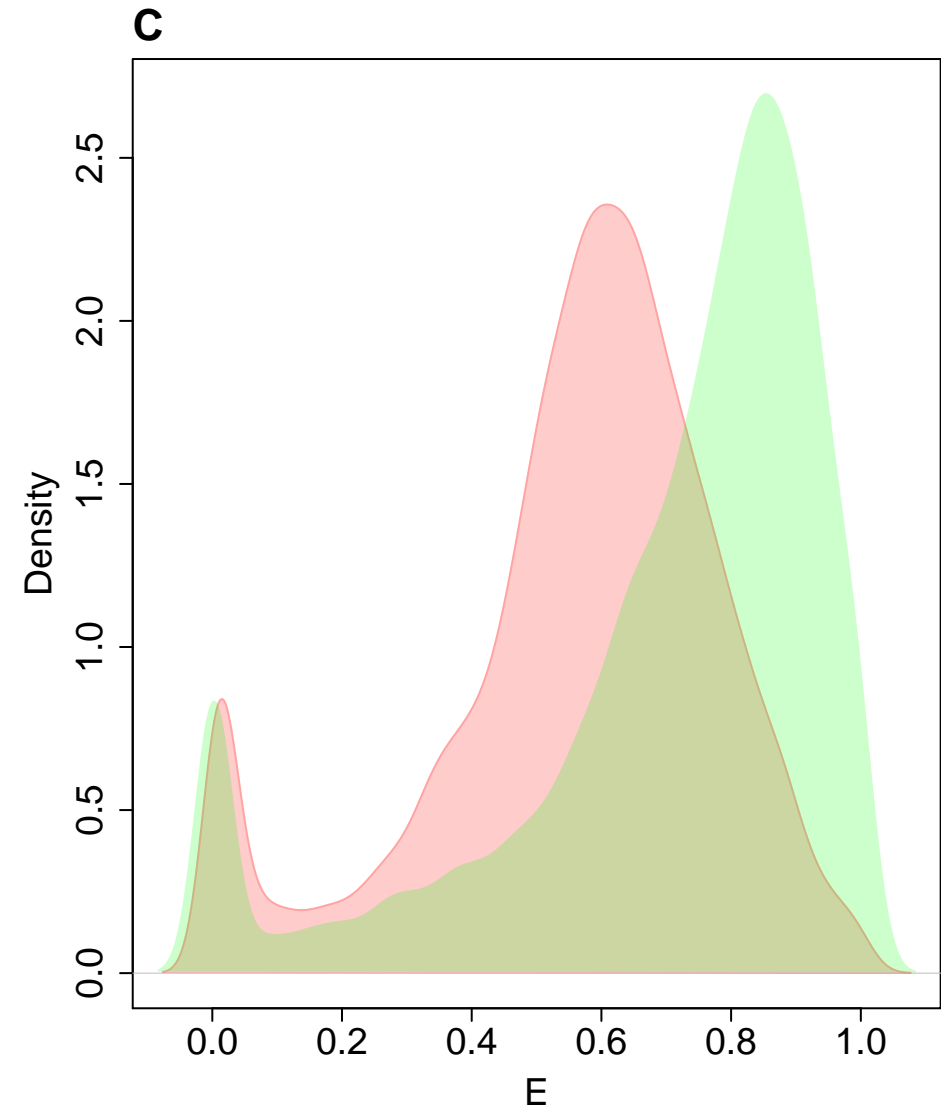

Supplement: S15 Fig — Shown are density plots of estimates of additive genetic effects (A), shared environmental effects (C) and non-shared (or unique) environmental effects (E) stratified by sex (red = females, green = males). (PDF) [file pgen.1007544.s020.pdf]

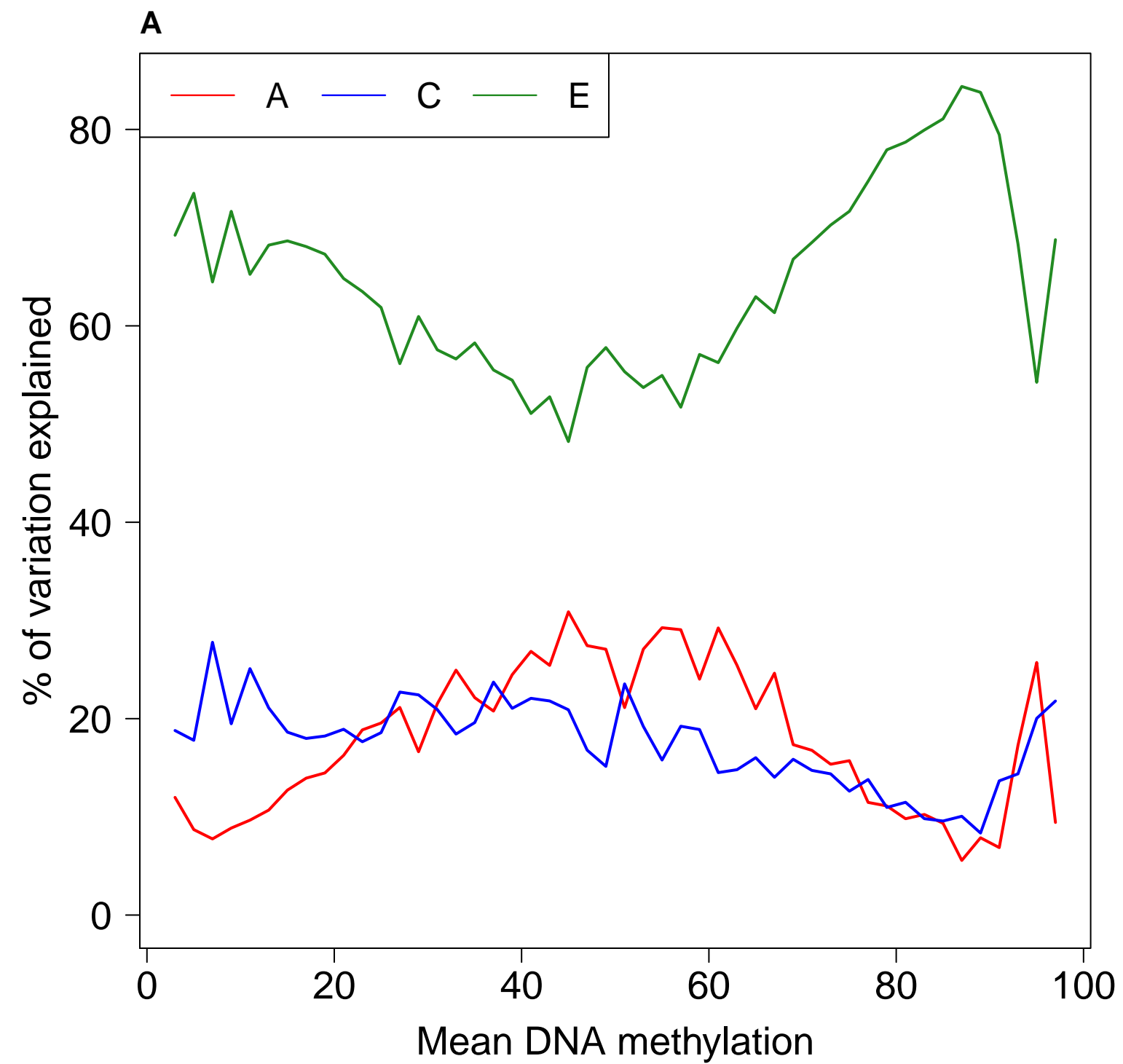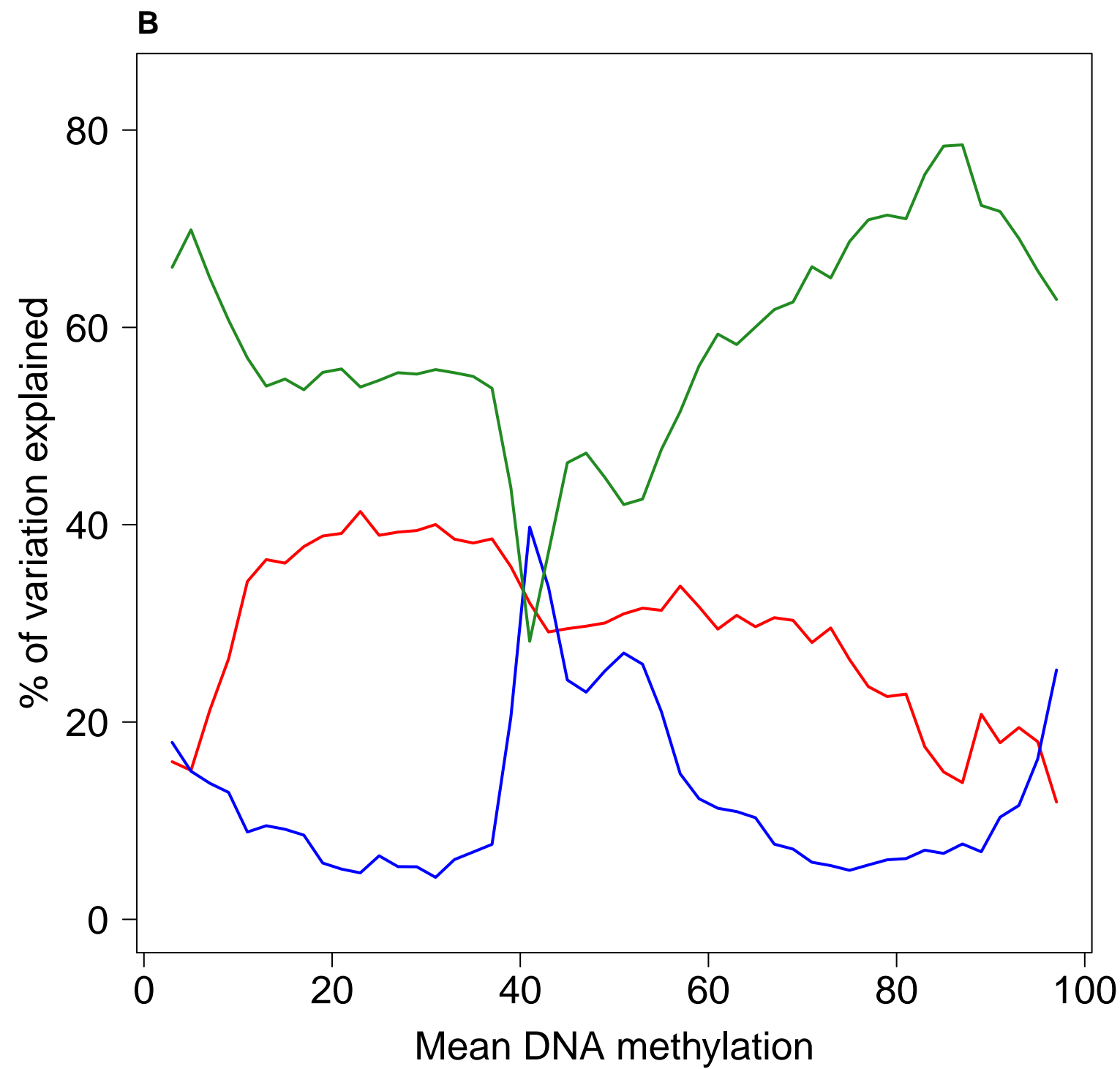

Supplement: S16 Fig — Shown for A) males and B) females are estimates of additive genetic effects (A), shared environmental effects (C) and non-shared (or unique) environmental effects (E) plotted as a function of average DNA methylation level. (PDF) [file pgen.1007544.s021.pdf]

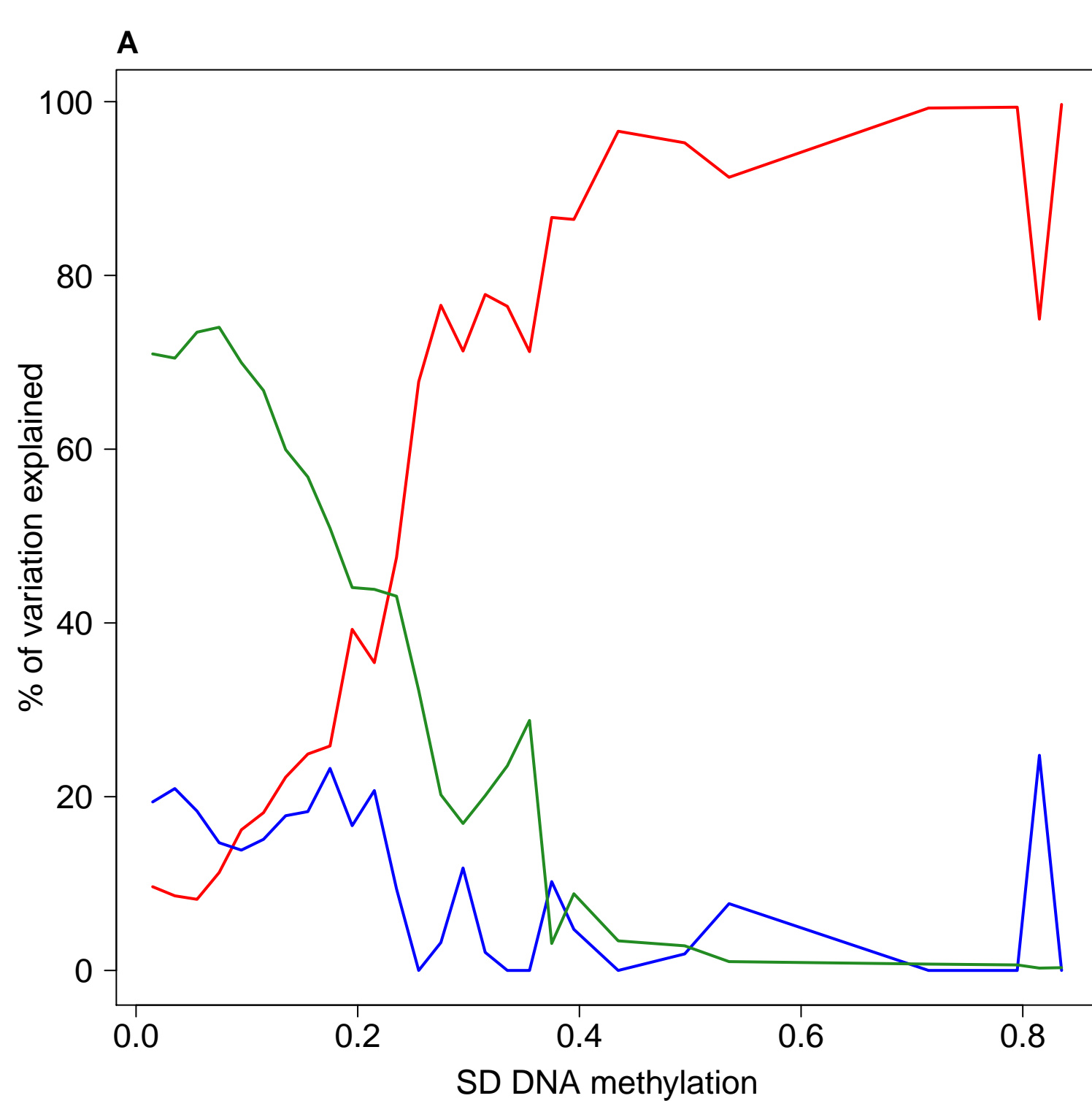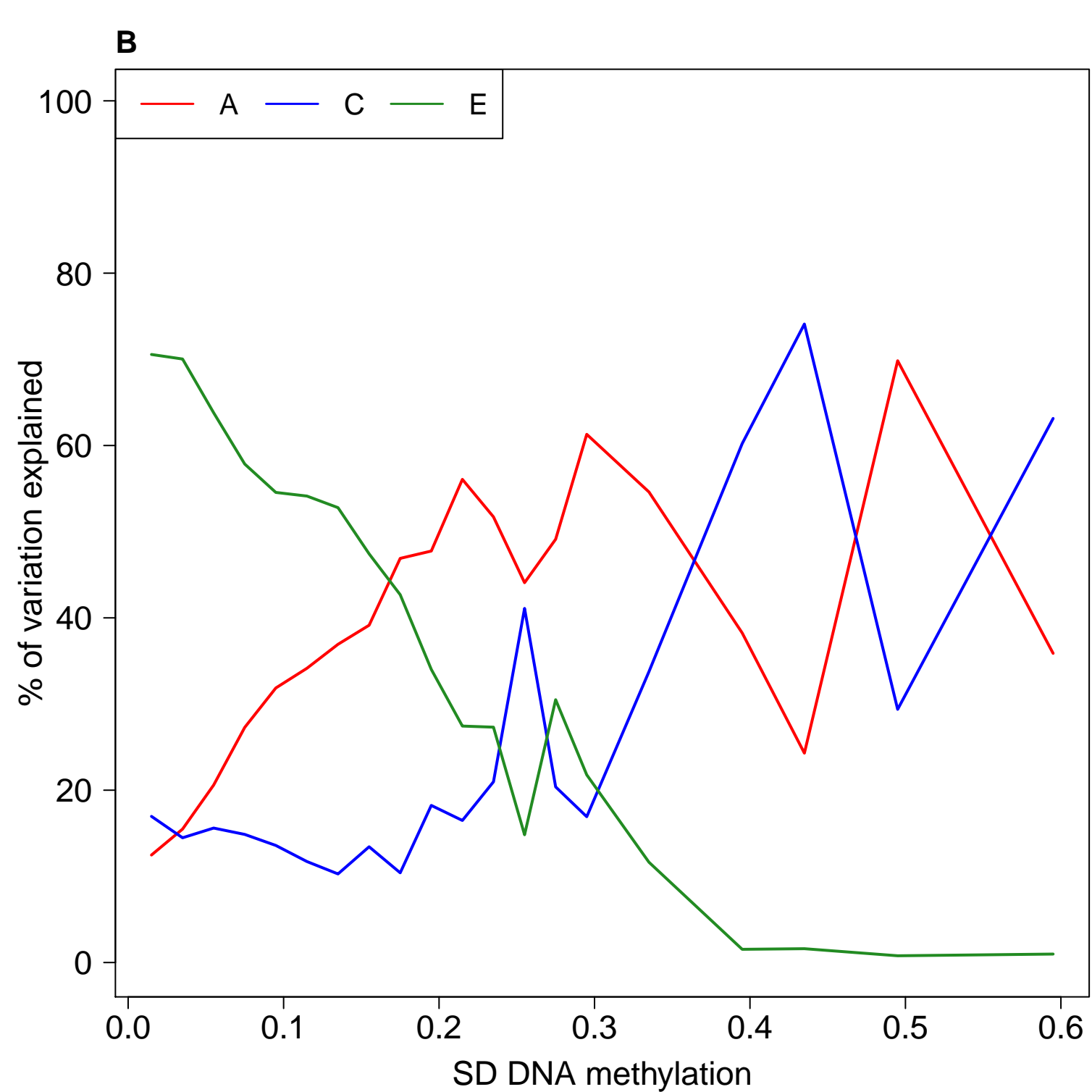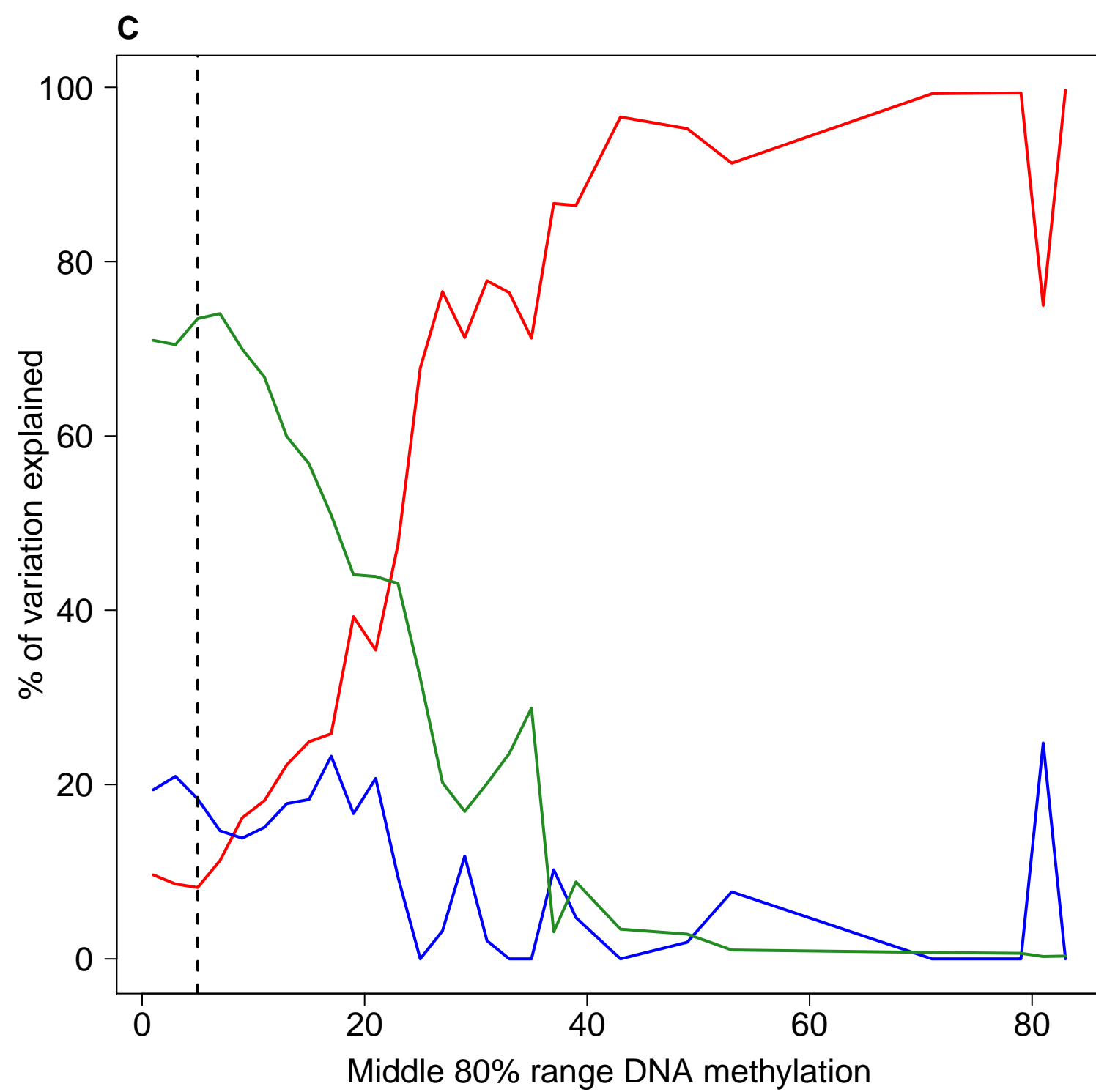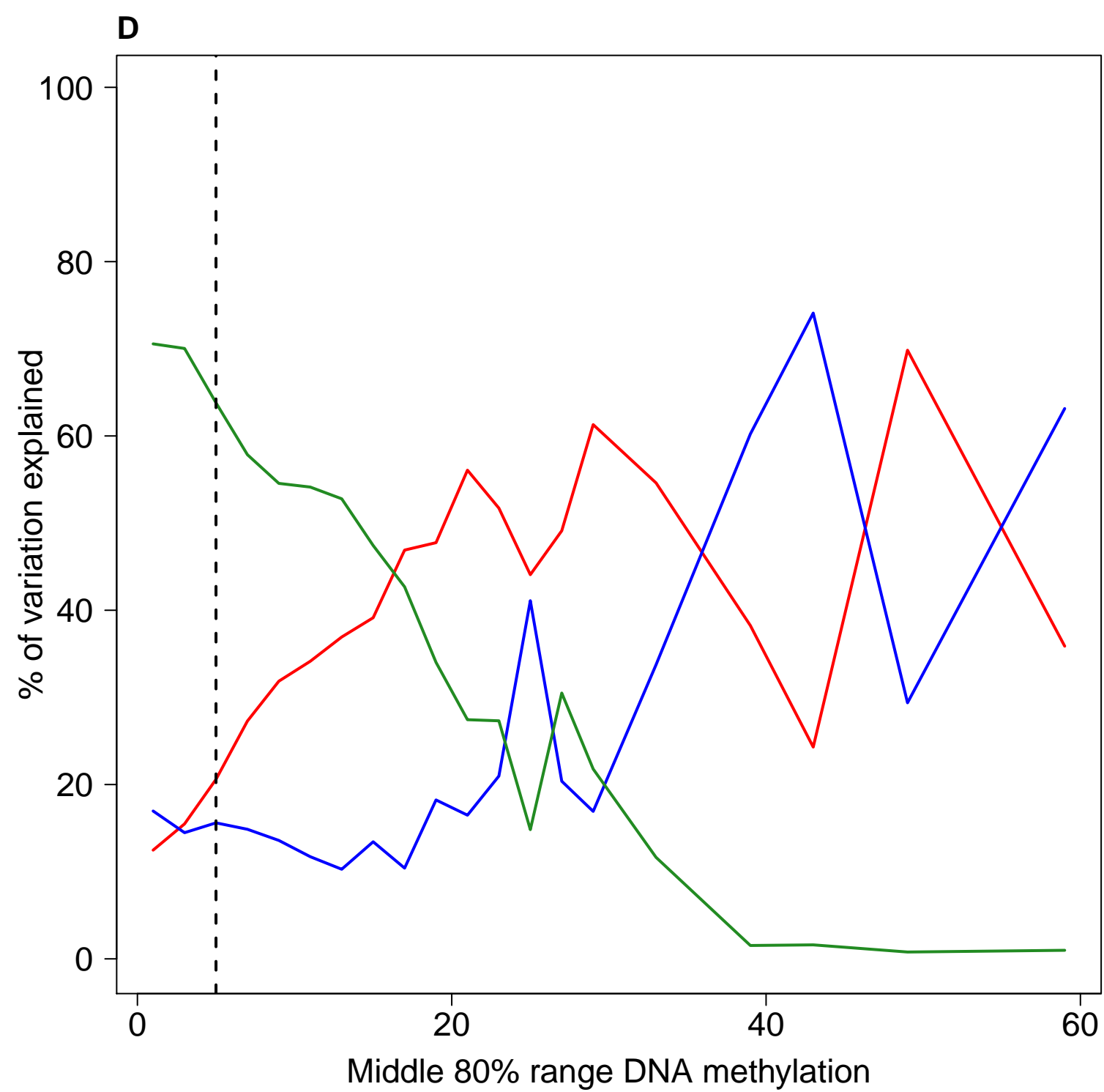

Supplement: S17 Fig — Shown are estimates of additive genetic effects (A), shared environmental effects (C) and unshared (or unique) environmental effects (E) against probe variability. Panels A and B show how genetic and environmental influences differ as a function of the standard deviation (SD) in DNA methylation in males and females, respectively. Panels C and D show how genetic and environmental influences differ as a function of the middle 80% of the distribution of DNA methylation levels in males and females, respectively. The dashed vertical line indicates the cut-off of 5% used to define probes as being “variable”. (PDF) [file pgen.1007544.s022.pdf]

Males

$r = 0.258$

**A**

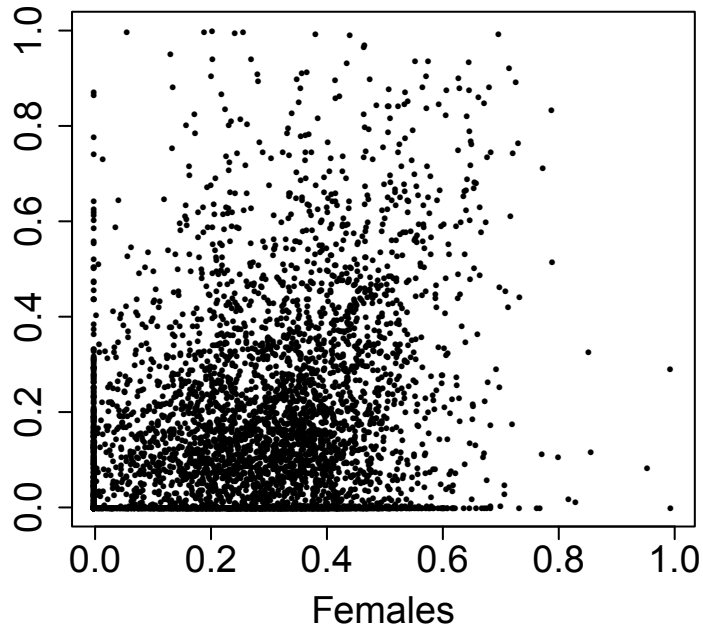

Males

$r = 0.185$

**B**

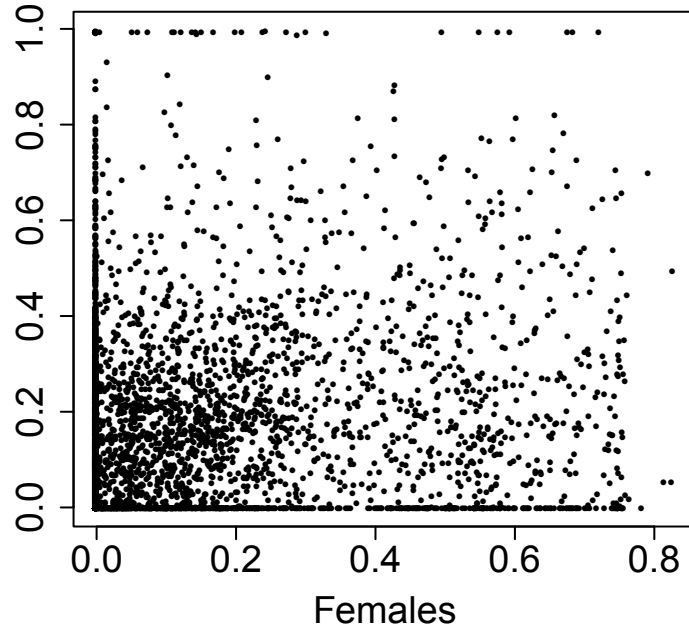

Males

$r = 0.381$

**C**

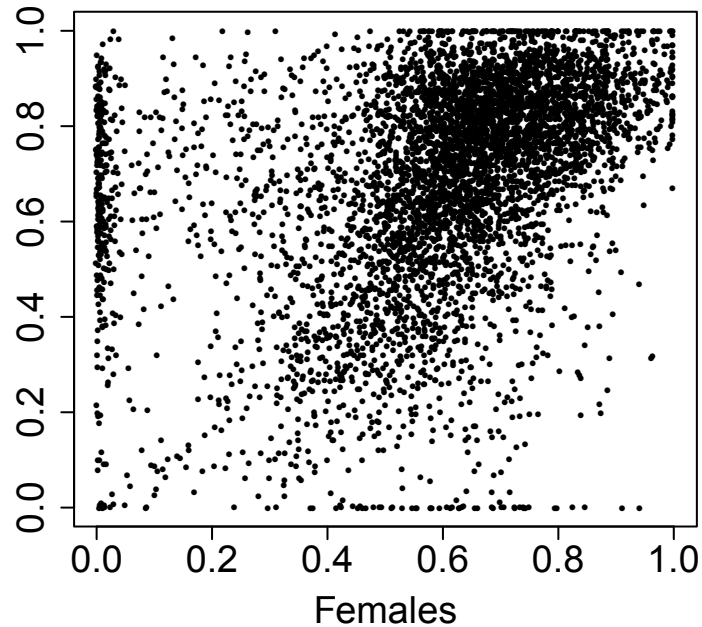

Supplement: S18 Fig — Shown are scatterplots of the A) additive genetic, B) shared environmental, and C) non-shared environmental contribution to DNA methylation for sites on the X chromosome in female (x-axis) and male (y-axis) twin pairs. (PDF) [file pgen.1007544.s023.pdf]

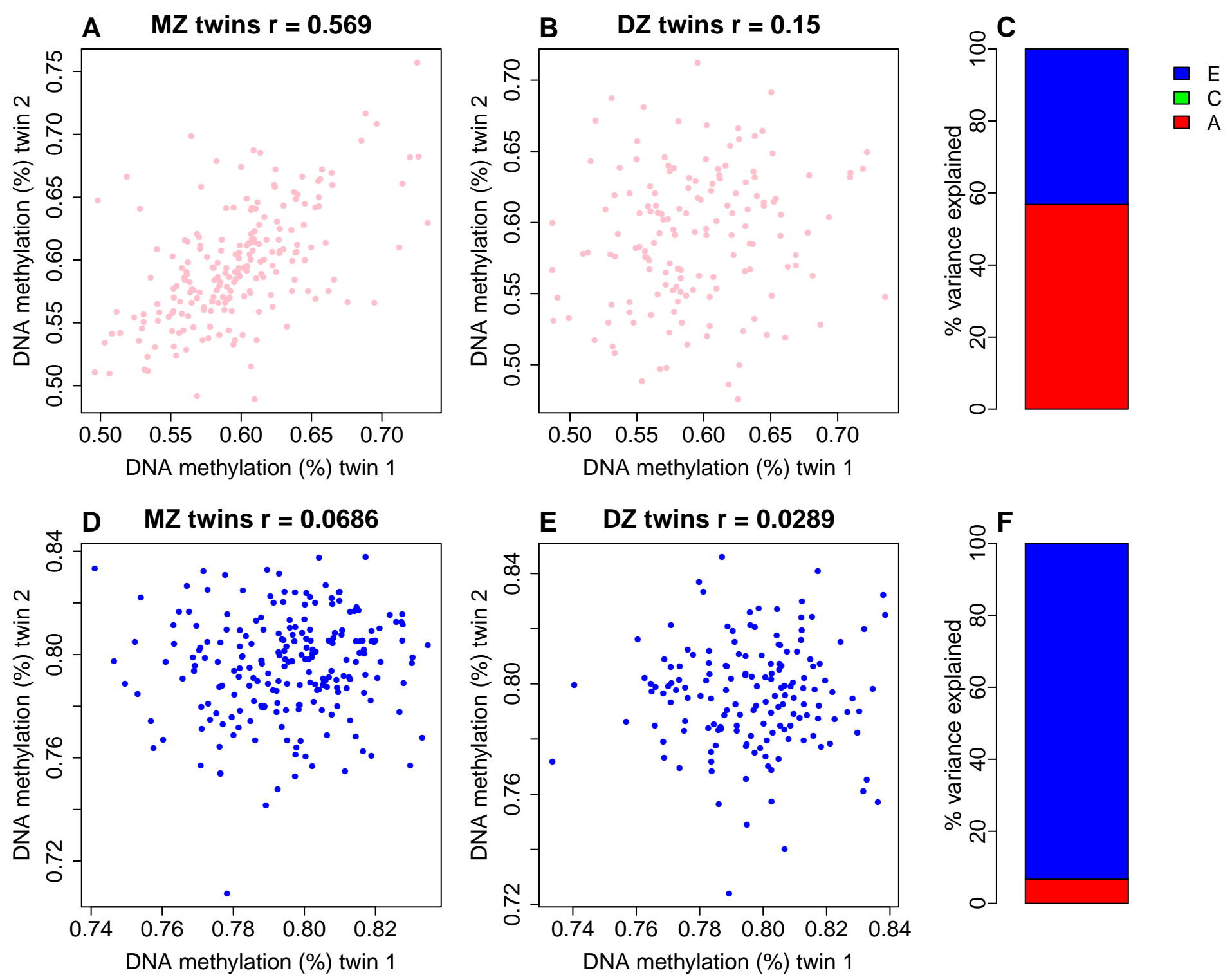

Supplement: S19 Fig — The scatterplots show DNA methylation values in A) female MZ, B) female DZ, D) male MZ, and E) male DZ twin pairs. Each point represents an individual twin-pair. At this site, the twin correlation of DNA methylation is notably higher in female MZ twins (r = 0.569) compared to female DZ twins (r = 0.15), whereas the correlations for male MZ twins (r = 0.0686) and male DZ twins (r = 0.0289) are similar. Results from structural equation modelling are presented as stacked bar-plots for C) female and F) male twin-pairs respectively, highlighting higher genetic effects on DNA methylation at this site in females than males. (PDF) [file pgen.1007544.s024.pdf]

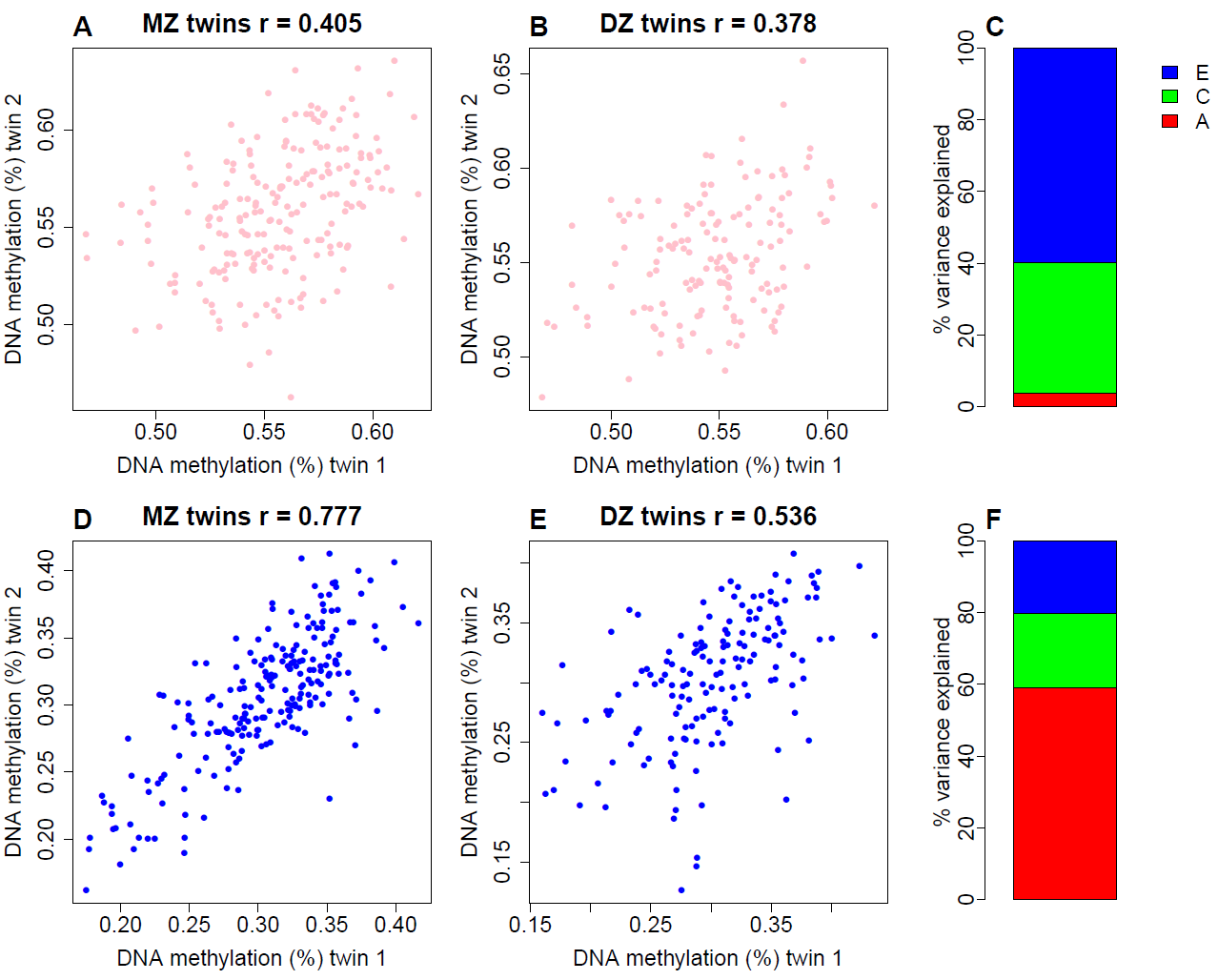

Supplement: S20 Fig — The scatterplots show DNA methylation values in A) female MZ, B) female DZ, D) male MZ, and E) male DZ twin pairs. Each point represents an individual twin-pair. At this site, the correlation of DNA methylation is notably higher in male MZ twins (r = 0.777) compared to female DZ twins (r = 0.536), whereas the correlations for male MZ twins (r = 0.405) and male DZ twins (r = 0.378) are similar. Results from structural equation modelling are presented as stacked bar-plots for C) female and F) male twin-pairs respectively, highlighting higher heritability in females than males. (TIF) [file pgen.1007544.s025.tif]

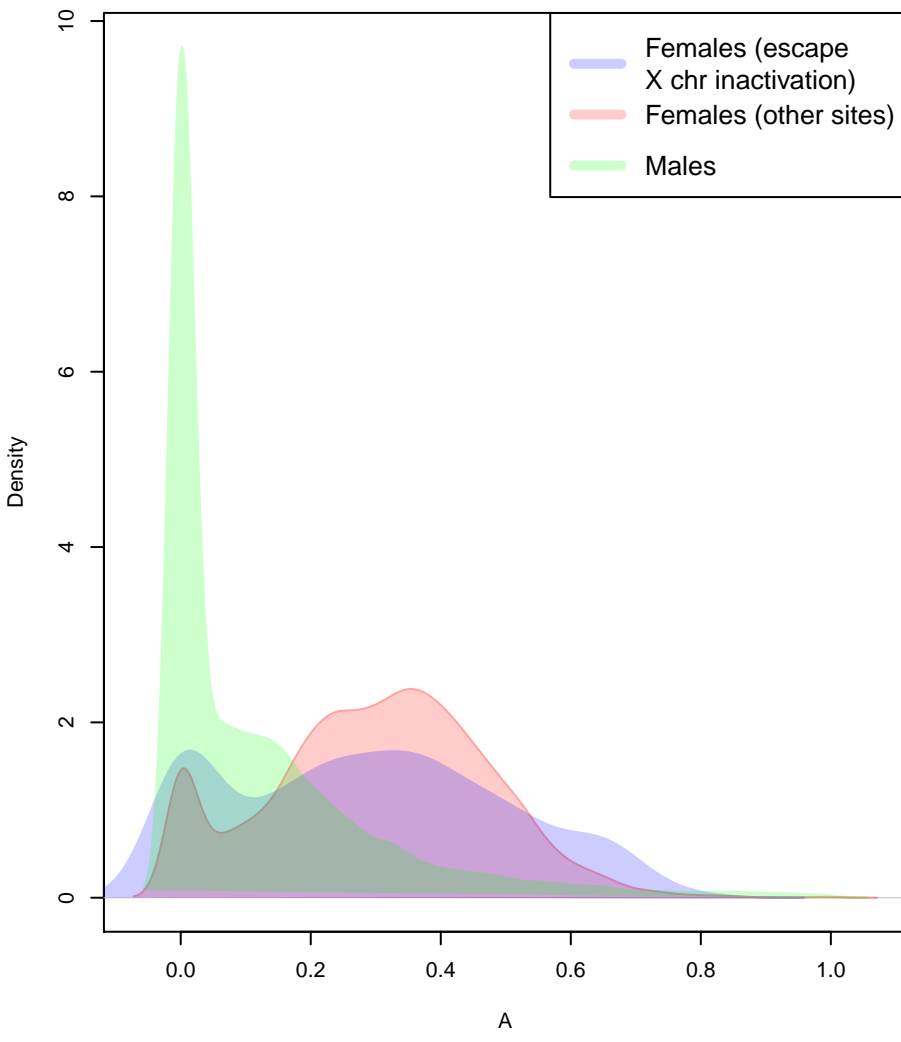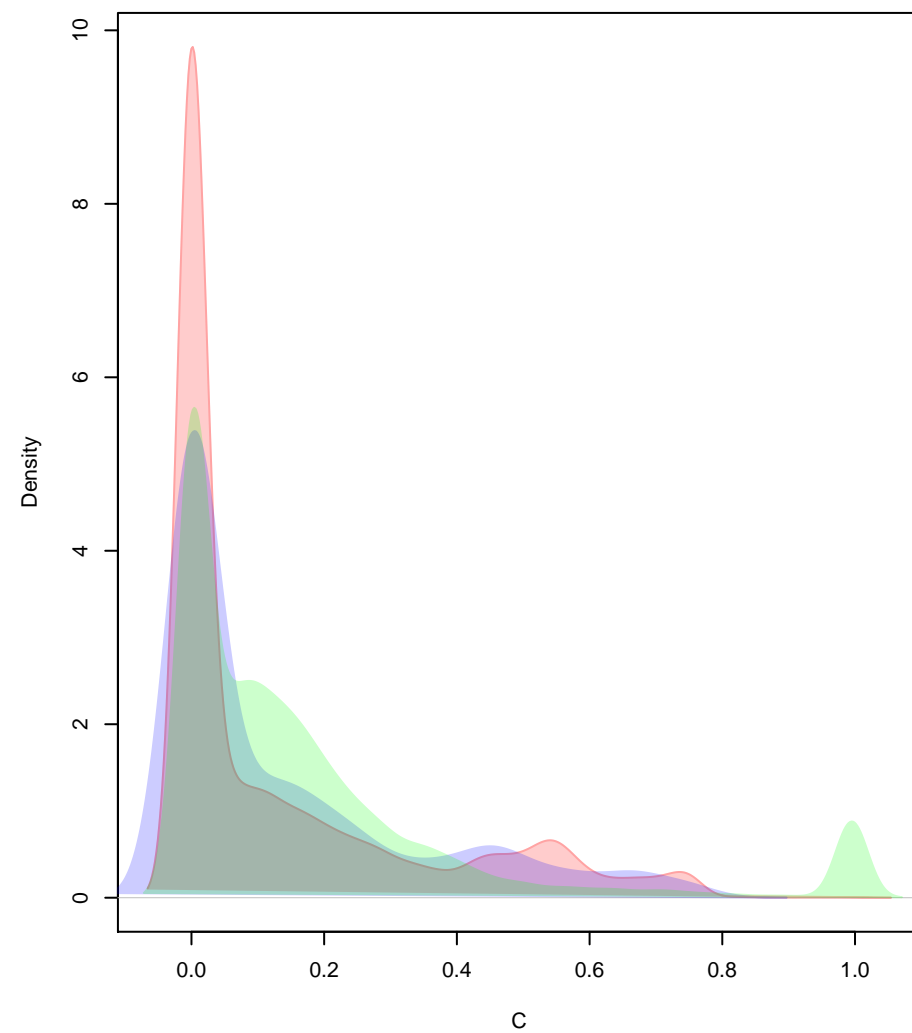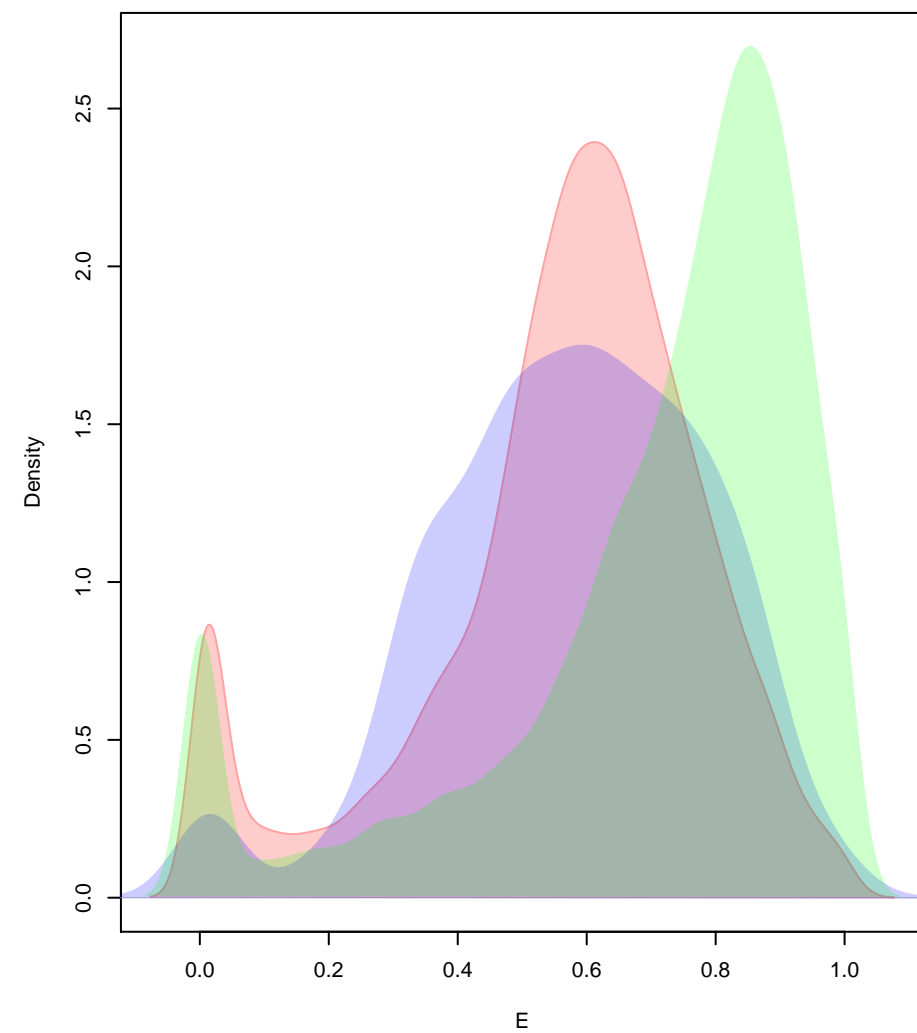

Supplement: S21 Fig — Shown are density plots of estimates of additive genetic effects (A), shared environmental effects (C) and non-shared (or unique) environmental effects (E) stratified by sex and within females stratified by sites located in the transcription start site or 5’UTR of genes that escape XCI (red = females, blue = females sites that escape X chromosome inactivation, green = males). (PDF) [file pgen.1007544.s026.pdf]

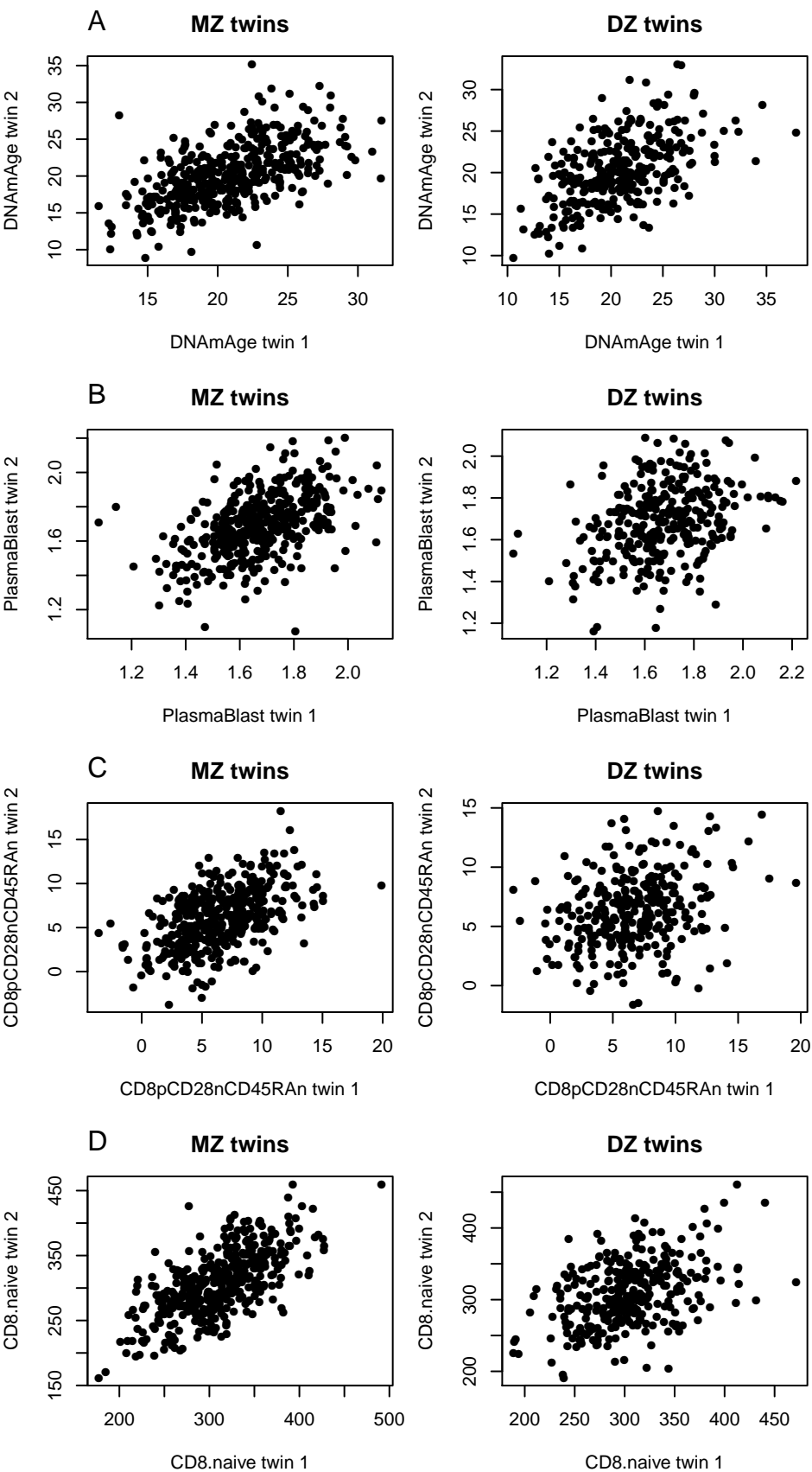

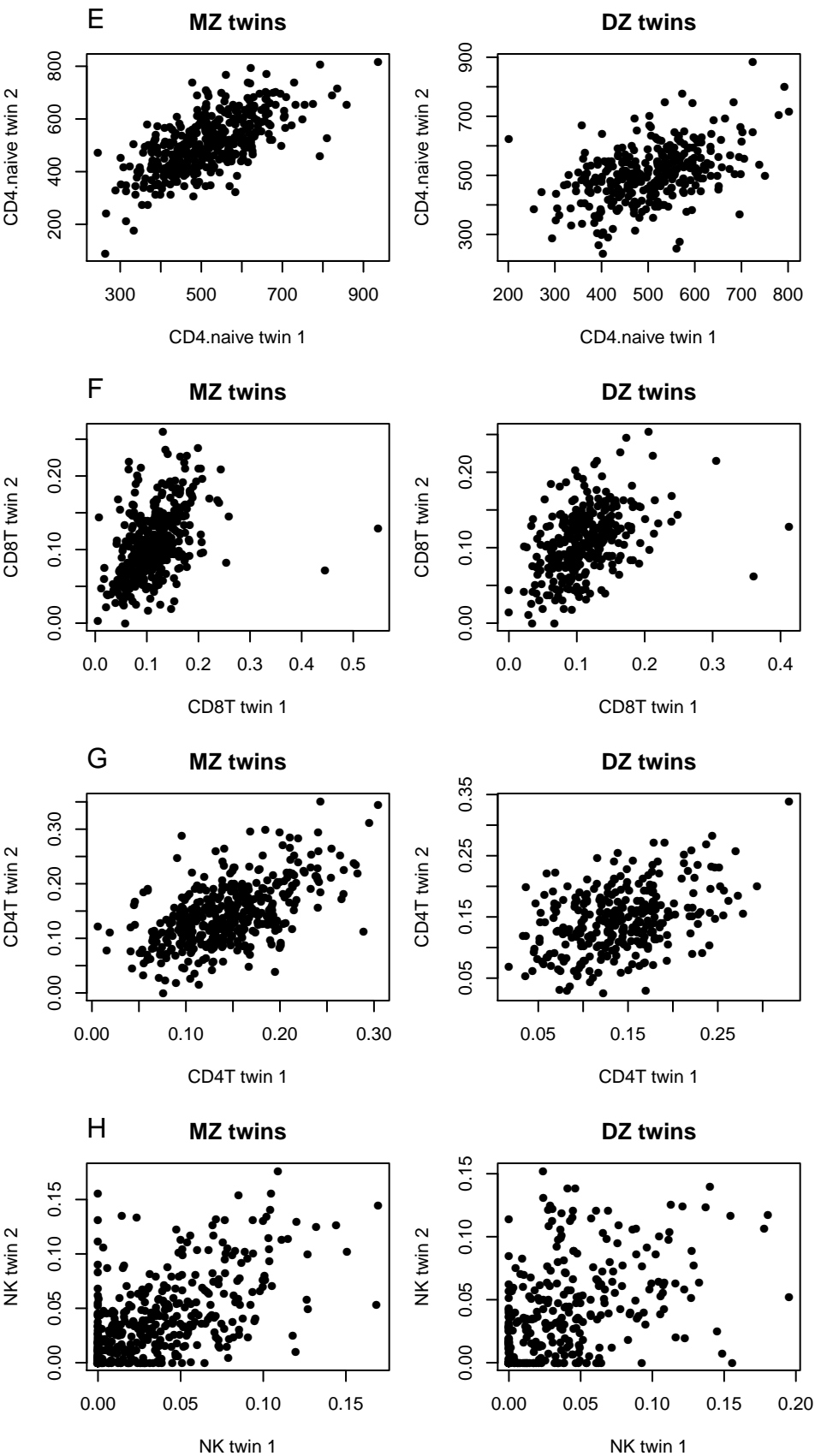

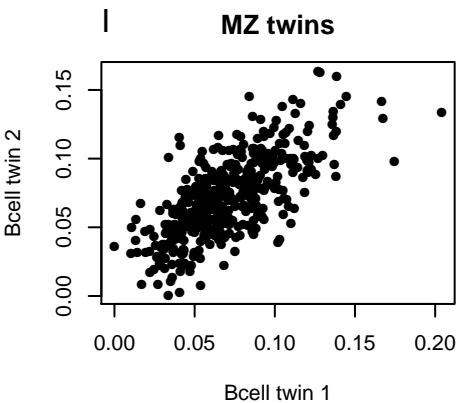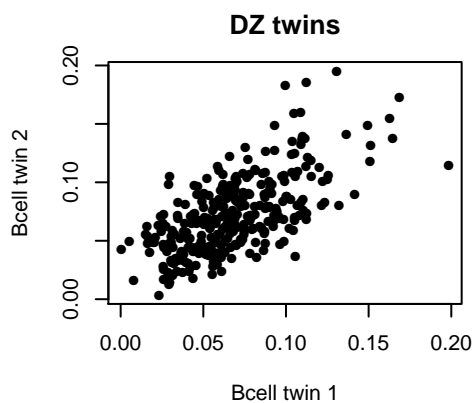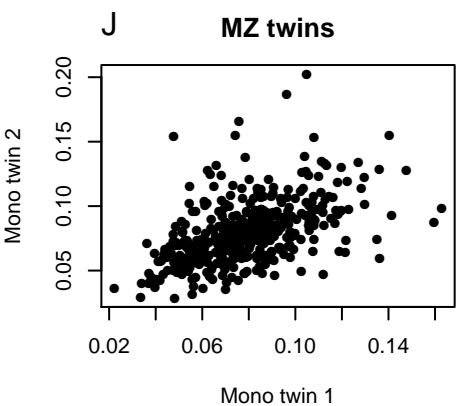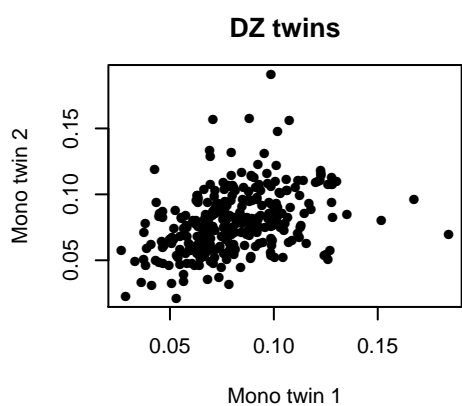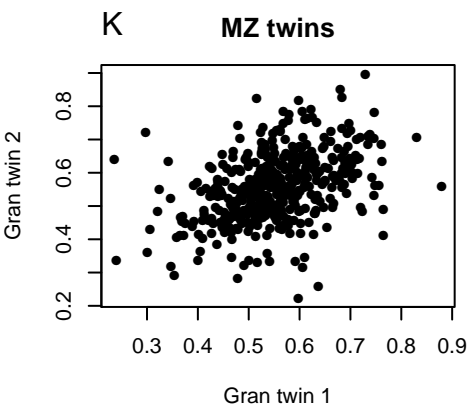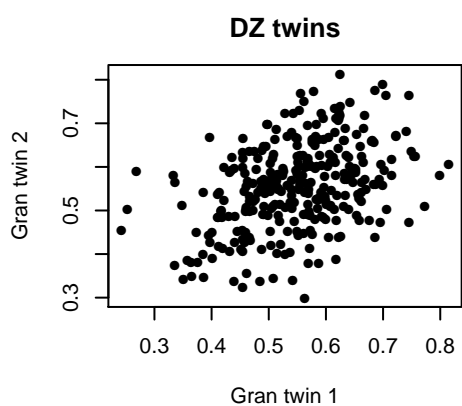

Supplement: S22 Fig — Shown are co-twin correlations for A) DNA methylation age, B) estimated plasma blast abundance, C) estimated CD8+CD28-CD45RA- T cell abundance, D) estimated naïve CD8 T cell abundance, E) estimated naive CD4 T cell abundance (all derived using the online Epigenetic Clock software[32]), F) estimated CD8 T cell proportion, G) estimated CD4 T cell proportion, H) estimated natural killer cell proportion, I) estimated B cell proportion, J) estimated monocyte proportion, and K) estimated granulocyte proportion (all derived using the Houseman algorithm[33, 34]). Panels on the left show correlations for monozygotic (MZ) twin pairs and panels on the right show correlations for dizygotic (DZ) twin pairs. (PDF) [file pgen.1007544.s027.pdf]

ACE heritability of Estimated Cell Counts

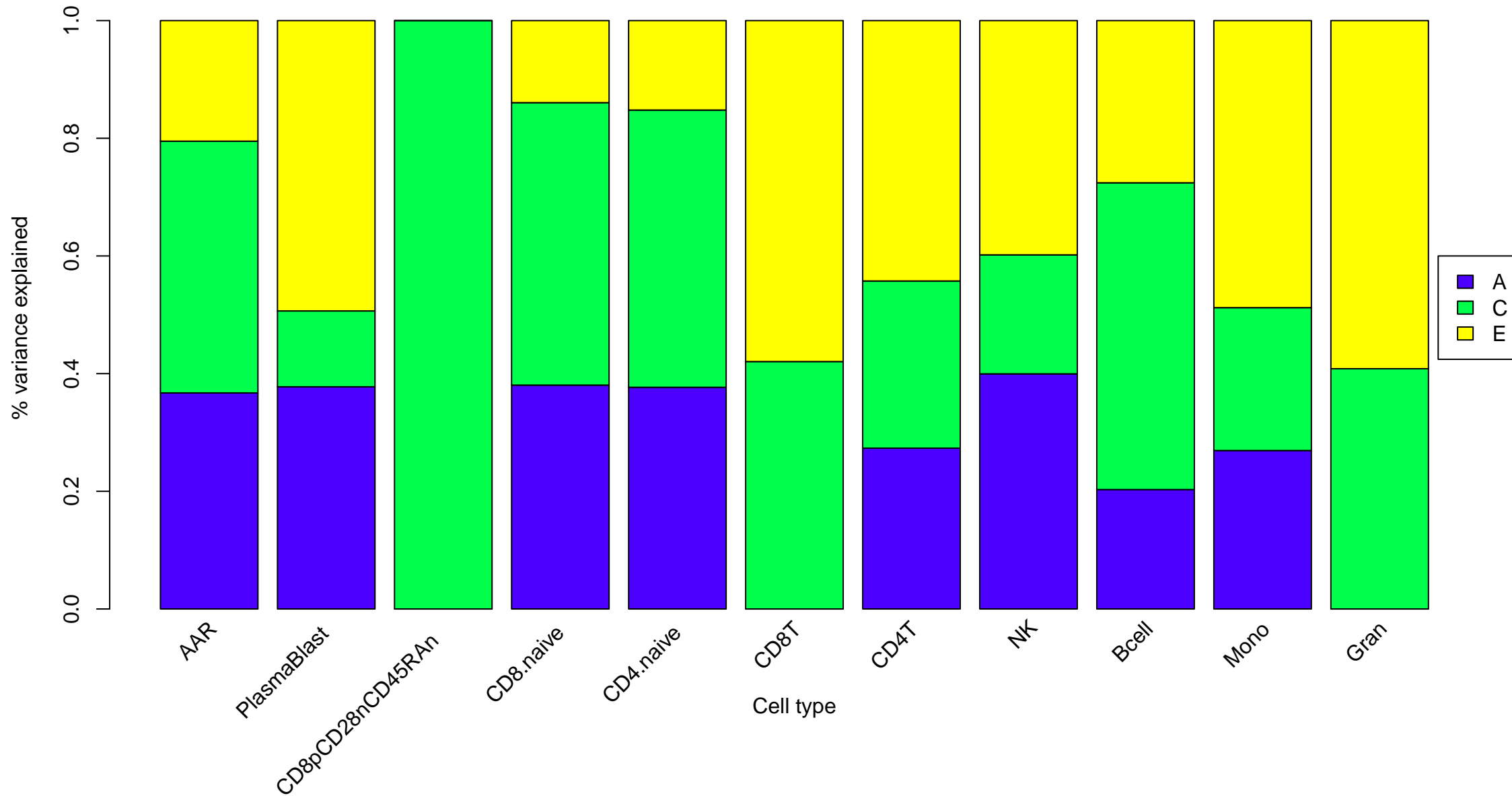

Supplement: S23 Fig — AAR = age acceleration residual derived from the DNA methylation age clock. (PDF) [file pgen.1007544.s028.pdf]

**A**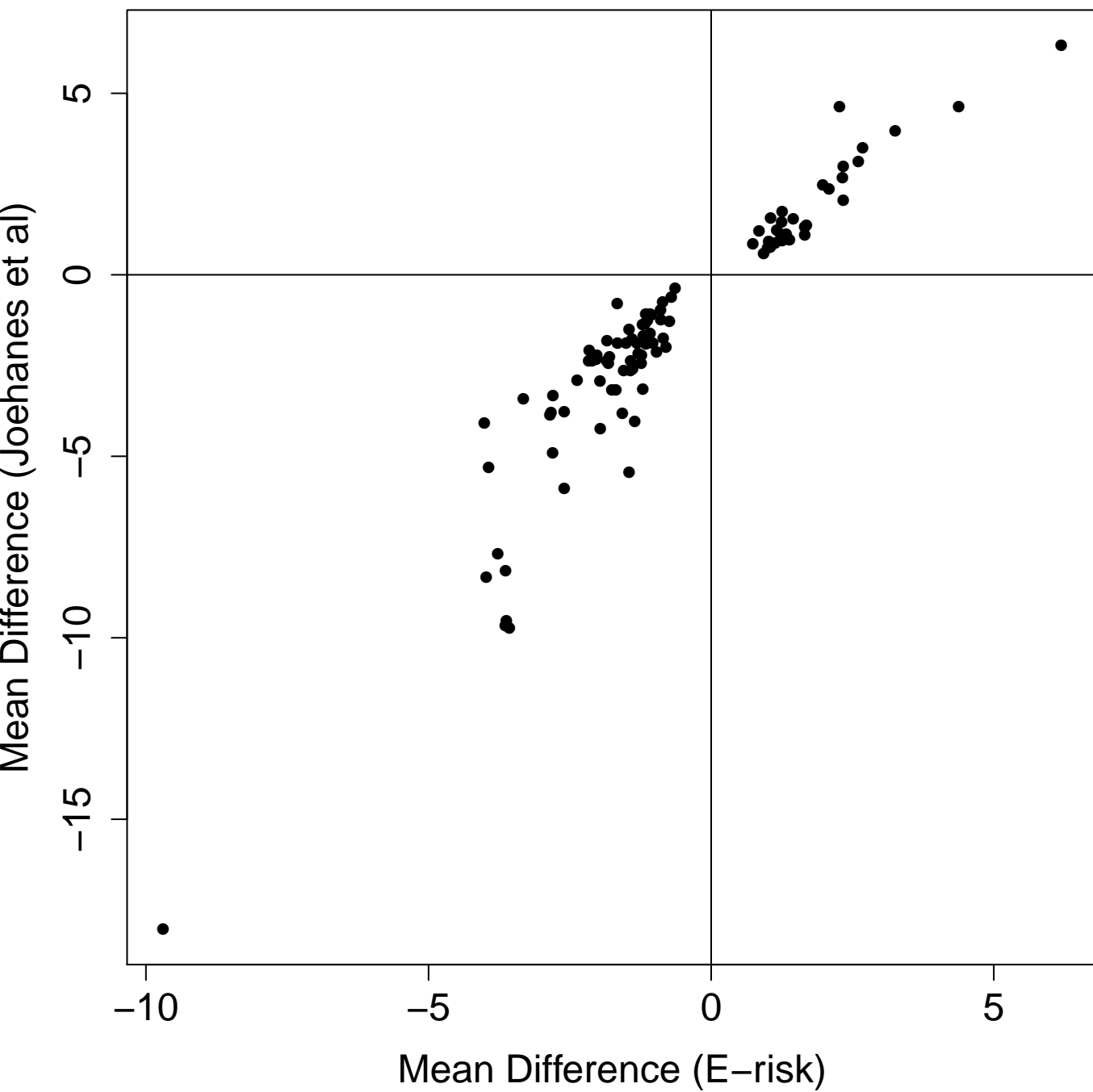**B**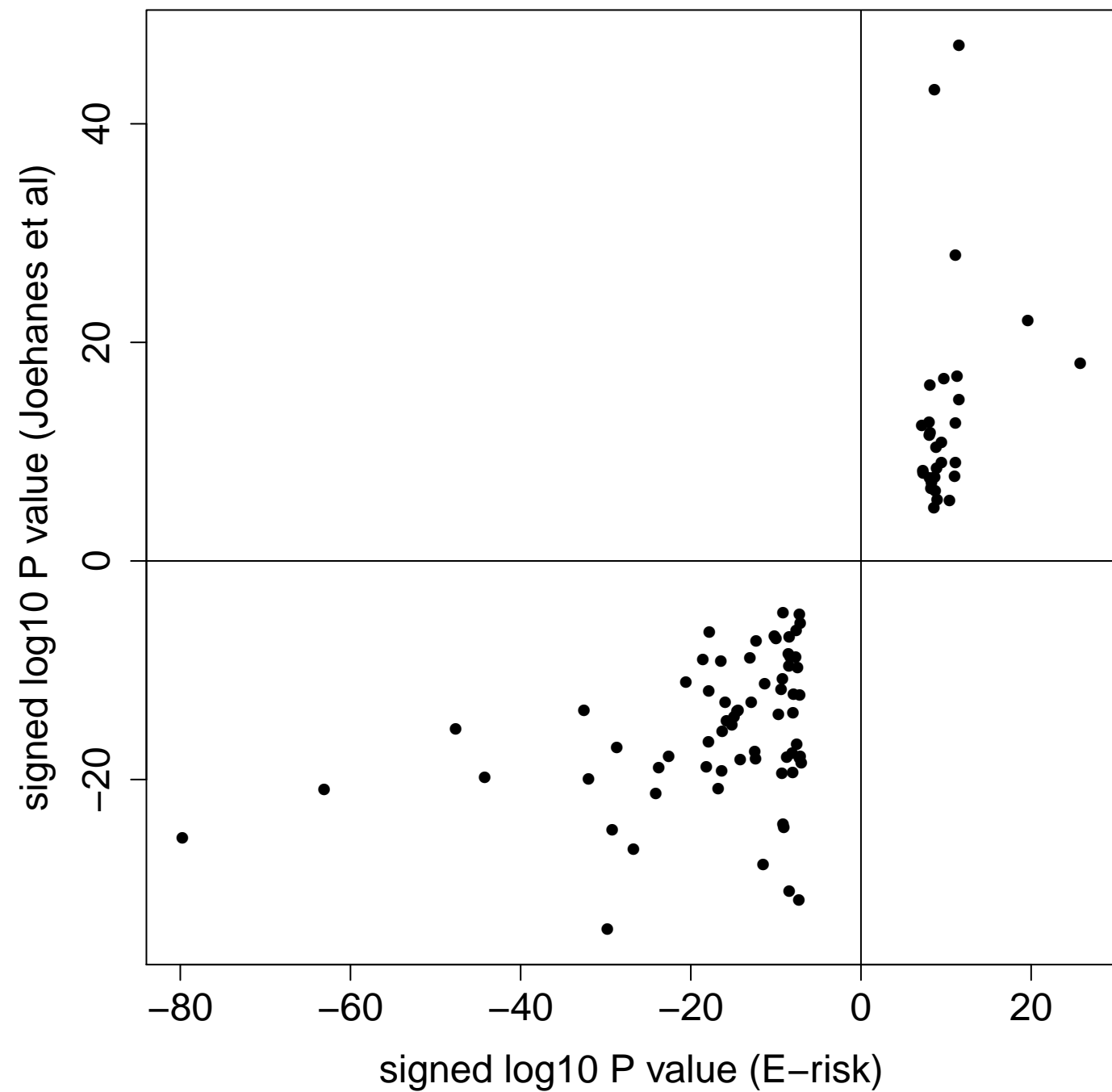

Supplement: S24 Fig — A) The mean difference between current smokers and never smokers from the E-risk cohort (x-axis) against a similar study in adults taken from Joehanes et al[44] (y-axis). B) Shown is the correlation of the signed log10 P-values from a comparison between current smokers and never smokers from the E-risk cohort (x-axis) against a similar study in adults taken from Joehanes et al[44] (y-axis). (PDF) [file pgen.1007544.s029.pdf]

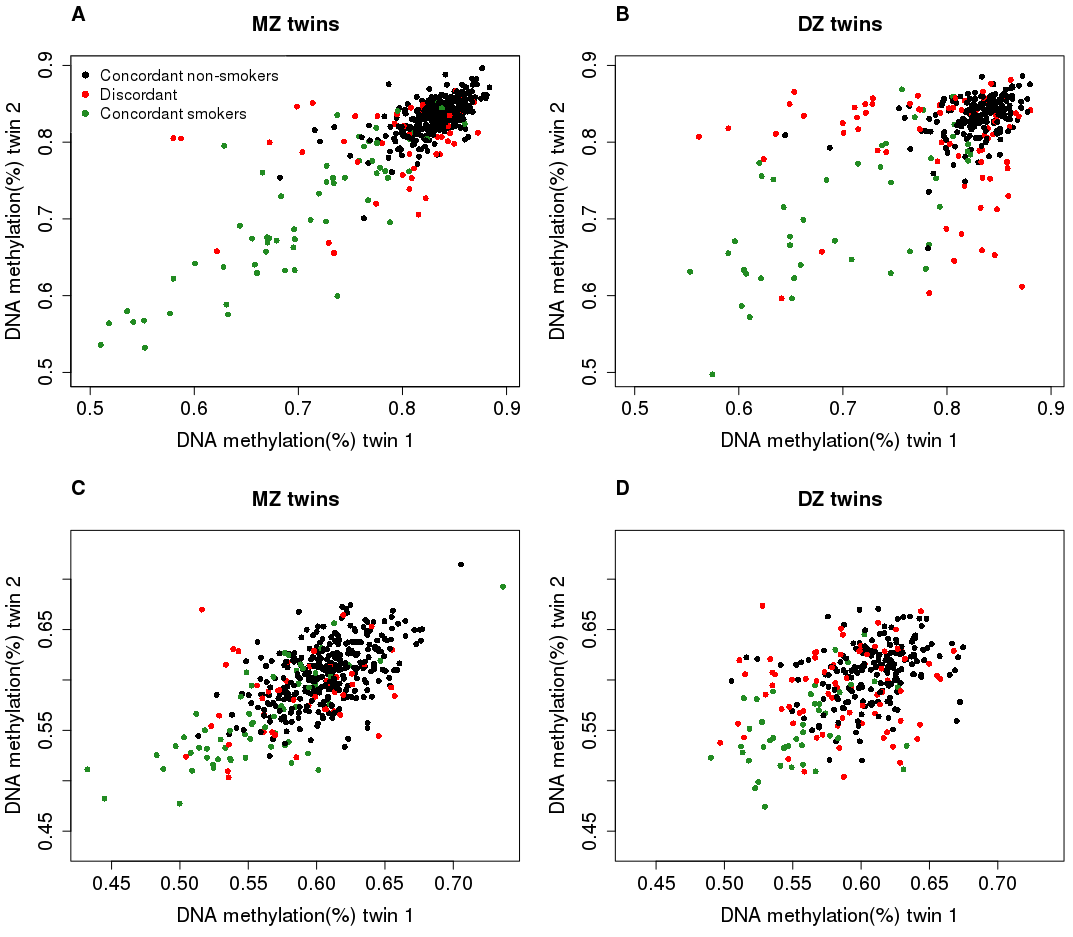

Supplement: S25 Fig — Scatterplot of DNA methylation values at cg05575921 for A) monozygotic (MZ) twin pairs and B) dizygotic (DZ) twin pairs, and cg26703534 for C) MZ twin pairs and D) DZ twin pairs. Colors depict the concordance for current smoking status in each twin-pair. (TIF) [file pgen.1007544.s030.tif]

A

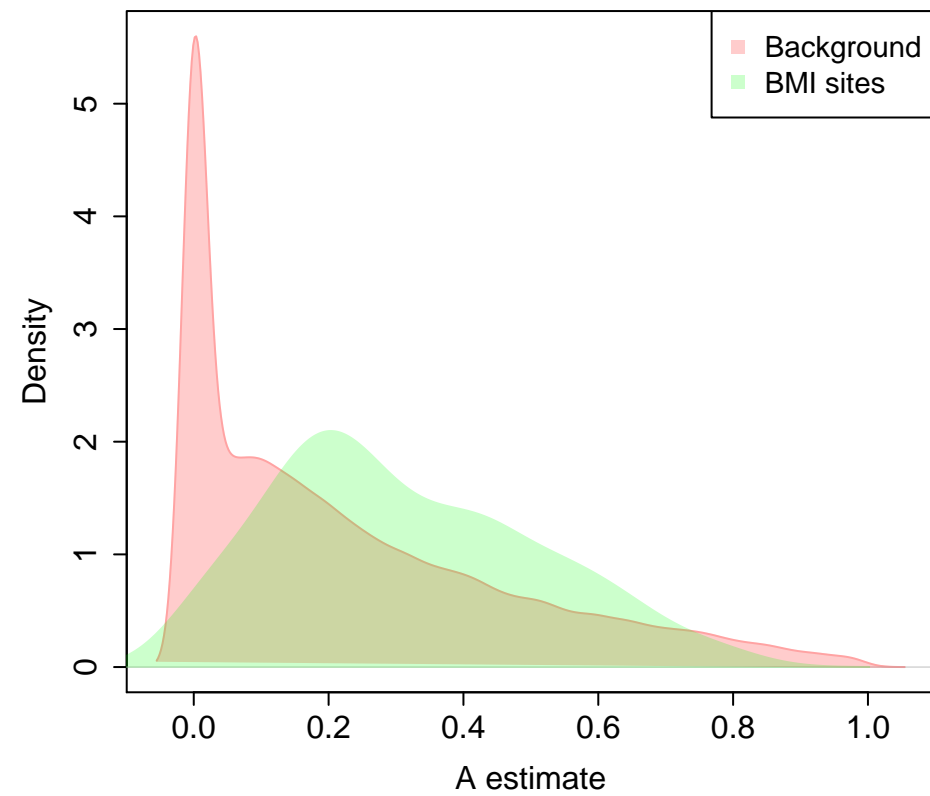

B

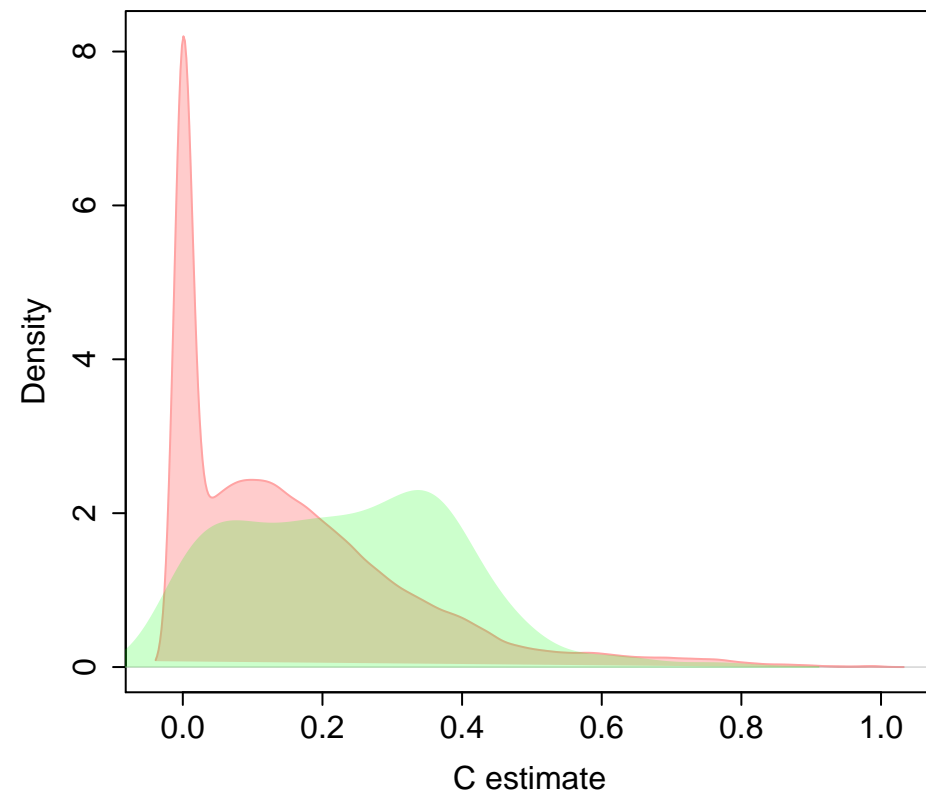

C

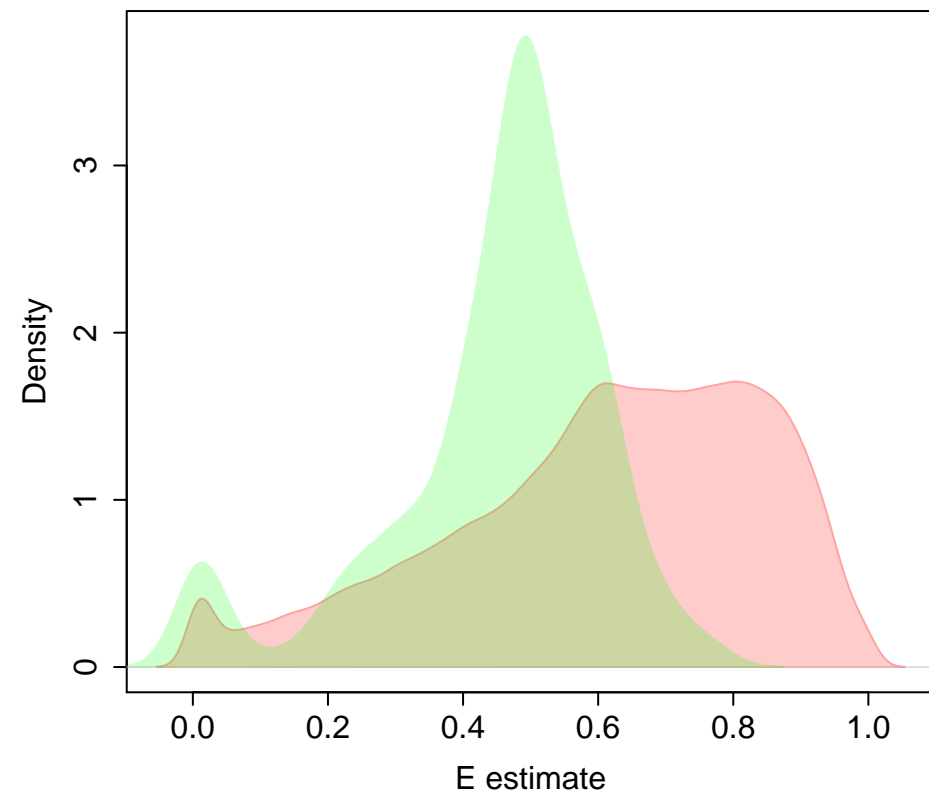

Supplement: S26 Fig — Density plots for estimates of A) additive genetic effects (A), B) shared environmental effects (C), and C) non-shared environmental effects (E) at 176 differentially methylated positions (DMPs) recently associated with BMI (green)[47]. (PDF) [file pgen.1007544.s031.pdf]
